# Disrupting the phase separation of KAT8–IRF1 diminishes PD-L1 expression and promotes antitumor immunity

**DOI:** 10.1038/s43018-023-00522-1

**Published:** 2023-03-09

**Authors:** Yuanzhong Wu, Liwen Zhou, Yezi Zou, Yijun Zhang, Meifang Zhang, Liping Xu, Lisi Zheng, Wenting He, Kuai Yu, Ting Li, Xia Zhang, Zhenxuan Chen, Ruhua Zhang, Penghui Zhou, Nu Zhang, Limin Zheng, Tiebang Kang

**Affiliations:** 1 Sun Yat-sen University Cancer Center, State Key Laboratory of Oncology in South China, Collaborative Innovation Center for Cancer Medicine, Guangzhou, China; 2 Department of Neurosurgery, First Affiliated Hospital of Sun Yat-sen University, Guangzhou, People’s Republic of China

**Keywords:** Gene regulation, Acetylation, Tumour immunology, Cancer therapy, Cancer

## Abstract

Immunotherapies targeting the PD-1/PD-L1 axis have become first-line treatments in multiple cancers. However, only a limited subset of individuals achieves durable benefits because of the elusive mechanisms regulating PD-1/PD-L1. Here, we report that in cells exposed to interferon-γ (IFNγ), KAT8 undergoes phase separation with induced IRF1 and forms biomolecular condensates to upregulate PD-L1. Multivalency from both the specific and promiscuous interactions between IRF1 and KAT8 is required for condensate formation. KAT8–IRF1 condensation promotes IRF1 K78 acetylation and binding to the *CD274* (PD-L1) promoter and further enriches the transcription apparatus to promote transcription of PD-L1 mRNA. Based on the mechanism of KAT8–IRF1 condensate formation, we identified the 2142–R8 blocking peptide, which disrupts KAT8–IRF1 condensate formation and consequently inhibits PD-L1 expression and enhances antitumor immunity in vitro and in vivo. Our findings reveal a key role of KAT8–IRF1 condensates in PD-L1 regulation and provide a competitive peptide to enhance antitumor immune responses.

## Abstract (web summary)

Wu et al. report that IFNγ induces the phase separation of KAT8 and IRF1 into condensates that promote PD-L1 upregulation. They further identify a peptide that blocks KAT8–IRF1 condensates, reducing PD-L1 levels and increasing antitumor immunity.

## Main

PD-L1 expressed by tumor cells has been demonstrated to be a dominant suppressor of antitumor immune surveillance^1,2^. When engaging with PD-1 on T cells, PD-L1 induces inhibition of cytotoxic T cell proliferation and subsequent exhaustion and apoptosis of these cells^3,4^. Within cells, PD-L1 exerts multiple functions to promote tumor immune evasion, such as increasing the resistance of tumor cells to interferon (IFN) cytotoxicity^5^, enhancing DNA damage repair^6^ and promoting the expression of immunosuppressive genes^7,8^. Targeting PD-L1 has become one of the most promising treatments for individuals with cancer, especially those with late-stage disease. Thus, fully understanding how PD-L1 is regulated helps in designing new targeting strategies for cancer immune therapy.

Molecular assemblies are increasingly being found to form membraneless biomolecular condensates in a phase separation-dependent manner^9,10^. Biomolecular condensates are involved in a broad range of physiological processes and have also been reported to control cancer-related dysregulation^11^. Investigating the mechanism of biomolecular condensate formation in detail may present opportunities to develop effective targeting strategies, as targeting phase separation processes in tumor cells seems to produce clinical benefits^12,13^. Although some progress has been made in disrupting phase separation with kinase inhibitors^14,15^, it is still challenging, especially for condensates formed by intrinsic disordered regions (IDRs), which lack defined, stable three-dimensional (3D) structures^16^, suggesting that a detailed investigation of the condensate formation process is needed to bring new insights into the development of phase separation-targeting drugs.

Here, we performed whole-genome CRISPR–Cas9 gene knockout screens and found that the histone acetyltransferase KAT8 transcriptionally upregulates PD-L1 via cocondensation with IRF1. Mechanistically, after cell exposure to IFNγ, KAT8 first binds to amino acids 21–42 in the N-terminal DNA-binding domain (DBD) of induced IRF1, and the IDRs of the two proteins promote KAT8–IRF1 condensate formation. Meanwhile, KAT8 acetylates IRF1 at K78 to promote its DNA binding, which synergizes with H4K16 acetylation to enhance the transcription of PD-L1 mRNA (encoded by *CD274*). Based on the condensate formation mechanism, we developed a cell-penetrating blocking peptide 2142–R8, which disrupted KAT8–IRF1 condensates, inhibited PD-L1 expression and enhanced antitumor immunity in vitro and in vivo.

## Results

### CRISPR–Cas9 screening identified KAT8 as a PD-L1 regulator

**Fig. 1:**
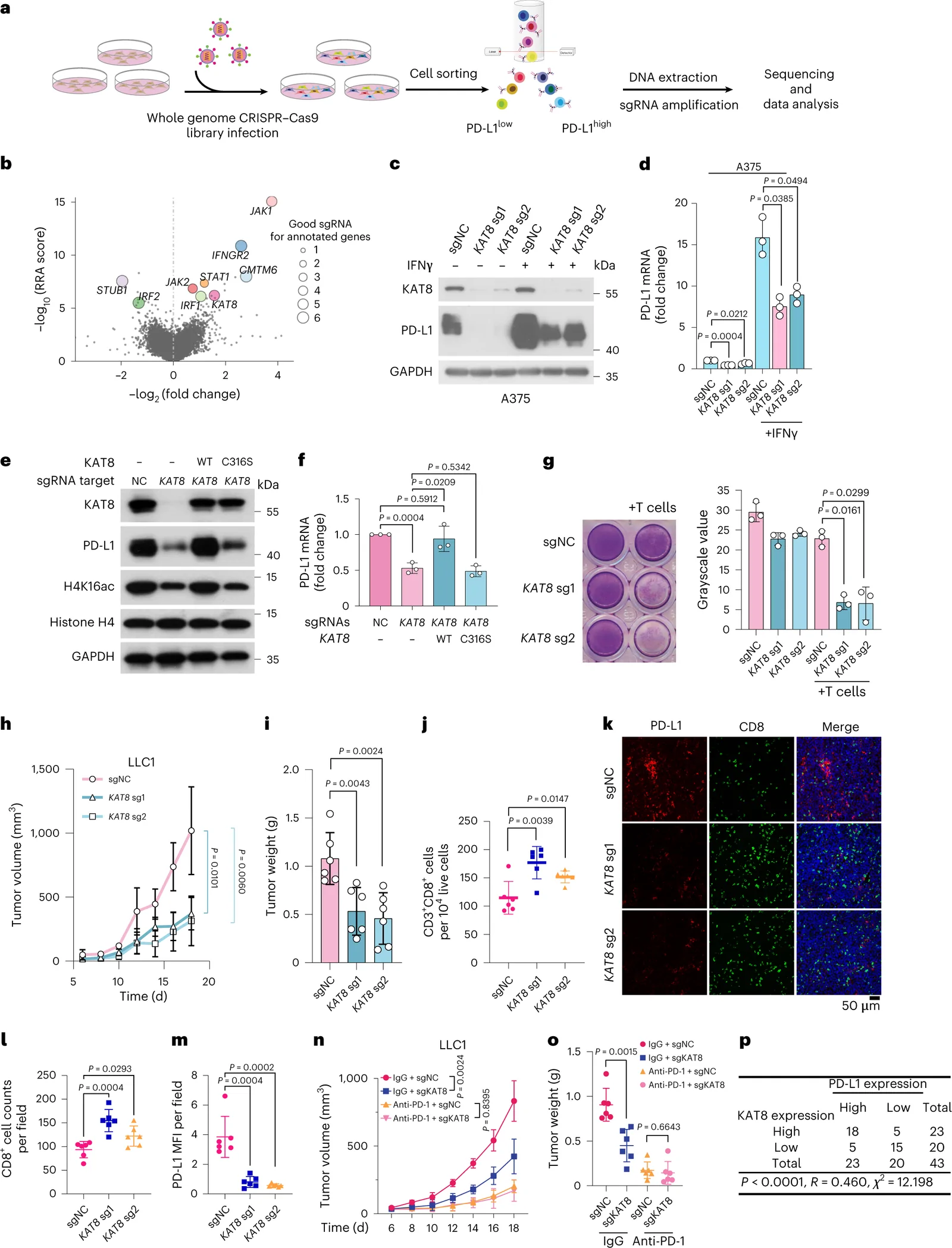
CRISPR–Cas9 screening identified KAT8 as a PD-L1 regulator. **a**, Schematic of the experimental design. **b**, Plot of whole-genome CRISPR–Cas9 gene knockout screen results using MAGeCK analysis. Cells were sorted after 14 d of infection. The *x* axis indicates the fold change of each gene, the *y* axis shows the Robust Rank Aggregation (RRA) score of each gene, and the bubble size of the indicated genes indicates the number of good sgRNAs. **c**,**d**, Western blotting (**c**) and quantitative PCR with reverse transcription (RT–qPCR) (**d**) analyses of A375 cells expressing the indicated sgRNAs in the presence or absence of 100 U ml^–1^ IFNγ for 6 h; *n* = 3 biologically independent experiments. **e**,**f**, A375 cells expressing the indicated sgRNAs were infected with sgRNA-resistant WT KAT8 or the C316S mutant, as indicated, and analyzed by western blotting (**e**) and RT–qPCR (**f**); *n* = 3 biologically independent experiments. **g**, Cytotoxicity assay of A375 cells expressing the indicated sgRNAs; *n* = 3 biologically independent experiments. **h**–**m**, LLC1 cells expressing the indicated sgRNAs were subcutaneously injected into mice. Tumor growth (**h**) and tumor weights (**i**) were measured, and the extent of CD3^+^CD8^+^ T cell infiltration was analyzed by FACS (**j**). Tumor slices were stained with anti-PD-L1 and anti-CD8 (**k**), and CD8^+^ cell counts (**l**) and mean fluorescence intensity (MFI) of PD-L1 (**m**) were analyzed. Data in **h**–**m** were generated from *n* = 6 mice for each group. **n**,**o**, Mice bearing tumors formed by LLC1 cells expressing sgNC and sgKAT8 were treated with IgG or anti-PD-1 on days 4, 6 and 8. Tumor sizes were measured at the indicated time points (**n**). Weights of the resected tumors were measured at the endpoint (**o**); *n* = 6 mice per group. Error bars in **d**, **f**–**j** and **l**–**o** indicate the mean ± s.d. *P* values in **h** and **n** were calculated by two-way ANOVA with Tukey’s multiple-comparison test. *P* values in **d**, **f**, **g**, **i, j**, **l**, **m** and **o** were calculated by two-tailed Student’s *t-*test. **p**, Crosstab shows the distribution of cancer tissues in the human multiple organ cancer tissue arrays according to the median IHC score of PD-L1 and KAT8. The *P* value and chi-square value were calculated using Pearson’s chi-square test, and the *R* value was calculated using Spearman’s correlation test. Source data

**Extended Data Fig. 1:**
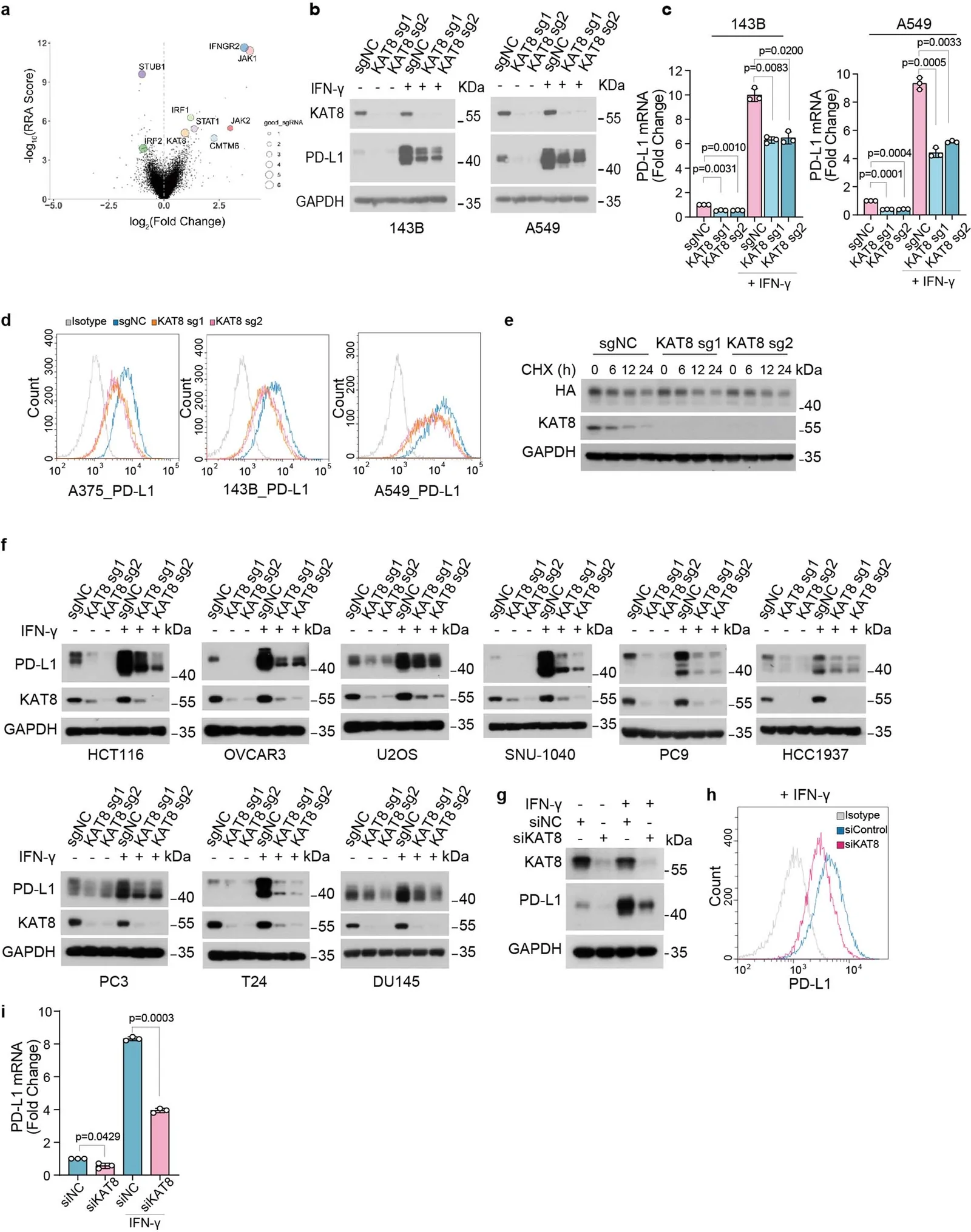
KAT8 is a PD-L1 transcriptional regulator. **a**, Plot of the whole-genome CRSIPR-Cas9 gene knockout screen result using MAGeCK analysis. The cells were sorted after 7 days of infection. The X axis indicates the fold change of each gene, the Y axis shows the robust ranking score of each gene, the bubble size of the indicated genes indicates the number of good sgRNAs. **b**, Western blot analysis of PD-L1 in 143B (left) and A549 (right) cells with sgNC or sgRNAs targeting KAT8 with or without 100 U/mL IFN-γ treatment for 6 h. **c**, qRT-PCR analysis of PD-L1 mRNA levels in cells in (**b**). **d**, Flow cytometric analysis of cell surface PD-L1 in A375, 143B and A549 cells with sgNC or sgRNAs targeting KAT8 after 100 U/mL IFN-γ treatment for 6 h. **e**, 143B cells expressing sgNC or sgRNAs targeting KAT8 were transfected with PD-L1-HA vector for 24 h, after that, cells were treated with cycloheximide (20 μg/mL) for indicated time. Then cells were lysed and analyzed by western blot. **f**, Western blot analysis of PD-L1 expression in the indicated cell lines expressing sgNC or sgRNAs targeting KAT8 with or without 100 U/mL IFN-γ treatment for 6 h. **g**-**i**, Western blot (**g**), flow cytometric analysis (**h**) and qRT-PCR (**i**) analysis of PD-L1 in A375 cells transfected with siNC or mixture of siRNAs targeting KAT8 for 72 h with or without 100 U/mL IFN-γ exposure for 6 h. Only cells treated with IFN-γ were subject to flow cytometric analysis. Data in **c**,**i** are shown as mean ± SD, n = 3 biologically independent experiments. *p* values in **c**,**i** were calculated by two-tailed Student’s *t*-test. The experiments in **b**,**d**-**h** were repeated three times with similar results. Source data

Considering that IFNγ secreted by T cells has been demonstrated to be a profound modifier of the tumor microenvironment^17,18^ and one of the key and strongest inducers of PD-L1 (ref. ^19^), we used whole-genome CRISPR–Cas9 gene knockout screens to identify the regulators of PD-L1 expression in tumor cells after IFNγ exposure in an unbiased manner. Well-established genes encoding key regulators of PD-L1, such as *JAK1*, *IFNGR2*, *STAT1*, *IRF2* and the recently reported *CMTM6* and *STUB1* (refs. ^20,21^), were enriched among the top-ranked genes. Interestingly, the gene encoding the histone acetyltransferase KAT8 was one of the most significantly enriched genes in our screens (Fig. 1a,b, Extended Data Fig. 1a and Supplementary Tables 1 and 2). As the major lysine acetyltransferase catalyzing histone H4 lysine 16 acetylation (H4K16ac) in mammalian cells^22,23^, KAT8 can also acetylate non-histone proteins^24–27^ and plays important roles in various cellular processes, including autophagy^28^, the stress response^29^ and nucleus–mitochondria communication^30^. However, the role of KAT8 in tumor progression and how KAT8 regulates the tumor immune microenvironment remain poorly defined.

Ectopic expression of Cas9 and single guide RNAs (sgRNAs) targeting *KAT8* in multiple cell lines (osteosarcoma cell line 143B, malignant melanoma cell line A375 and lung cancer cell line A549) significantly decreased the total protein and mRNA levels of PD-L1 with or without IFNγ exposure (Fig. 1c,d and Extended Data Fig. 1b,c). Cell surface PD-L1 levels were also decreased (Extended Data Fig. 1d). Moreover, depleting KAT8 did not affect PD-L1 protein half-life (Extended Data Fig. 1e). These results suggest that KAT8 regulates PD-L1 mRNA transcription in various cancer cell lines. Further, reduced PD-L1 expression induced by KAT8 depletion was observed in an extensive set of cell lines (Extended Data Fig. 1f). Importantly, wild-type (WT) KAT8, but not its C316S catalytically deficient mutant, rescued the downregulation of PD-L1 and H4K16ac in KAT8-depleted cells, indicating that the acetyltransferase activity of KAT8 is critical for regulation of PD-L1 expression (Fig. 1e,f). Likewise, treatment of cells with small interfering RNA targeting *KAT8* resulted in decreased PD-L1 expression (Extended Data Fig. 1g–i).

**Extended Data Fig. 2:**
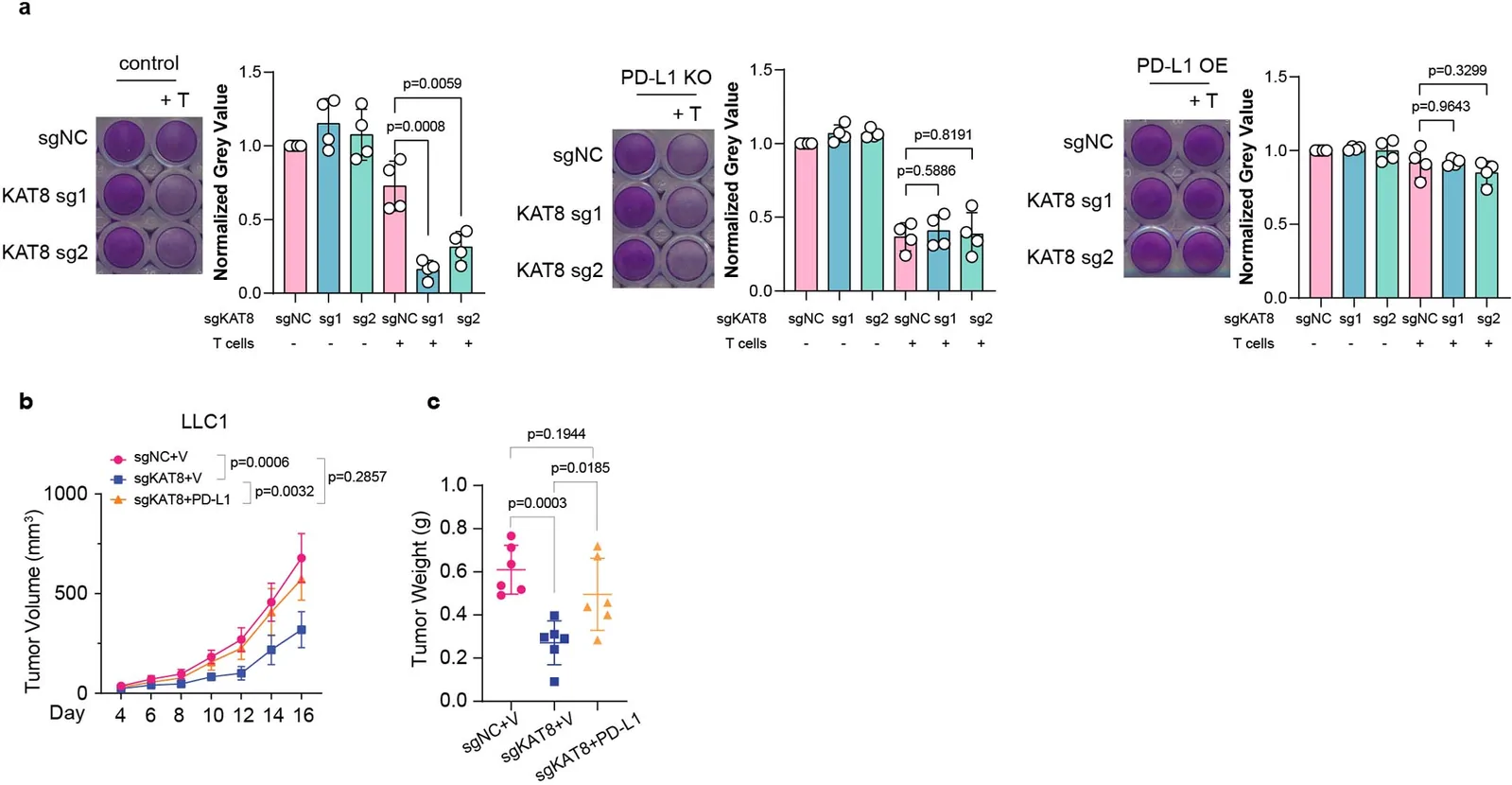
The function of KAT8 on immune regulation relies on PD1-PD-L1 axis. **a**, Wild type (control), PD-L1 knockout (KO) and PD-L1 overexpression (OE) A375 cells were transfected with sgNC or sgRNAs targeting KAT8. Then cells were cocultured with activated T cells from human PBMCs in 48-well plates for 24 h. Then, the T cells were carefully washed away with PBS, and the adherent tumor cells were stained with crystal violet. n = 4 biologically independent experiments. *p* values were calculated by two-tailed Student’s *t*-test. Error bars indicate the mean ± SD. **b**,**c**, The indicated stable expression LLC1 cell lines were subcutaneously injected into mice. Tumor sizes were measured at the indicated time points (**b**), and the endpoint tumor weights were measured (**c**). n = 6 mice per group. *p* values in (**b**) were calculated by two-way ANOVA with Tukey multiple comparisons test. *p* values in (**c**) were calculated by two-tailed Student’s *t*-test. Error bars indicate the mean ± SD. Source data

Next, we examined whether the downregulation of PD-L1 expression by KAT8 depletion impacts the antitumor response. In vitro cytotoxicity assays showed that depleting KAT8 significantly enhanced T cell killing (Fig. 1g), while overexpression of PD-L1 reversed the enhancement (Extended Data Fig. 2a). No further T cell killing enhancement after KAT8 depletion was observed in *CD274*-knockout cells (Extended Data Fig. 2a). In vivo, KAT8 depletion in the Lewis lung carcinoma cell line LLC1 inhibited tumor growth and reduced tumor weight (Fig. 1h,i) while increasing the tumor infiltration of CD3^+^CD8^+^ T cells in mice (Fig. 1j–m). Moreover, the antitumor effect of KAT8 depletion could be reversed by ectopic PD-L1 expression (Extended Data Fig. 2b,c). KAT8 depletion could not further retard tumor growth in mice treated with anti-PD-1 (Fig. 1n,o). These data suggest that depletion of KAT8 enhances antitumor immunity via the PD-L1/PD-1 axis in vitro and in vivo. Moreover, 33 of 43 (76.74%) samples from human multiple organ cancer tissue arrays showed synchronized high or low expression levels of KAT8 and PD-L1 as detected by immunohistochemistry (IHC; Fig. 1p and Supplementary Table 3), indicating that there is a positive correlation between KAT8 and PD-L1 in human cancers.

### KAT8 interacts and forms dynamic condensates with IRF1

**Fig. 2:**
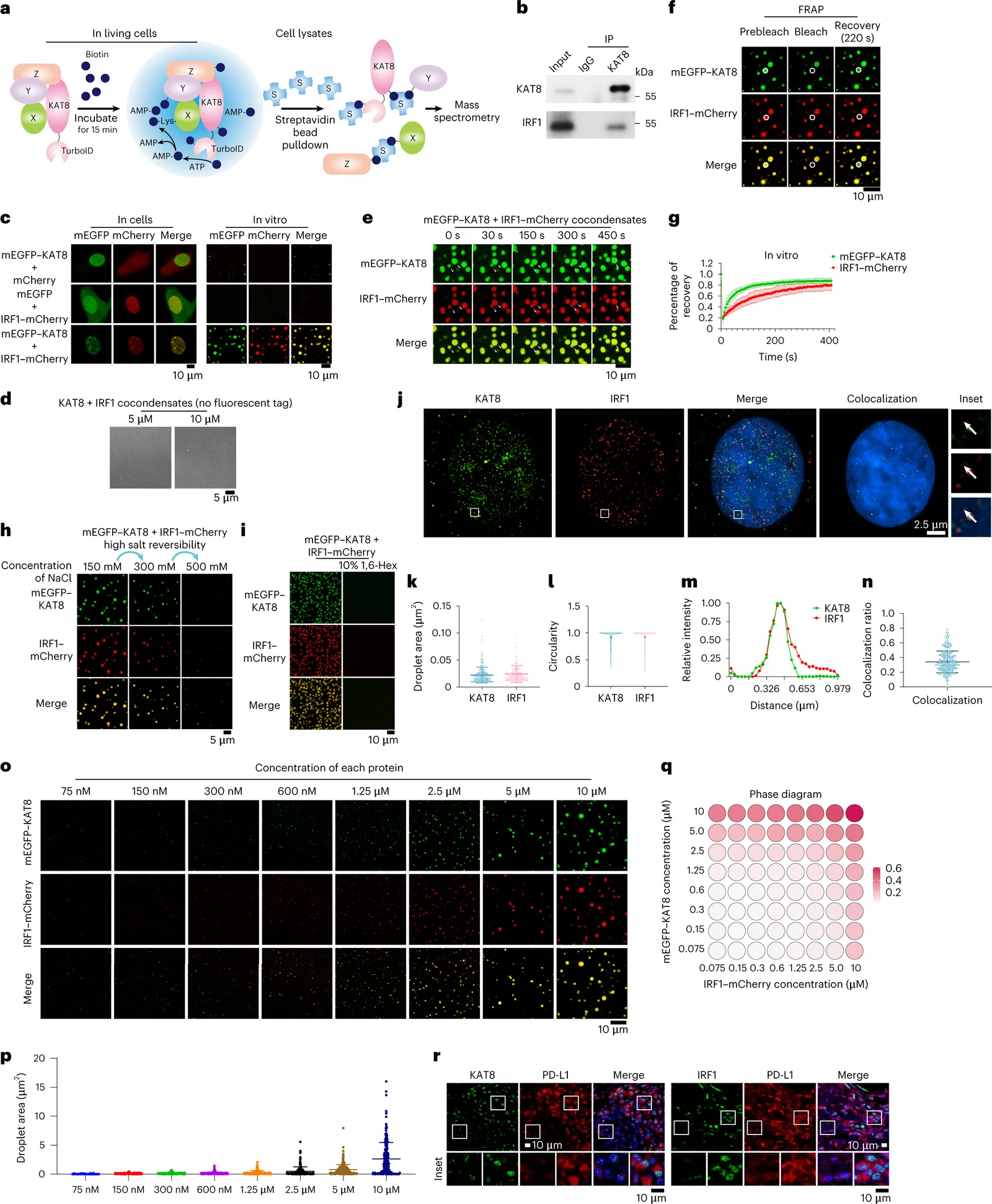
KAT8 interacts and forms dynamic condensates with IRF1, which is correlated with PD-L1 expression. **a**, Schematics of the proximity labeling system using V5-TurboID-tagged KAT8. **b**, Endogenous IRF1 was immunoprecipitated with endogenous KAT8 in A375 cells treated with 100 U ml^–1^ IFNγ for 6 h; IP, immunoprecipitation. **c**, Condensate formation was analyzed in 143B cells transfected with the indicated constructs (left). Droplet formation was analyzed in the indicated protein mixture (10 μM each) at room temperature in the presence of 150 mM NaCl and 10% PEG 8000 (right). **d**, Differential interference contrast microscopy images of purified KAT8 and IRF1 proteins without fluorescent protein tags at room temperature. **e**, Arrows point to the representative droplets formed by mEGFP–KAT8 and IRF1–mCherry that fused over time. **f**,**g**, FRAP assay of the droplets formed by mEGFP–KAT8 and IRF1–mCherry; *n* = 6 biologically independent experiments. **h**, Reversibility of mEGFP–KAT8–IRF1–mCherry (10 μM each) droplets in response to treatment with high NaCl concentrations at room temperature. **i**, Droplets formed by 50 μM recombinant mEGFP–KAT8 and IRF1–mCherry with or without 10% 1,6-hexanediol (1,6-Hex) treatment in the presence of 100 mM NaCl without crowding agent. **j**–**m**, SIM analysis of endogenous KAT8 and IRF1 localization in A375 cells after treatment with 100 U ml^–1^ IFNγ for 6 h. Hoechst 33342 was used to stain nuclei. Colocalization of the two proteins is shown as yellow dots. Images (**j**), droplet area (**k**), circularity (**l**) and line scan analysis (**m**) are shown; *n* = 287 puncta for KAT8 and *n* = 236 puncta for IRF1. **n**, Colocalization ratio defined by IRF1–KAT8 colocalized area/IRF1 total area in each cell; *n* = 232 cells. **o**,**p**, Condensate formation was analyzed in the mEGFP–KAT8 and IRF1–mCherry mixtures at the indicated concentrations of each protein at room temperature (**o**). The areas of 250 random droplets in **o** were quantified and plotted in **p**. **q**, Phase diagrams of the mEGFP–KAT8 and IRF1–mCherry mixtures at the indicated concentrations for each protein at room temperature measured at an optical density at 600 nm. **r**, Tissue samples from individuals with lung cancer were costained with anti-PD-L1 and either anti-KAT8 or anti-IRF1 as indicated. The experiments shown in **b**–**e**, **h**–**j**, **o**, **q** and **r** were repeated independently three times with similar results. Error bars in **g**, **k**, **n** and **p** indicate the mean ± s.d. Source data

**Extended Data Fig. 3:**
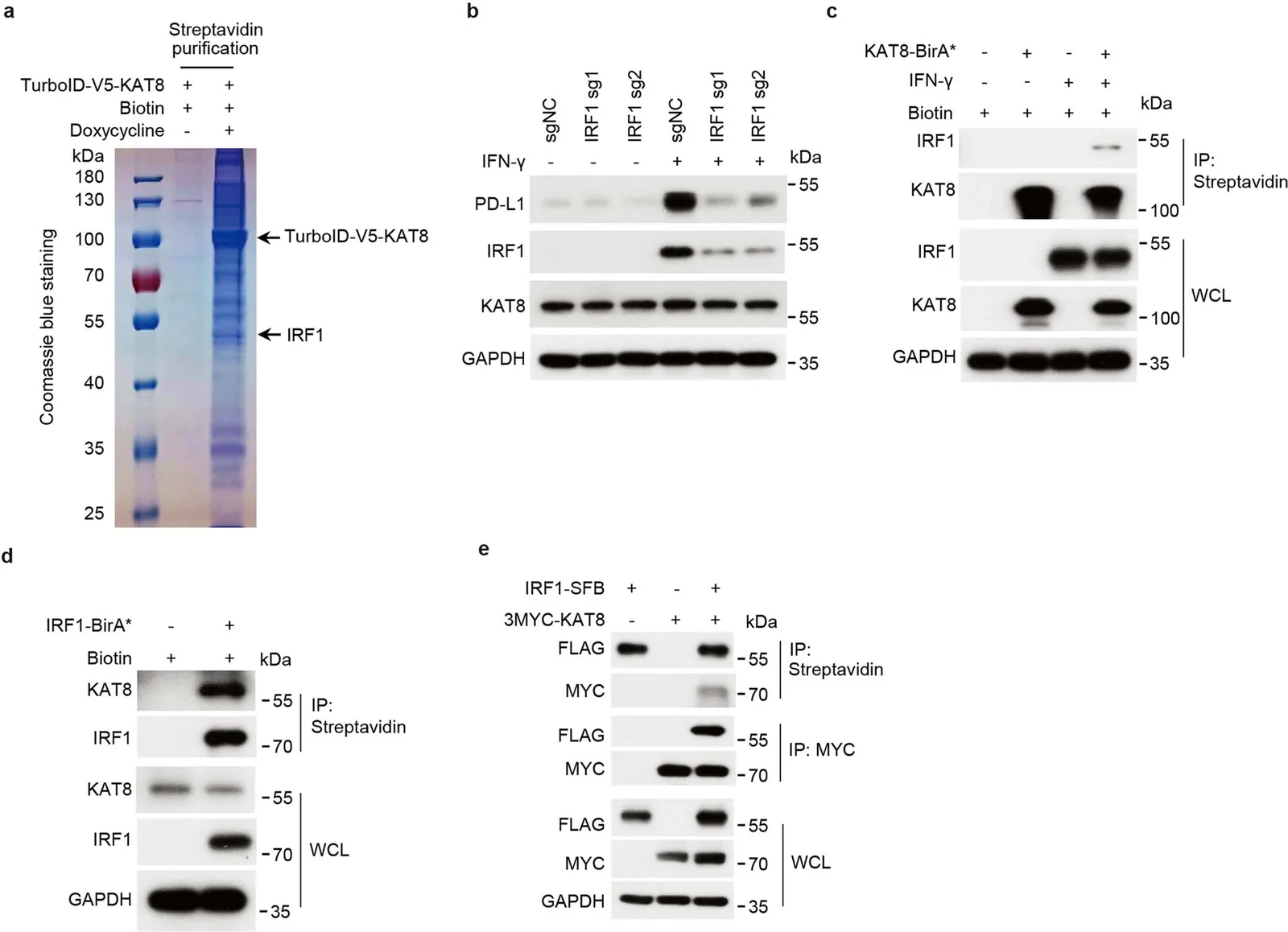
KAT8 interacts with IRF1. **a**, Coomassie blue staining of an SDS-PAGE gel showing the proteins from the nuclear lysates of A375 cells expressing doxycycline-inducible V5-TurboID-KAT8 that pulled down with the streptavidin beads before subject to mass spectrometry analysis. See Methods for details of proximity labeling assay. **b**, Western blot analysis of PD-L1 expression in A375 cells expressing sgRNAs targeting IRF1 with or without 100 U/mL IFN-γ treatment for 6 h. **c**,**d**, A375 cells expressing BirA* (BirA R118G mutation)-fused KAT8 (**c**) or IRF1 (**d**) were treated with or without 100 U/mL IFN-γ in the presence of 50 μM biotin for 24 h. Cell lysates were immunoprecipitated with streptavidin beads and blotted with the indicated antibodies. **e**, HEK293T cells were transfected with the indicated constructs for 24 h. The cell lysates were immunoprecipitated with streptavidin or anti-MYC beads and blotted with the indicated antibodies. The experiment in **a** was performed once. The experiments in **b**-**e** were repeated three times with similar results. Source data

To investigate how KAT8 regulates PD-L1 mRNA transcription, we applied a proximity labeling method by introducing TurboID-tagged KAT8 into A375 cells to label potential interacting proteins^31^ (Fig. 2a). Using mass spectrometry, we identified IRF1 as one of the labeled proteins (Extended Data Fig. 3a and Supplementary Table 4), which has been reported as the main transcription factor in the IFNγ pathway that induces PD-L1 mRNA transcription^19^. Indeed, cells expressing sgRNAs targeting *IRF1* showed substantial inhibition in the induction of PD-L1 expression by IFNγ (Extended Data Fig. 3b). Spatial colocalization of KAT8 and IRF1 was confirmed by the BirA* proximity labeling method^32^ (Extended Data Fig. 3c,d). The interaction between KAT8 and IRF1 was further confirmed by immunoprecipitations at endogenous and exogenous levels (Fig. 2b and Extended Data Fig. 3e).

**Extended Data Fig. 4:**
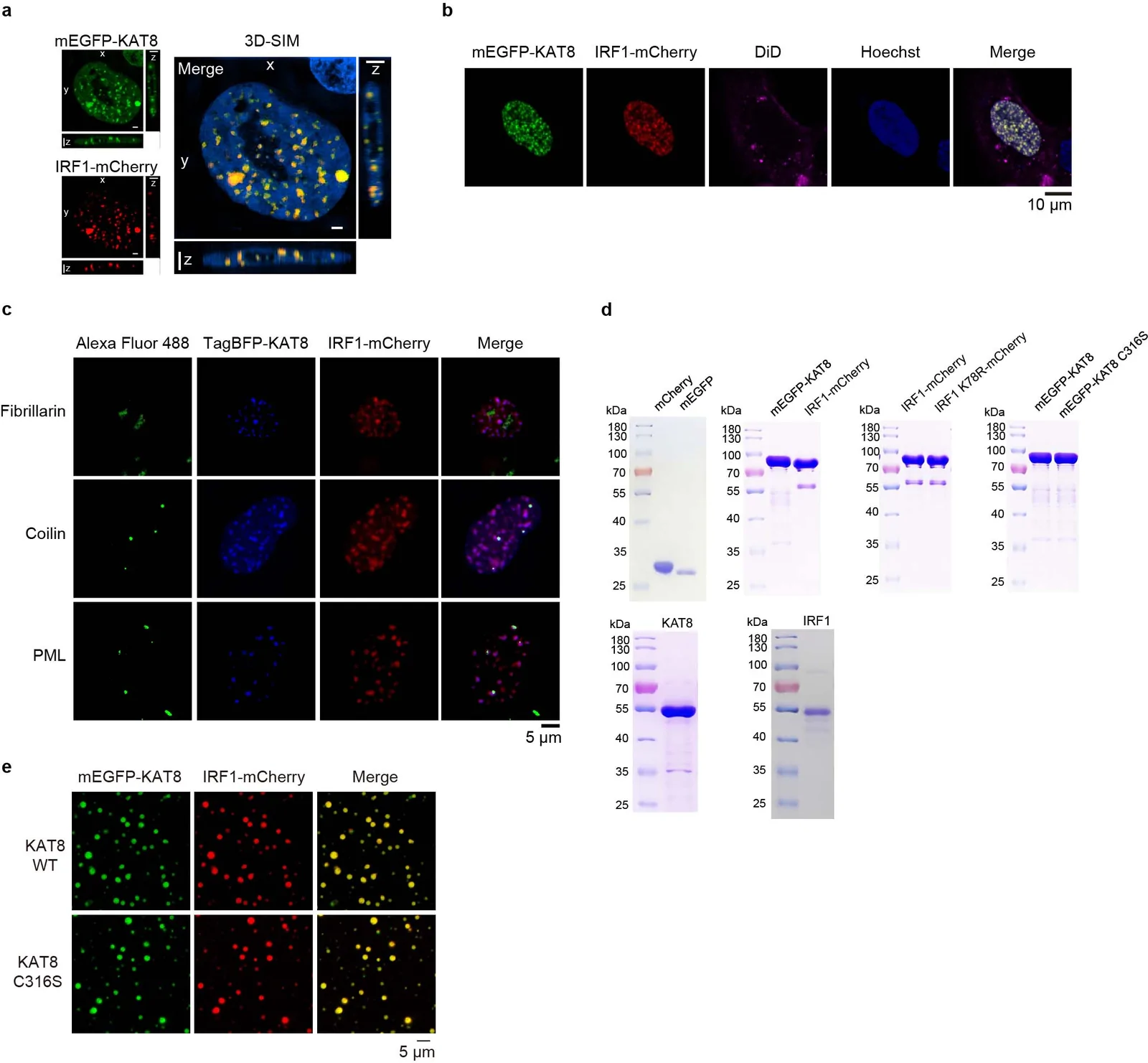
KAT8-IRF1 forms membrane-less condensates. **a**, Structured illumination microscopy (SIM) analysis of mEGFP-KAT8 and IRF1-mCherry localization in 143B cells. **b**, Live-cell images of 143B cells expressing mEGFP-KAT8 and IRF1-mCherry with DiD staining. **c**, 143B cells expressing TagBFP-KAT8 and IRF1-mCherry were stained with anti-fibrillarin, anti-coilin or anti-PML antibodies. **d**, Indicated purified proteins used for in vitro LLPS assays were analyzed by SDS-PAGE and stained with Coomassie blue. **e**, Droplet formation was analyzed in purified mEGFP-KAT8 WT/C316S, IRF1-mCherry at room temperature in the presence of 150 mM NaCl and 10% PEG 8000. The concentration of each protein was 10 μM. Hoechst 33342 was used for staining nuclei. Scale bars in **a**,**c**,**e** indicate 5 μm. Scale bar in **b** indicates 10 μm. The experiments in **a**-**e** were repeated three times with similar results.

**Extended Data Fig. 5:**
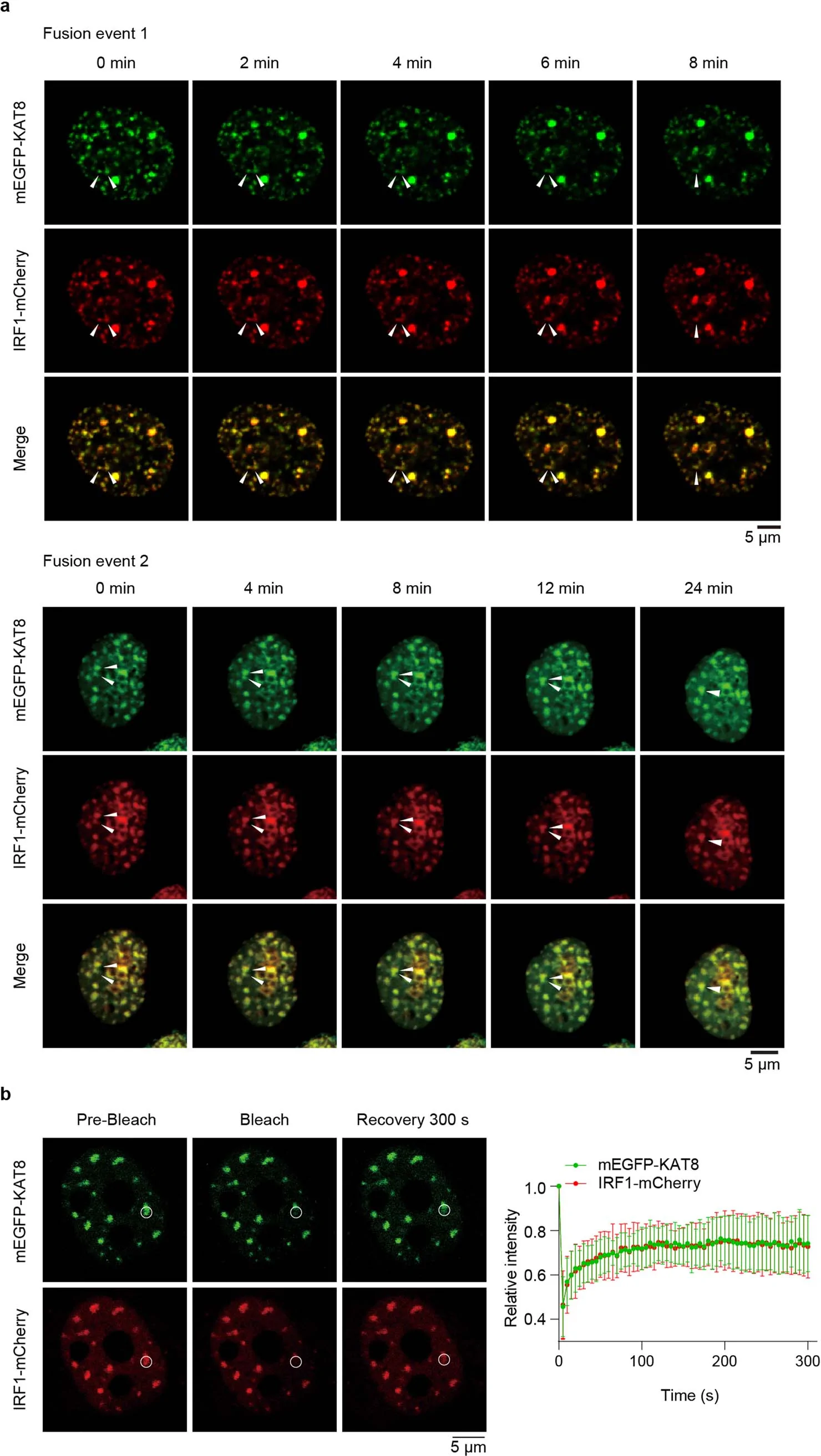
Intracellular fusion and FRAP of KAT8-IRF1 condensates. **a**, Live cell images of 143B cells expressing mEGFP-KAT8 and IRF1-mCherry. The arrows point to the representative condensates formed by KAT8 and IRF1 that fused over time. **b**, FRAP assay of the droplets formed by mEGFP-KAT8 and IRF1-mCherry. The data are plotted as the mean ± SD on the right, n = 5 biologically independent experiments. Source data

Cells transfected with monomeric enhanced green fluorescent protein–KAT8 (mEGFP–KAT8) and IRF1–mCherry showed droplet-like condensates in the nuclei (Fig. 2c, left). Three-dimensional (3D) structured illumination microscopy (3D-SIM) reconstruction also showed droplet-like structures (Extended Data Fig. 4a). These condensates were negative for the lipid marker dye DiD (Extended Data Fig. 4b), did not colocalize with the nucleolus and partially colocalized with Cajal bodies and PML bodies (Extended Data Fig. 4c), indicating that the condensates were membraneless structures. We then examined the dynamic properties of KAT8–IRF1 condensates. Purified mEGFP–KAT8 or IRF1–mCherry alone showed a weak capacity for droplet formation, while mixing both together dramatically enhanced droplet formation (Fig. 2c, right, and Extended Data Fig. 4d). Droplet formation was not dependent on fluorescent protein tags nor KAT8 acetyltransferase activity (Fig. 2d and Extended Data Fig. 4e). Moreover, droplets were able to fuse over time and partially recovered after photobleaching (fluorescence recovery after photobleaching (FRAP)) in vitro and in cells (Fig. 2e–g and Extended Data Fig. 5a,b); these droplets could be disrupted by 1,6-hexanediol and high concentrations of NaCl (Fig. 2h,i). Collectively, these results indicate the dynamic and reversible properties of KAT8–IRF1 condensates.

**Extended Data Fig. 6:**
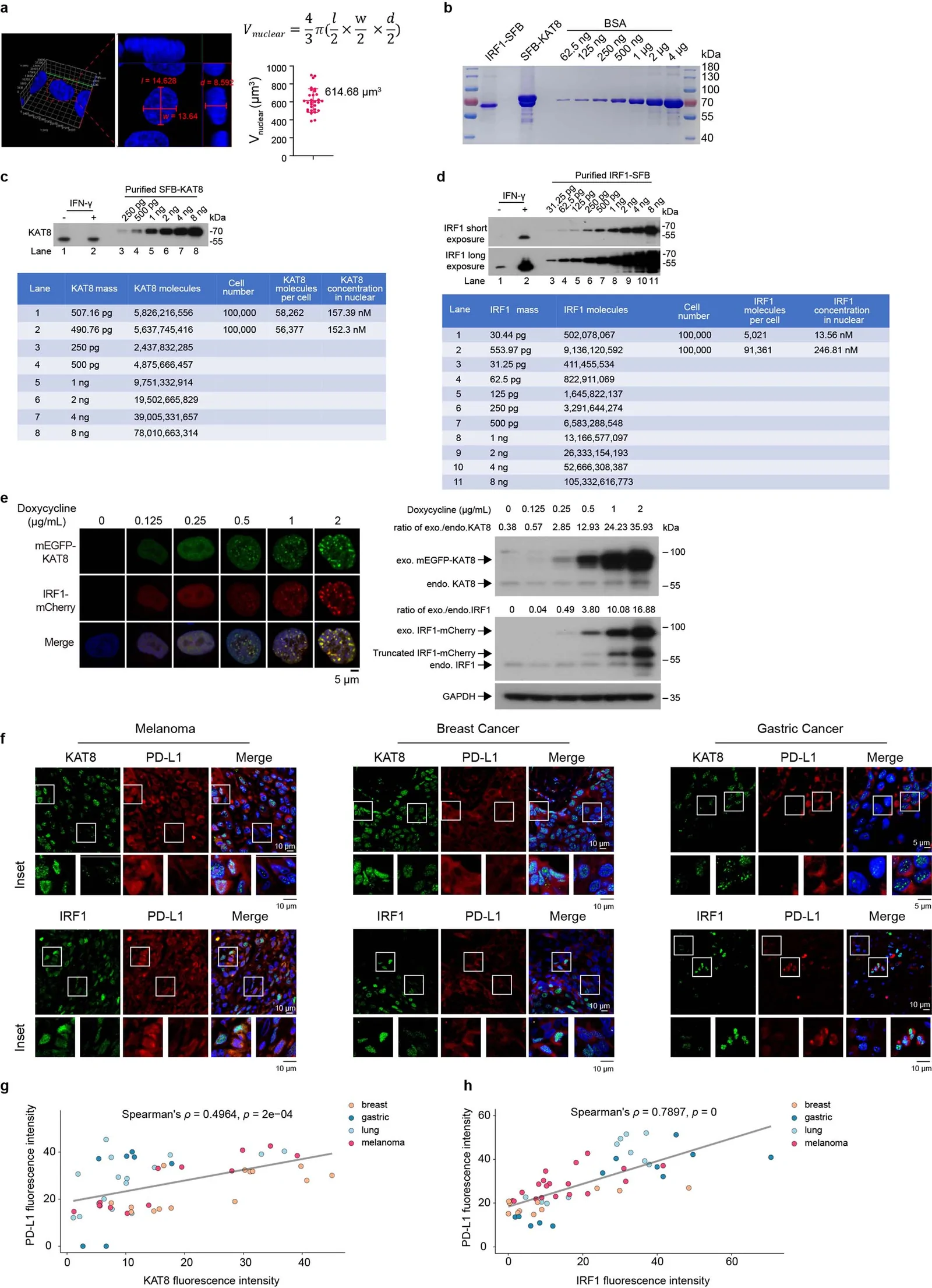
Calculation of KAT8 and IRF1 endogenous protein concentrations and the staining of KAT8 or IRF1 in patient samples. **a**, Representative 3D reconstruction of confocal image of 143B cells stained with Hoechst 33342. The nuclear volumes were calculated by the ellipsoid volume formula indicated on top of the plot. A total of 30 randomly selected cells were measured and analyzed. The statistical data were plotted on the right. Error bar indicates the mean ± SD. **b**, SFB-KAT8 and IRF1-SFB were expressed in HEK293T cells and purified by streptavidin beads. Coomassie blue staining were applied to validate the protein purity and were quantified by Quantity One. Protein concentrations were calculated by the standard curve generated from Bovine Serum Albumin (BSA) protein. **c**,**d**, Quantification of endogenous KAT8 (**c**) and IRF1 (**d**). Quantified SFB-KAT8 and IRF1-SFB were used to generate standard curves to estimate the endogenous KAT8 and IRF1 protein numbers from 100, 000 143B cells with or without IFN-γ 100 U/mL for 12 h. Western blot signals were quantified by Quantity One. Protein concentrations were estimated by the protein numbers per cell divided by the mean nuclear volume. **e**, pTeton-mEGFP-KAT8 and IRF1-mCherry were integrated into the genome of 143B cells by transposase (see Method for details). A concentration gradient of doxycycline from 0.125 to 2 μg/mL were used to induce mEGFP-KAT8 and IRF1-mCherry expressions for 12 hours in the presence of IFN-γ 100 U/mL, then cells were fixed and visualized by confocal microscope (on the left side), proteins were harvested and detected by western blot (on the right side). **f**-**h**, Indicated patient tissue samples were stained with anti-PD-L1 antibodies and either anti-KAT8 or anti-IRF1 antibodies as indicated. Representative images are shown (**f**). The fluorescence intensities of KAT8, IRF1 and PD-L1 of each cell in the representative images were measured using Fiji. Data of KAT8 and PD-L1 (**g**), IRF1 and PD-L1 (**h**) are plotted. Spearman’s correlation coefficients (ρ) and the two-sided *p* values are shown. The fitted linear regression lines are also depicted in grey. The experiments in **b**-**e** were repeated three times with similar results. The experiments in **f** were repeated twice with similar results. Source data

Superresolution imaging showed that endogenous KAT8 and IRF1 formed small condensates in cells, and colocalized condensates were observed (Fig. 2j–n). Moreover, KAT8–IRF1 droplets formed in vitro when the two protein concentrations were higher than 75 nM (Fig. 2o–q); these concentrations were comparable to the endogenous expression levels of KAT8 and IRF1 (152.3 nM for KAT8 and 246.81 nM for IRF1 after IFNγ stimulation), as determined by quantitative western blotting^33^ and 3D reconstruction of cell nuclei (Extended Data Fig. 6a–d). Further, we observed concentration-dependent condensate formation of exogenous mEGFP–KAT8 and IRF1–mCherry in cells via a doxycycline-inducible expression system (Extended Data Fig. 6e). More importantly, in samples from individuals with cancer (lung cancer, melanoma, breast cancer and gastric cancer), KAT8 and IRF1 condensates were also observed, and the fluorescence intensities of both proteins were positively correlated with PD-L1 expression (Fig. 2r and Extended Data Fig. 6f–h). Together, these results demonstrate that KAT8 and IRF1 can form condensates in vivo and in vitro.

### KAT8–IRF1 condensation depends on multivalent interactions

**Fig. 3:**
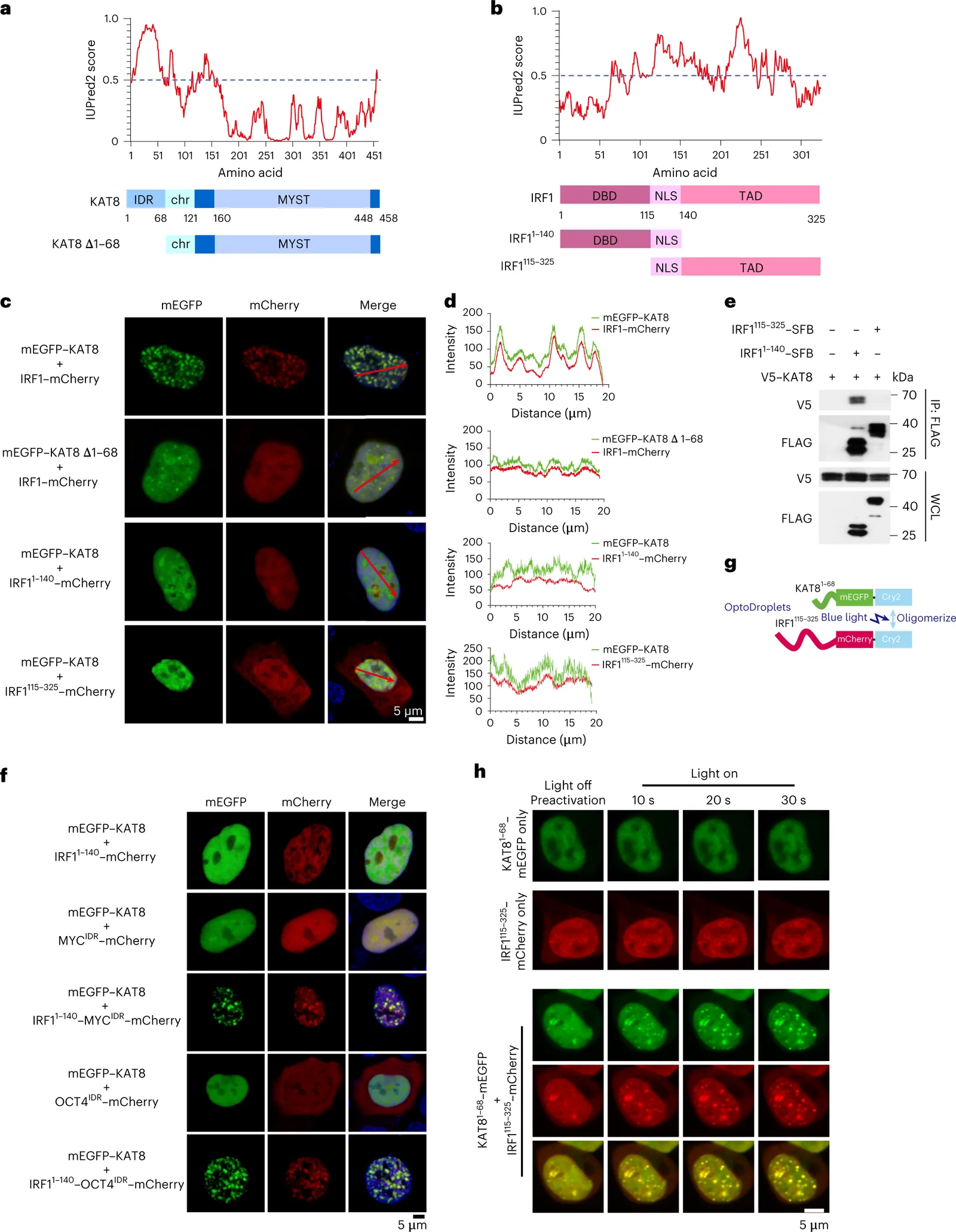
KAT8–IRF1 condensation depends on both structured domain and IDR interactions. **a**,**b**, Protein structure and IDR analysis of KAT8 (**a**) and IRF1 (**b**) using the IUPred2A tool with default parameters. Scores above 0.5 indicate disorder. The representative diagrams indicating the major domains of full-length KAT8 and IRF1 and the truncated constructs are shown below; TAD, transactivation domain; NLS, nuclear localization signal; chr, chromodomain; MYST (Moz, Ybf2/Sas3, Sas2, Tip60) acetyltransferase domain. **c**,**d**, Confocal microscopy images of condensate formation in 143B cells cotransfected with the indicated constructs (**c**). Line scan analysis results of fluorescence intensity along the indicated lines in **c** are shown (**d**). **e**, HEK293T cells were cotransfected with V5–KAT8 and the indicated IRF1–SFB truncated constructs. SFB-tagged proteins were immunoprecipitated with anti-FLAG beads and immunoblotted with anti-V5 or anti-FLAG; WCL, whole-cell lysate. **f**, Confocal microscopy images of representative 143B cells cotransfected with mEGFP–KAT8 and the indicated constructs. **g**, Schematic illustration of the optoDroplets assay. **h**, HEK293T cells were transfected with IRF1^115–325^–mCherry–Cry2 and KAT8^1–68^–mEGFP–Cry2 separately or together. Images were collected at the indicated times after illumination by a 488-nm laser. The experiments in **c**, **e**, **f** and **h** were repeated three times with similar results. Source data

To explore the structural basis of KAT8–IRF1 condensates, we analyzed the amino acid sequences of the two proteins using IUPred2 (ref. ^34^). Amino acids 1–68 of KAT8 (KAT8^1–68^) and IRF1^140–325^ (which includes the transactivation domain^35^) were scored as IDRs (Fig. 3a,b). Depletion of KAT8^1–68^ or IRF1^140–325^ disrupted condensate formation (Fig. 3c,d), indicating that the predicted IDRs contribute to this process. However, coimmunoprecipitation experiments showed that IRF1^1–140^ (containing the N-terminal DBD (amino acids 1–115)^36^), but not the predicted IDR of IRF1, was responsible for the KAT8 interaction (Fig. 3e), and deletion of the IRF1 DBD (that is the IRF1^115–325^ fragment) also impaired condensate formation with KAT8 (Fig. 3c,d), indicating that the DBD of IRF1 mediates interactions with KAT8 and is also required for condensate formation. Furthermore, fusing IRF1^1–140^ with IDRs from the unrelated transcription factors MYC or OCT4 restored condensate formation with KAT8, whereas IDR constructs alone from MYC or OCT4 could not (Fig. 3f). An optoDroplet assay^37^ also showed that when the KAT8 and IRF1 IDRs were fused with Cry2, blue light induction enhanced condensate formation between the IDRs of the two proteins (Fig. 3g,h and Supplementary Videos 1–3). Collectively, these observations indicate a multivalent interaction model where the interaction between the IRF1 DBD and KAT8 might be a prerequisite for condensate initiation, and the weak promiscuous interactions between the IDRs of both IRF1 and KAT8 promote condensate formation.

### KAT8–IRF1 condensates promote PD-L1 mRNA transactivation

**Fig. 4:**
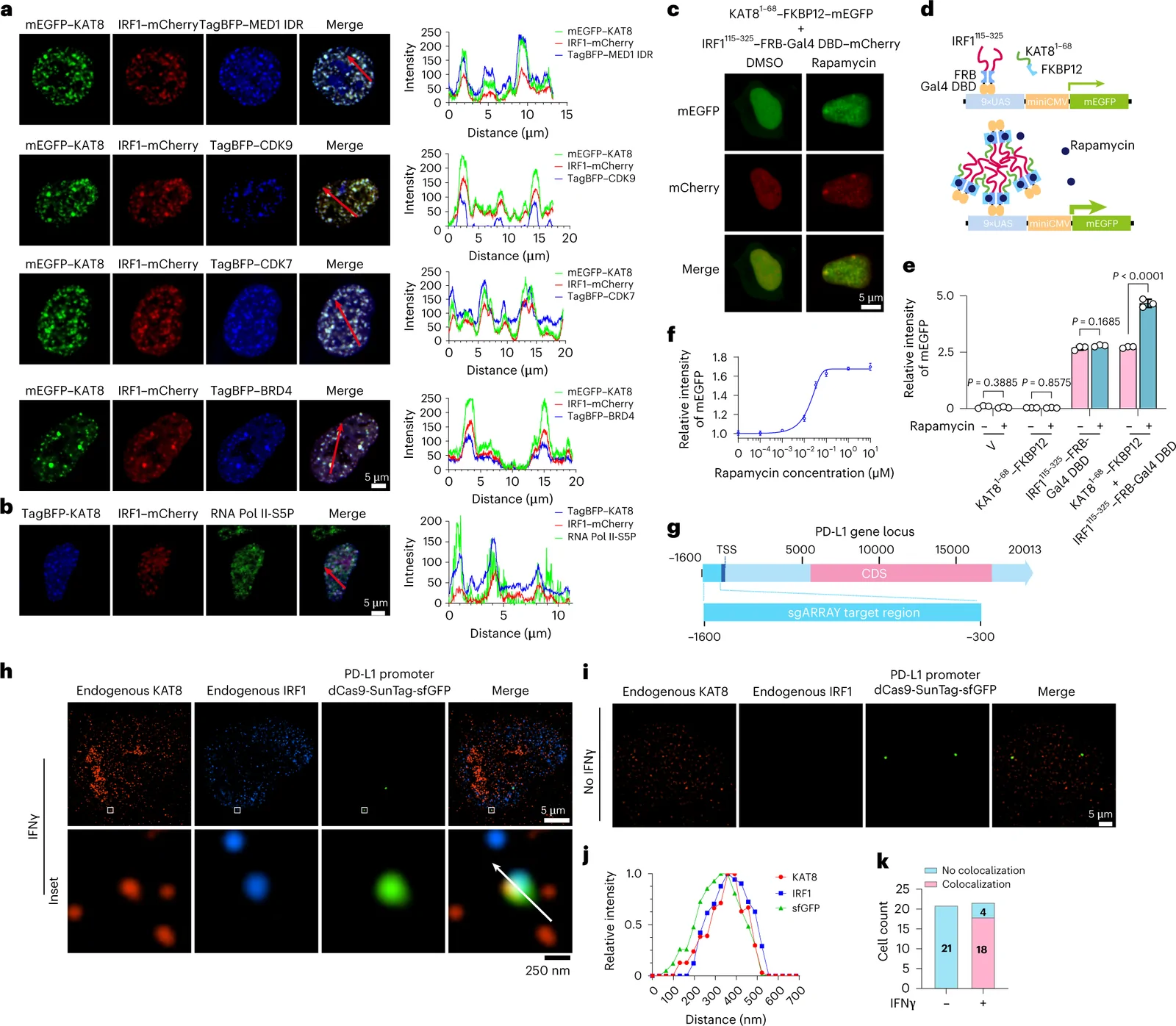
KAT8 and IRF1 condensates enrich the transcription apparatus and colocalize with the PD-L1 promoter to promote transactivation. **a**,**b**, Confocal microscopy images of representative condensates in 143B cells cotransfected with the indicated constructs (**a**) and stained with the indicated antibodies (**b**). The corresponding line scan analyses are plotted on the right. **c**, Confocal microscopy images of HEK293T cells with KAT8^1–68^–FKBP12–mEGFP and IRF1^115–325^–FRB-Gal4 DBD–mCherry cotransfected and treated with or without rapamycin (200 nM) for 1 h. **d**, Schematic illustration of the Gal4 reporter assay. **e**, 143B cells integrated with Gal4-UAS–mEGFP reporter were transfected with the indicated vectors and treated with DMSO or rapamycin (200 nM) for 18 h before analysis by FACS. **f**, IRF1^115–325^–FRB-Gal4 DBD and KAT8^1–68^–FKBP12 stably integrated UAS–mEGFP reporter cells were treated with the indicated concentrations of rapamycin for 24 h, and mEGFP signal intensity was quantified by FACS. **g**–**k**, In situ analysis of the colocalization of endogenous KAT8 and IRF1 with the PD-L1 promoter region by SIM; 143B cells were transfected with a set of dCas9-SunTag-sfGFP and sgARRAY vectors to visualize the PD-L1 promoter region (Methods). A schematic of the PD-L1 promoter region targeted by sgARRAY is shown (**g**). After treatment with (**h**) or without (**i**) IFNγ for 6 h, 143B cells were stained with anti-KAT8 and anti-IRF1. The corresponding line scan analyses are shown in **j**. Cells with positive or negative localization of KAT8–IRF1 droplets at the sfGFP sites were counted in the presence or absence of 100 U ml^–1^ IFNγ treatment for 6 h (**k**); TSS, transcription start site; CDS, coding sequence. *P* values in **e** were calculated by two-tailed Student’s *t*-tests. Error bars in **e** and **f** indicate the mean ± s.d.; *n* = 3 biologically independent experiments. The experiments in **a**–**c**, **h** and **i** were repeated three times with similar results. Source data

Next, we investigated whether KAT8–IRF1 condensates can enhance transcription. Transcriptional machinery components, including MED1 IDR, CDK7, CDK9, BRD4 and active RNA polymerase II phosphorylated at serine 5 (RNA Pol II-S5P), were enriched in KAT8–IRF1 condensates (Fig. 4a,b). To test the contribution of IDR-mediated KAT8–IRF1 condensation in transcription enhancement, we designed a rapamycin-inducible interaction system to uncouple the structured IRF1 DBD–KAT8 interaction and the promiscuous IDR interaction by fusing the KAT8 IDR or IRF1 IDR with FKBP12 or the FRB-Gal4 DBD. After rapamycin treatment, cells cotransfected with KAT8 IDR–FKBP12 and IRF1 IDR–FRB-Gal4 DBD showed small condensates in the nuclei (Fig. 4c), indicating that this system can simulate condensation induced by the KAT8 and IRF1 IDR interaction. We then evaluated the transactivation effect of IDR condensation using a Gal4 upstream activation site (UAS) reporter assay (Fig. 4d). Transfection of IRF1 IDR–FRB-Gal4 DBD increased reporter expression, while no-IDR control and KAT8 IDR–FKBP12 showed no activity, and rapamycin induction could not further enhance reporter expression. Cells cotransfected with KAT8 IDR–FKBP12 and IRF1 IDR–FRB-Gal4 DBD showed similar reporter expression levels as cells only transfected with IRF1 IDR–FRB-Gal4 DBD in the absence of rapamycin, while reporter expression was significantly enhanced after rapamycin treatment (Fig. 4e). Moreover, rapamycin dose–response curves displayed a nonlinear activation pattern in KAT8 IDR–FKBP12 and IRF1 IDR–FRB-Gal4 DBD stably integrated UAS reporter cells (Fig. 4f). These results suggest that IDR-mediated KAT8–IRF1 condensation promotes transactivation. Further, after performing dCas9-SunTag-sgARRAY-mediated in situ labeling^38^, we observed that endogenous KAT8 and IRF1 formed condensates at the PD-L1 promoter in cells (Fig. 4g–k).

**Extended Data Fig. 7:**
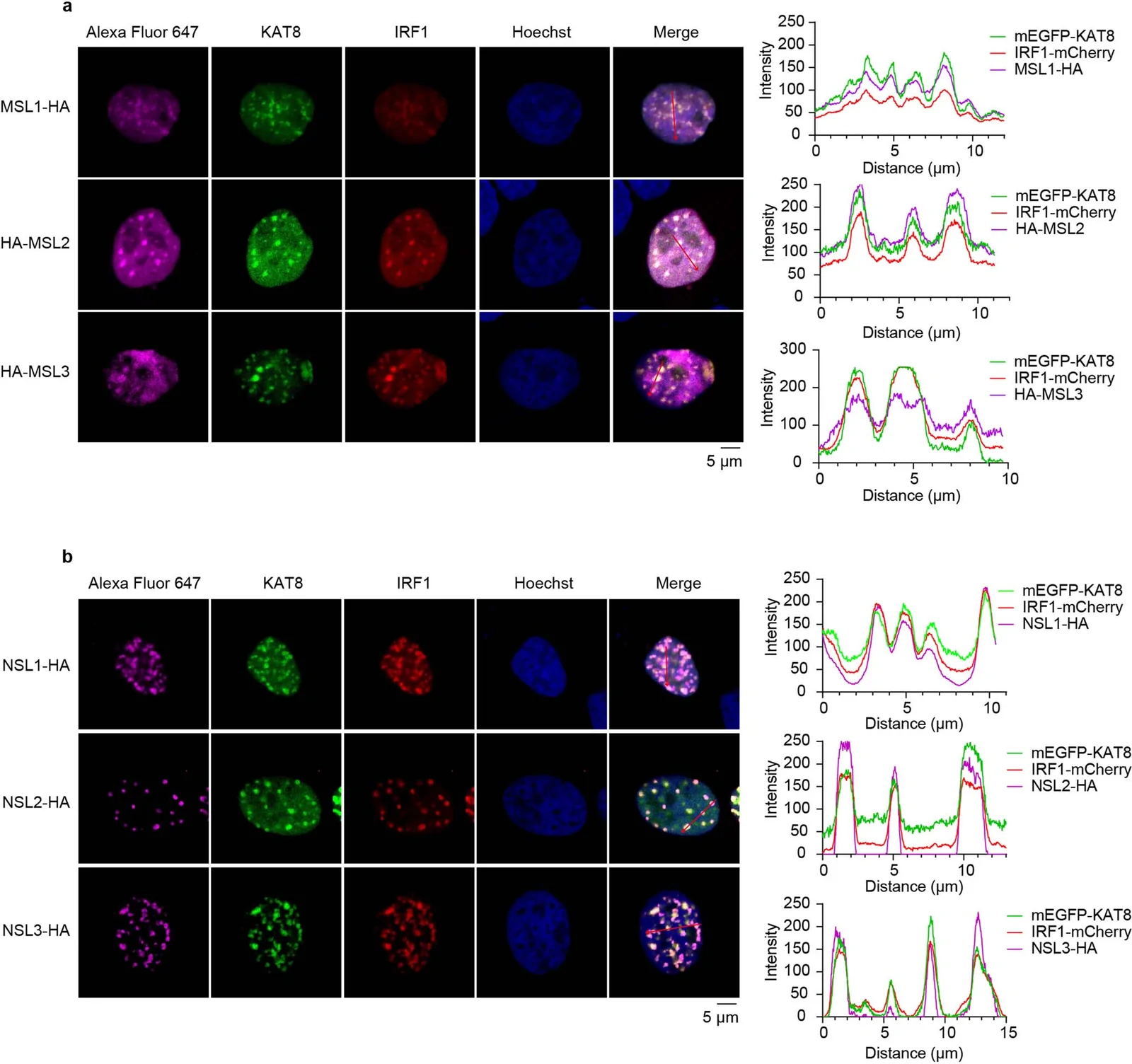
MSL and NSL complexes are involved in KAT8-IRF1 condensates. **a**,**b**, Confocal microscopy images of droplet formation in 143B cells cotransfected with mEGFP-KAT8, IRF1-mCherry and HA-tagged MSLs (**a**) or NSLs (**b**). Cells were stained with anti-HA antibodies and Alexa Fluor 647 conjugated secondary antibodies. Cell nuclei were stained by Hoechst 33342. The corresponding line scan analysis of the fluorescence intensity along the indicated line is shown on the right. The experiments were repeated three times with similar results. Source data

It has been shown that KAT8 serves as the catalytic core subunit in male-specific lethal (MSL) and non-specific lethal (NSL) complexes^39,40^, which have different catalytic activities on histone and non-histone substrates. Our data showed that both MSL and NSL complex subunits are also involved in KAT8–IRF1 condensates (Extended Data Fig. 7a,b).

### KAT8 acetylates and promotes IRF1 activity

**Fig. 5:**
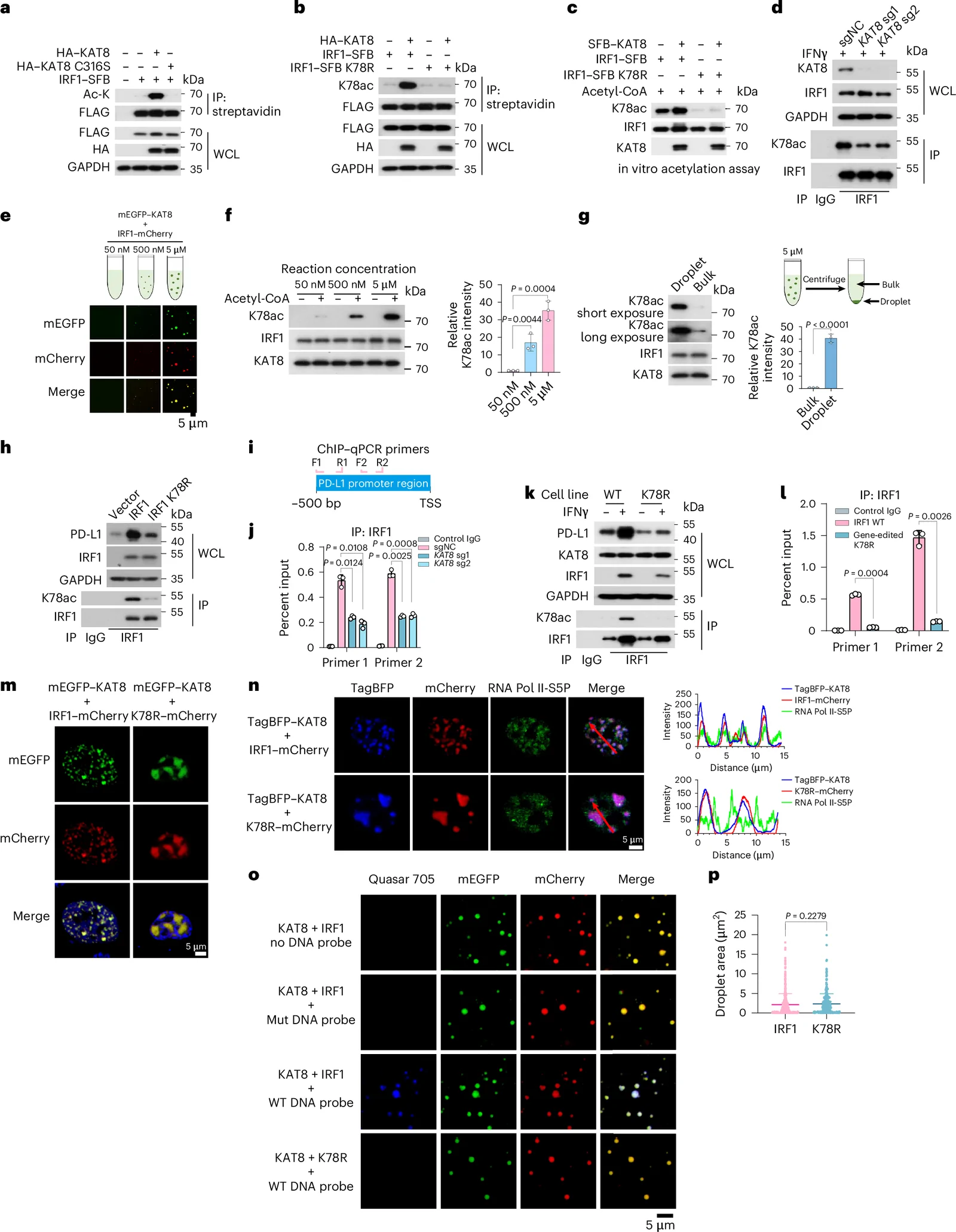
KAT8 acetylates IRF1 at K78 and promotes IRF1 binding to the PD-L1 promoter. **a**,**b**, HEK293T cells were cotransfected with the indicated constructs. Cell lysates were immunoprecipitated with streptavidin beads and immunoblotted with the indicated antibodies. **c**, In vitro acetylation assay using antibodies to IRF1 K78ac. **d**, Analysis of endogenous IRF1 K78ac in A375 cells in the presence of 100 U ml^–1^ IFNγ for 12 h. **e**–**g**, Droplet formation in the in vitro acetylation assay (**e**). The K78ac products were detected via western blotting and quantified (**f**). Bulk protein and protein from droplets from the reaction products were centrifuged and isolated for western blotting and quantification (**g**); *n* = 3 biologically independent experiments. **h**, Western blotting of PD-L1 and K78ac in A375 cells transfected with IRF1 WT or IRF1 K78R mutant. **i**, Schematic representing the amplicons of the two primers used for ChIP–qPCR at the PD-L1 promoter region. **j**, ChIP–qPCR analysis of IRF1 abundance at the PD-L1 promoter in A375 cells after treatment with 100 U ml^–1^ IFNγ for 12 h; *n* = 3 biologically independent experiments. **k**, Western blotting of IRF1 K78ac and PD-L1 in parental A375 cells and IRF1 K78R gene-edited A375 cells (K78R) with or without 100 U ml^–1^ IFNγ treatment for 6 h. **l**, ChIP–qPCR analysis of IRF1 abundance at the PD-L1 promoter in WT and K78R A375 cells after 100 U ml^–1^ IFNγ treatment for 12 h; *n* = 3 biologically independent experiments. **m**, Puncta formed by mEGFP–KAT8 with IRF1–mCherry or IRF1 K78R–mCherry in 143B cells. **n**, Representative confocal images showing the localization of TagBFP–KAT8, IRF1–mCherry/IRF1 K78R–mCherry and endogenous RNA Pol II-S5P in 143B cells; line scan analyses are shown on the right. **o**,**p**, Representative confocal microscopy images showing the overlap of mEGFP–KAT8, IRF1–mCherry/IRF1 K78R–mCherry and the indicated PD-L1 promoter-derived DNA probes Quasar 705 in vitro. Mut represents DNA probe with mutated IRF1 motifs (**o**). The droplet areas of mEGFP–KAT8 and IRF1–mCherry/IRF1 K78R–mCherry cocondensates in **o** in the presence of WT DNA probes were plotted (**p**); *n* = 519 droplets for IRF1; *n* = 425 droplets for K78R. Data in **f**, **g**, **j**, **l** and **p** are shown as the mean ± s.d., and *P* values were calculated by two-tailed Student’s *t-*test. The experiments in **a**–**h**, **k** and **m**–**o** were repeated three times with similar results. Source data

**Extended Data Fig. 8:**
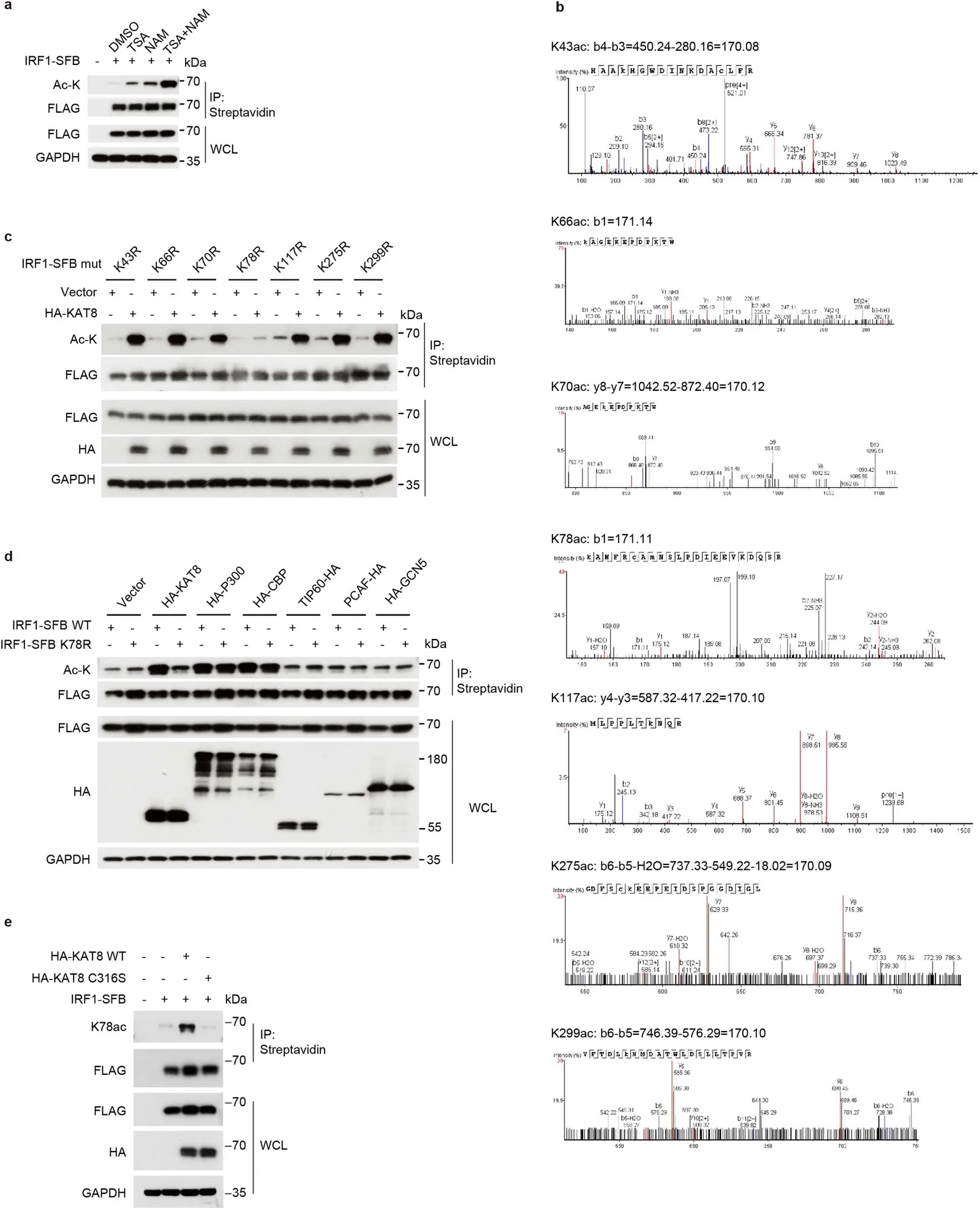
IRF1 K78 is acetylated by KAT8. **a**, HEK293T cells transiently transfected with IRF1-SFB for 24 h were treated with DMSO, 5 μM TSA, 5 mM NAM, or both compounds for another 24 h. Then, cell lysates were immunoprecipitated with streptavidin beads and immunoblotted with the indicated antibodies. **b**, The seven acetylated lysines identified by mass spectrometry were as follows: K43, K66, K70, K78, K117, K275, K299. **c**, HEK293T cells transiently cotransfected with vector or HA-KAT8 and the indicated mutants of IRF1-SFB for 48 h. Then, cell lysates were immunoprecipitated with streptavidin beads and immunoblotted with the indicated antibodies. **d**, HEK293T cells transiently cotransfected with IRF1 WT or K78R mutant and the indicated acetyltransferases for 48 h. Then, cell lysates were immunoprecipitated with streptavidin beads and immunoblotted with the indicated antibodies. **e**, HEK293T cells transiently cotransfected with IRF1-SFB and HA-KAT8 or HA-KAT8-C316S mutant as indicated for 48 h. Then, cell lysates were immunoprecipitated with streptavidin beads and immunoblotted with the indicated antibodies. The experiments in **a**,**c**-**e** were repeated three times with similar results. Source data

Next, we tested whether KAT8 can acetylate IRF1. IRF1 acetylation was detected and enhanced after treatment with the histone deacetyltransferase inhibitors trichostatin A (TSA) and nicotinamide (Extended Data Fig. 8a). Cotransfection of WT KAT8, but not its catalytically deficient C316S mutant, with IRF1 significantly enhanced the acetylation of IRF1 (Fig. 5a). By mass spectrometry analysis, we identified a total of seven acetylated lysine residues of IRF1 (K43, K66, K70, K78, K117, K275 and K299; Extended Data Fig. 8b and Supplementary Table 5). We then constructed IRF1 single-point mutants by replacing each identified acetylated lysine with arginine. Only the IRF1 K78R mutant did not show enhanced acetylation when cotransfected with WT KAT8 (Extended Data Fig. 8c). Importantly, IRF1 K78 acetylation (IRF1 K78ac) was specifically catalyzed by KAT8 but not by other histone acetyltransferases (Extended Data Fig. 8d). After application of a specific antibody to acetylated IRF1 K78 (K78ac), the direct catalytic activity of WT KAT8, but not the C316S mutant, on IRF1 K78 was further confirmed through acetylation assays in vivo and in vitro (Fig. 5b,c and Extended Data Fig. 8e). More importantly, depleting KAT8 resulted in decreased acetylation of endogenous IRF1 at K78 (Fig. 5d). Collectively, these data suggest that KAT8 specifically acetylates IRF1 K78.

Biomolecular condensate formation is used as a mechanism to increase enzymatic reaction rates, as enzymes and substrates are concentrated in the droplet^41–43^. To explore whether KAT8–IRF1 condensation promotes IRF1 K78ac, in vitro histone acetyltransferase activity assays with or without droplet formation were performed. IRF1 K78ac was substantially increased in droplets (Fig. 5e,f), and the acetylation ability of KAT8 in droplets was ~39.67 ± 1.997-fold higher than in bulk (Fig. 5g). These results provide evidence that KAT8 promotes IRF1 acetylation via cocondensation.

**Extended Data Fig. 9:**
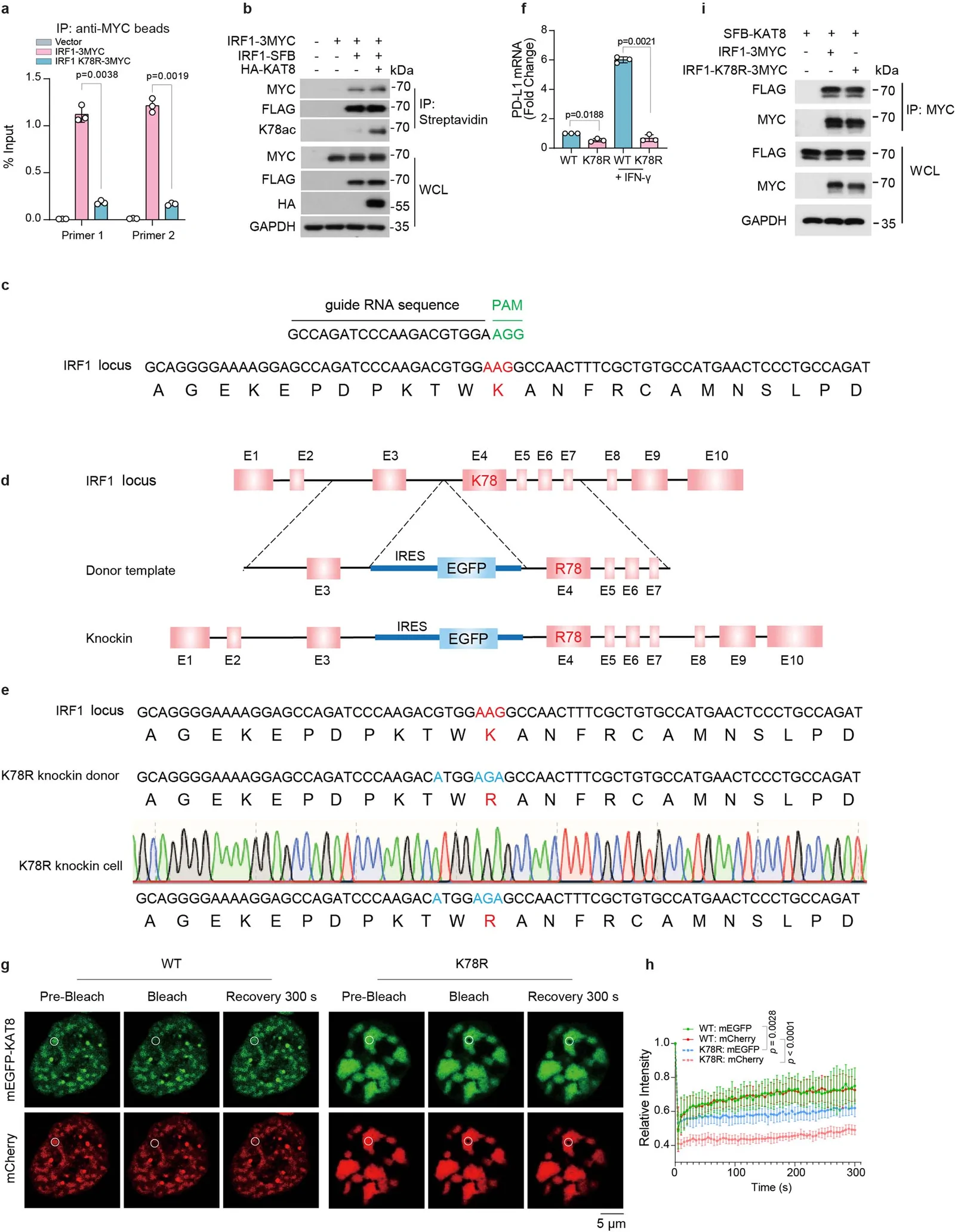
IRF1 K78 acetylation promotes the DNA binding of IRF1. **a**, ChIP-qPCR analysis of ectopically expressed IRF1-3MYC or IRF1 K78R-3MYC in HCT116 cells. Data are shown as the mean ± SD, n = 3 biologically independent experiments. *p* values were calculated by two-tailed Student’s *t*-test. **b**, HEK293T cells were transiently cotransfected with the indicated plasmids and analyzed by immunoprecipitation. **c**-**e**, Schematics of the locus-specific IRF1 K78R knock-in A375 cell line constructed by CRISPR-Cas9-mediated homology-directed repair. See Methods for details. An sgRNA efficiently targeting IRF1 was used (**c**). A DNA donor template containing internal ribosome entry site (IRES), EGFP, and indicated mutations sequence in the intron sequence between exon 3 and exon 4 was used (**d**). Site-specific PCR amplification and Sanger sequencing of the K78R gene-edited clone (**e**). **f**, qRT-PCR analysis of PD-L1 mRNA levels in parental WT and K78R A375 cells. Data are shown as the mean ± SD, n = 3 biologically independent experiments. *p* values were calculated by two-tailed Student’s *t*-test. **g**,**h** Representative confocal images showing FRAP of the puncta formed by mEGFP-KAT8 and IRF1-mCherry or IRF1 K78R-mCherry in 143B cells (**g**). FRAP curves are shown on the right (**h**). The data are plotted as the mean ± SD (n = 5 biologically independent experiments). The *p* value was calculated by two-tailed Student’s *t*-test. **i**, HEK293T cells were transiently cotransfected with indicated constructs and analyzed by immunoprecipitation. The experiments in **b**,**i** were repeated three times with similar results. Source data

Next, we evaluated the effect of IRF1 K78ac on PD-L1 expression. The IRF1 K78R mutant failed to upregulate PD-L1 expression and showed reduced abundance at the PD-L1 promoter (Fig. 5h and Extended Data Fig. 9a). Additionally, IRF1 homodimerization was not affected by acetylation at IRF1 K78 (Extended Data Fig. 9b). These results indicate that acetylation at K78 is important for DNA binding of IRF1. Consistent with these data, depletion of KAT8 by sgRNA treatment significantly reduced the abundance of IRF1 at the PD-L1 promoter (Fig. 5i,j). To further support this conclusion, we generated a locus-specific K78R knock-in A375 cell line (hereafter termed K78R cells) using CRISPR–Cas9-mediated homology-directed repair (Extended Data Fig. 9c–e). K78R cells demonstrated a significant reduction in PD-L1 mRNA and protein expression (Fig. 5k and Extended Data Fig. 9f). The abundance of IRF1 at the PD-L1 promoter was also significantly reduced in K78R cells subjected to IFNγ treatment (Fig. 5l). Consistently, intracellular condensates formed by KAT8–IRF1 K78R showed large irregular and less dynamic clusters without colocalization with RNA Pol II-S5P (Fig. 5m,n and Extended Data Fig. 9g,h). Although the K78R mutant showed a similar ability to interact and cocondensate with KAT8 in vitro (Fig. 5o,p and Extended Data Fig. 9i), droplets formed by KAT8–IRF1 K78R showed significantly reduced recruitment of the DNA probes derived from the PD-L1 promoter compared to the WT droplets (Fig. 5o). Taken together, these results indicated that KAT8 acetylates IRF1 at K78, which enhances the DNA binding activity of IRF1 and its localization to the PD-L1 promoter and subsequent activation of PD-L1 mRNA transcription.

### IRF1 acetylation recruits KAT8 to the PD-L1 promoter

**Fig. 6:**
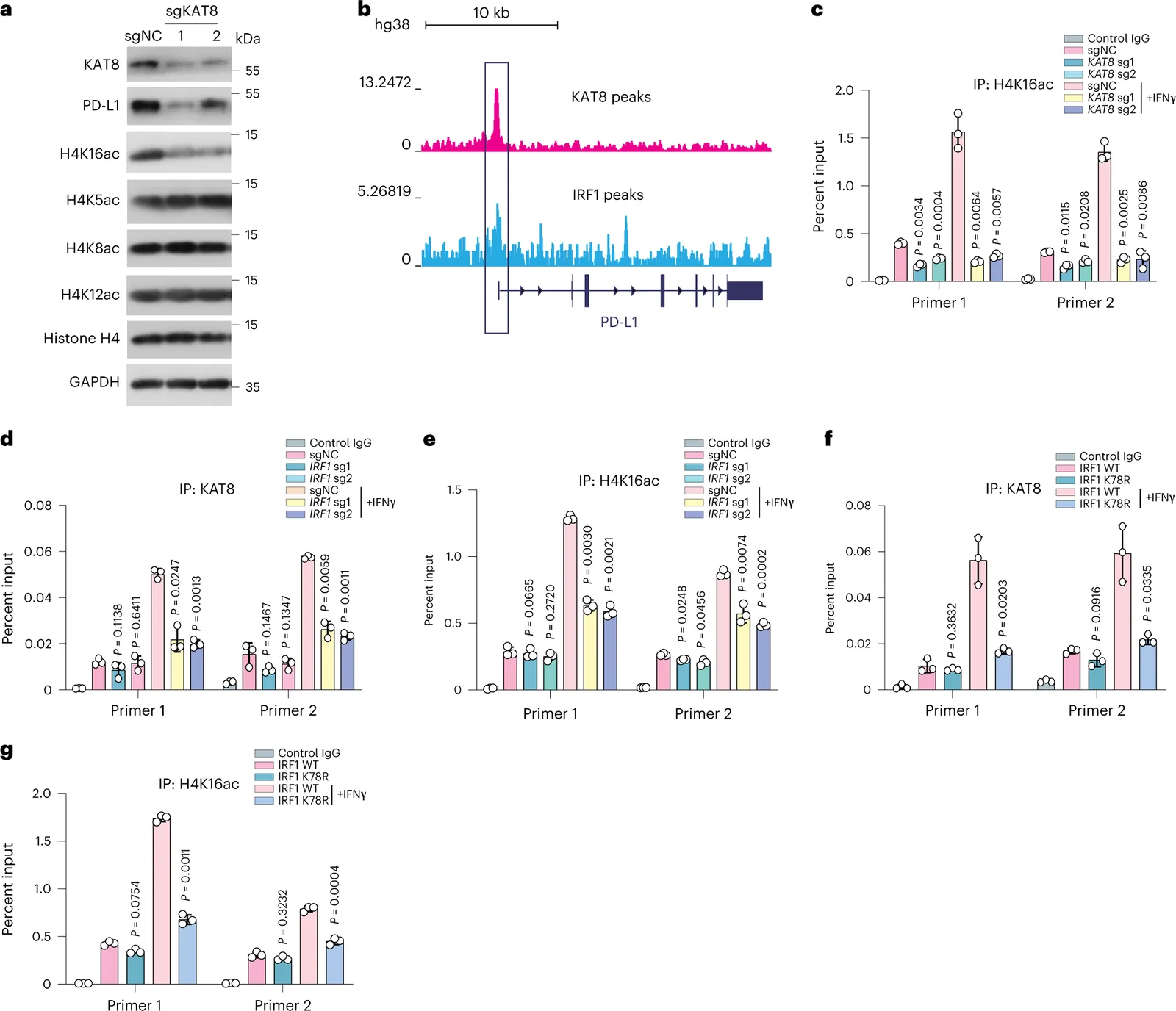
IRF1 acetylation enhances KAT8 recruitment and H4K16 acetylation at the PD-L1 promoter as positive feedback. **a**, Western blotting of H4 acetylation status in A375 cells expressing the indicated sgRNAs. Experiments consisted of three biological replicates with similar results. **b**, ChIP–seq data of the HepG2 cell line from the ENCODE database showing KAT8 and IRF1 peaks enriched at the promoter region of PD-L1; kb, kilobases. **c**, ChIP–qPCR analysis of H4K16 acetylation abundance at the PD-L1 promoter in A375 cells expressing sgNC or sgRNAs targeting *KAT8* with or without 100 U ml^–1^ IFNγ treatment for 12 h. **d**,**e**, ChIP–qPCR analysis of KAT8 (**d**) and H4K16 acetylation (**e**) abundance at the PD-L1 promoter in A375 cells expressing sgNC or sgRNAs targeting *IRF1* with or without 100 U ml^–1^ IFNγ treatment for 12 h. **f**,**g**, ChIP–qPCR analysis of KAT8 (**f**) and H4K16 acetylation (**g**) abundance at the PD-L1 promoter in WT and IRF1 K78R gene-edited A375 cells with or without 100 U ml^–1^ IFNγ treatment for 12 h. Data in **c**–**g** are shown as the mean ± s.d.; *n* = 3 biologically independent experiments. *P* values were calculated by two-tailed Student’s *t-*test. Source data

Given that KAT8 is the main acetyltransferase required for H4K16ac in mammalian cells^22,23^, we investigated whether KAT8 regulates H4K16ac in the PD-L1 promoter. Cells expressing sgRNAs targeting *KAT8* showed an overall reduction in H4K16ac, while other H4 acetylation sites (H4K5ac, H4K8ac and H4K12ac) remained unaffected (Fig. 6a). Chromatin immunoprecipitation with sequencing (ChIP–seq) data from the ENCODE database revealed that KAT8 and IRF1 were enriched at the promoter region of PD-L1 (refs. ^44–46^; Fig. 6b). ChIP–quantitative PCR (ChIP–qPCR) showed that H4K16ac at the PD-L1 promoter was also significantly reduced when KAT8 expression was depleted by sgRNAs (Fig. 6c).

In light of the cocondensation of KAT8 and IRF1, we assessed whether IRF1 in turn enhances KAT8 recruitment at the PD-L1 promoter in response to IFNγ. Cells with IRF1 depletion had a significant reduction in the abundance of KAT8 and H4K16ac at the PD-L1 promoter after IFNγ treatment (Fig. 6d,e). Moreover, K78R cells also showed reduced KAT8 localization and H4K16ac at the PD-L1 promoter after IFNγ exposure (Fig. 6f,g), suggesting that IRF1 acetylation enhances KAT8 recruitment and H4K16ac modification at the PD-L1 promoter as positive feedback.

### Disrupting KAT8–IRF1 condensates inhibits PD-L1 expression

**Fig. 7:**
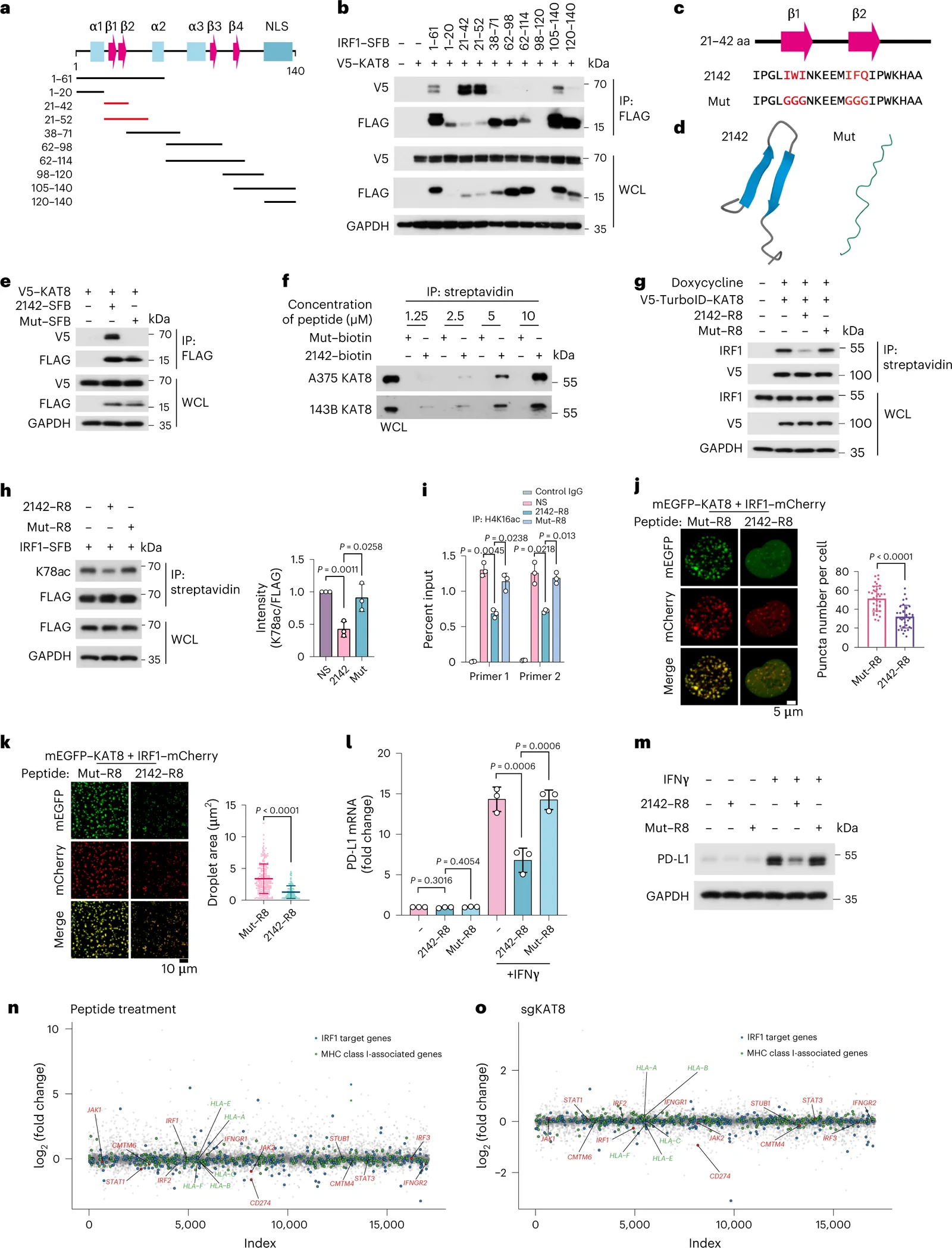
Disrupting KAT8–IRF1 condensates inhibits PD-L1 expression. **a**, Diagram featuring major domains in the IRF1 N terminus (amino acids 1–140) and the truncated constructs that were analyzed in **b**; α, α helix; β, β-sheet. **b**, Interactions between V5–KAT8 and the indicated constructs in HEK293T cells. **c**, The sequence of amino acids 21–42 of IRF1 (2142) and Mut. The core amino acids of the β-sheet predicted by BETApro are in red; aa, amino acids. **d**, AlphaFold prediction of the structures of the 2142 and Mut fragments. **e**, Interactions between V5–KAT8 and 2142–SFB/Mut–SFB constructs in HEK293T cells. **f**, Biotin-conjugated 2142 and Mut peptides at the indicated concentrations were immunoprecipitated from A375 or 143B cell lysates. **g**, Proximity labeling assay of V5-TurboID–KAT8 and endogenous IRF1 in cells with the indicated peptide treatments (Methods). **h**, HEK293T cells transfected with IRF1–SFB were treated with 10 μM peptides, and K78ac levels were analyzed; NS, normal saline. **i**, ChIP–qPCR analysis of H4K16ac abundance at the PD-L1 promoter in A375 cells treated with peptides for 24 h and 100 U ml^–1^ IFNγ for 12 h. **j**, Puncta formed by mEGFP–KAT8 and IRF1–mCherry in 143B cells treated with 10 μM peptide for 12 h; *n* = 36 cells in three independent experiments. **k**, Droplets formed by 50 μM recombinant mEGFP–KAT8 and IRF1–mCherry mixture in the presence of 50 μM 2142–R8 or Mut–R8 peptide in 100 mM NaCl and no crowding agent. Quantification of the droplet area in the shown images is plotted on the right; *n* = 208 droplets for Mut–R8; *n* = 207 droplets for 2142–R8. **l**,**m**, RT–qPCR (**l**) and western blotting (**m**) analysis of PD-L1 expression in A375 cells treated with 10 μM of the indicated peptides. **n**,**o**, RNA-sequencing analysis of A375 cells with peptide treatment (2142–R8/Mut–R8) or KAT8 depletion (sgKAT8/sgNC). Cells were exposed to 100 U ml^–1^ IFNγ for 6 h before collection; *n* = 2 biologically independent replicates for each treatment. The experiments in **b**, **e**–**h**, **j**, **k** and **m** were repeated three times with similar results. Data in **h**–**l** are shown as the mean ± s.d.; *n* = 3 biologically independent experiments. *P* values were calculated by two-tailed Student’s *t*-test. Source data

Considering the role of KAT8–IRF1 condensates in regulating PD-L1 transcription, we reasoned that disrupting the phase separation of KAT8–IRF1 could inhibit PD-L1 mRNA transcription. Deletion of the IRF1 DBD resulted in impaired condensate formation (Fig. 3c,d), while fusing the IDRs of the two proteins with Cry2 (blue light-inducible interaction; Fig. 3g,h) or FKBP12–FRB (rapamycin-inducible interaction; Fig. 4c,d) enhanced condensate formation of the IDRs. These results highlighted the vital role of the structured domain interaction between IRF1 DBD and KAT8 in condensate formation, suggesting that instead of targeting the unstructured IDRs, which is much more challenging, targeting the structured domain interaction might also disrupt KAT8–IRF1 condensates. To test this hypothesis, we constructed a series of IRF1 DBD truncations (Fig. 7a) and cotransfected each with KAT8 in cells to identify the minimal region of IRF1 responsible for interaction with KAT8. The fully conserved N-terminal region (amino acids 21–42, hereafter 2142) in humans and mice, which contains a two-stranded β-sheet structure^36^, was then identified as the main region responsible for the IRF1–KAT8 interaction (Fig. 7b–d), and mutation of the predicted key residues of β-sheets^47,48^ (Mut) abolished the interaction (Fig. 7c–f).

**Extended Data Fig. 10:**
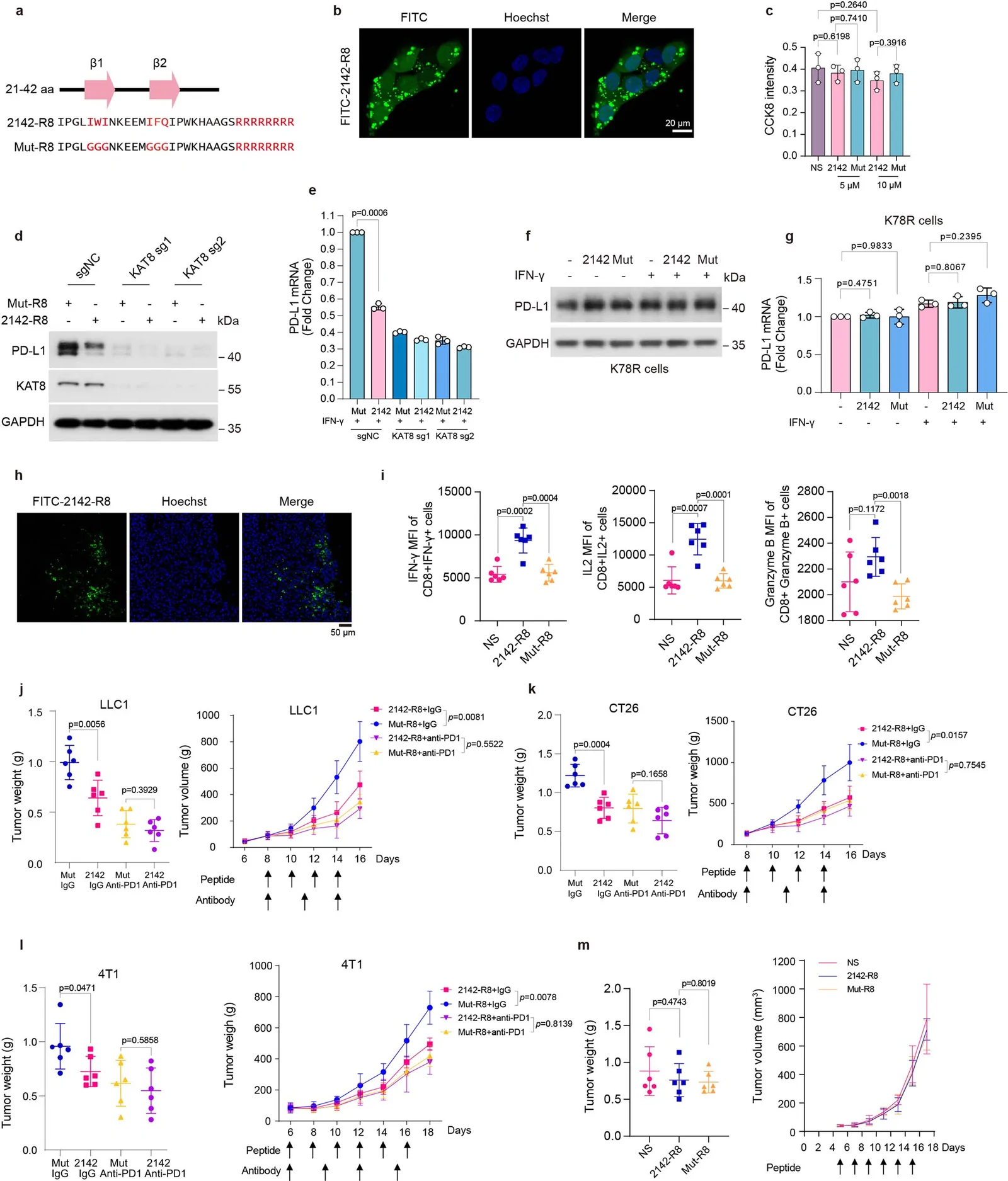
Disruption of KAT8-IRF1 condensates by 2142-R8 enhances antitumor immunity. **a**, Schematics of the amino acid sequences of the peptides. **b**, Representative confocal images showing the infiltration of the FITC-2142-R8 in 143B cells. The experiments were repeated three times with similar results. **c**, CCK8 assay for the peptide cytotoxicity in A375 cells. n = 3 biologically independent experiments. Data are shown as the mean ± SD. *p* values were calculated by two-tailed Student’s *t*-test. **d**,**e**, Western blot (**d**) and qRT-PCR (**e**) analysis of PD-L1 expression in A375 cells expressing indicated sgRNAs treated with 10 μM peptides for 24 h and 100 U/mL IFN-γ for 6 h before harvest. Data are shown as the mean ± SD, n = 3 biologically independent experiments. *p* values were calculated by two-tailed Student’s *t*-test. **f**,**g**, Western blot (**f**) and qRT-PCR (**g**) analysis of PD-L1 in gene-edited IRF1 K78R cells after 10 μM peptides treatment with or without IFN-γ stimulation for 6 h. Data are shown as the mean ± SD, n = 3 biologically independent experiments. *p* values were calculated by two-tailed Student’s *t*-test. **h**, Representative confocal images of cryostat sections showing the infiltration of the FITC-2142-R8 peptide in LLC1 tumor tissues in immune-competent mice. The experiments were repeated twice with similar results. **i**, The Mean Fluorescence Intensity (MFI) of the IFN-γ + , IL2 + cells and Granzyme B + cells in the tumor infiltrated CD8 + T cells. Data are shown as the mean ± SD, n = 6 mice per group. *p* values were calculated by two-tailed Student’s *t*-test. **j**-**l**, Mice bearing LLC1 (**j**), CT26 (**k**), 4T1 (**l**) tumors were treated with 5 mg/kg peptide and 100 μg antibody as indicated. The endpoint tumor weights and tumor volumes were plotted. Data are shown as the mean ± SD, n = 6 mice per group, *p* values of tumor volumes were calculated by Two-way ANOVA with Tukey’s multiple comparisons test. *p* values of tumor weights were calculated by two-tailed Student’s *t*-test. **m**, NOG mice bearing LLC1 tumors were treated with normal saline (NS) or 5 mg/kg peptides; Tumor weights and tumor volumes were measured. n = 6 mice per group. Data are shown as the mean ± SD, *p* values were calculated by two-tailed Student’s *t*-test. Source data

Next, we synthesized two peptides, 2142–R8 and Mut–R8, derived from 2142 and Mut with eight arginine residues fused to their C termini (R8), which enhance cell membrane penetration and nuclear localization (Extended Data Fig. 10a). The peptides could enter the cell nuclei and had low cytotoxicity (Extended Data Fig. 10b,c). To assess the disrupting ability of 2142–R8 for KAT8–IRF1 condensates under physiological conditions in cells, the proximity labeling system with V5-TurboID-tagged KAT8 was used (Fig. 2a). After cells were treated with 2142–R8, the levels of biotin-labeled IRF1 were significantly reduced (Fig. 7g), indicating that the interaction between IRF1 and KAT8 in cells was inhibited. Consequently, the acetylation of IRF1 K78 was inhibited, and H4K16ac at the PD-L1 promoter was reduced (Fig. 7h,i). Consistently, KAT8–IRF1 condensates were reduced after 2142–R8 treatment in cells and in vitro (Fig. 7j,k). Most importantly, 2142–R8 effectively suppressed upregulation of PD-L1 expression (mRNA and protein) in cells treated with IFNγ (Fig. 7l,m) but failed to further reduce PD-L1 mRNA and protein levels in cells expressing sgRNAs targeting *KAT8* or in gene-edited K78R cells (Extended Data Fig. 10d–g), indicating that inhibition of PD-L1 by 2142–R8 depends on the KAT8–IRF1 interaction. Additionally, RNA-sequencing analysis showed that 2142–R8 treatment downregulated PD-L1, while the expression of major histocompatibility complex class I (MHC class I)-related genes and most of the IRF1 downstream remained unchanged, consistent with the data from KAT8-depleted cells (Fig. 7n,o and Supplementary Tables 6 and 7).

### 2142–R8 peptide enhances antitumor immunity

**Fig. 8:**
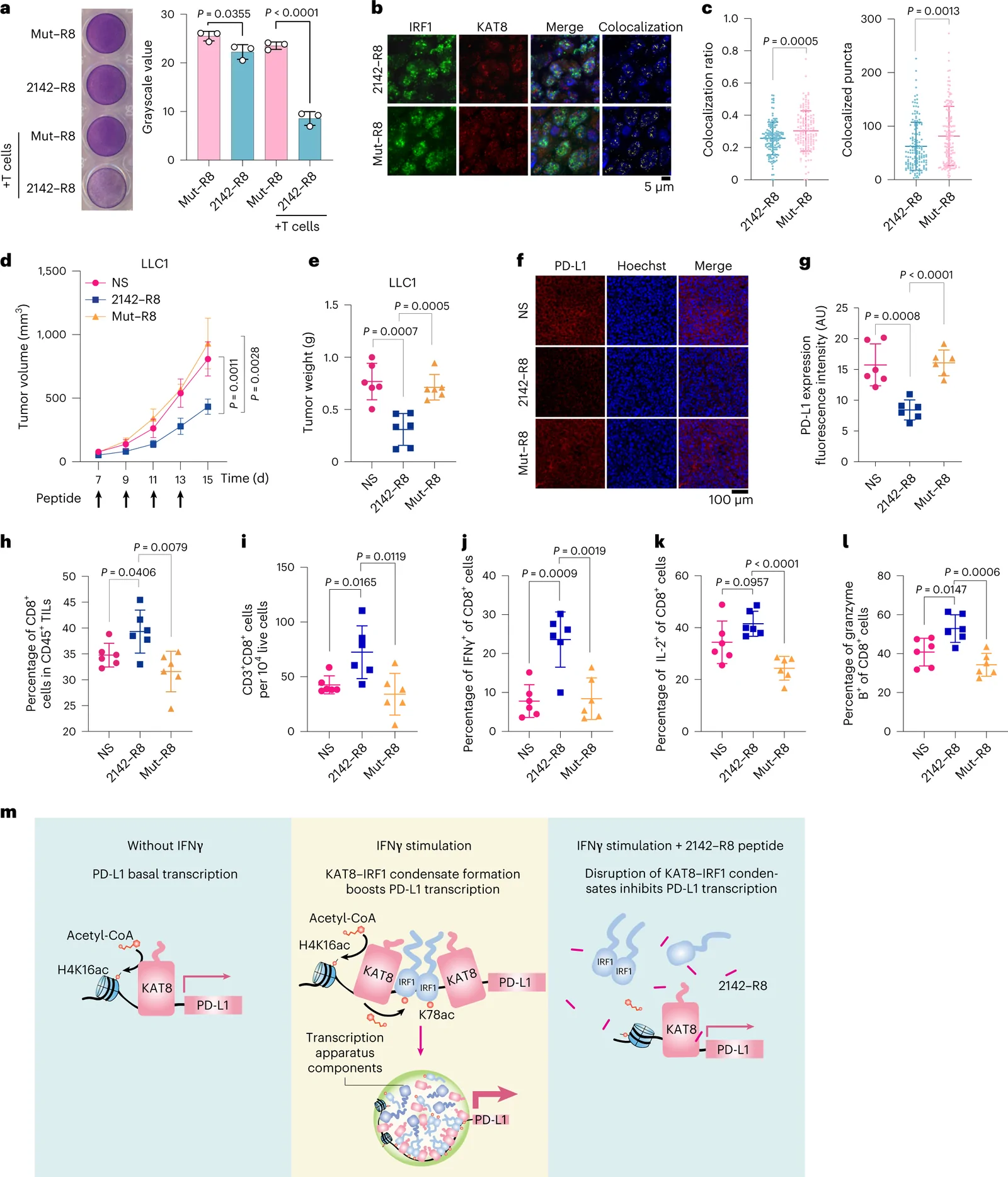
2142–R8 peptide enhances antitumor immunity. **a**, Cytotoxicity assay of A375 cells treated with 10 μM of the indicated peptides for 24 h; *n* = 3 biologically independent experiments. **b**,**c**, Immunostaining for KAT8 and IRF1 in peptide-treated mouse tumor tissues (**b**). Colocalization ratios and colocalized puncta numbers for each cell are plotted in **c**. The colocalization ratio was calculated as the area of colocalized puncta divided by the area of IRF1 puncta; *n* = 150 cells for 2142–R8; *n* = 140 cells for Mut–R8. **d**–**l**, Mice bearing LLC1 tumors were randomly divided into three indicated groups (*n* = 6 mice per group). Peptides (5 mg per kg (body weight)) were administered via intraperitoneal injection at the indicated time points. Tumor size (**d**) and weights (**e**) were measured. The immunofluorescence intensity of PD-L1 expression in each group was analyzed (**f** and **g**). The ratios of CD3^+^CD8^+^ T cells in CD45^+^ tumor-infiltrating lymphocytes (**h**) and total live cells (**i**) were analyzed. The percentages of IFNγ^+^ (**j**), IL-2^+^ (**k**) or granzyme B^+^ (**l**) cells in the tumor-infiltrating CD8^+^ T cells were plotted; TILs, tumor-infiltrating lymphocytes. Data in **c**, **d**, **e** and **g**–**l** are shown as the mean ± s.d.; *P* values in **d** were calculated by two-way ANOVA with Tukey’s multiple-comparison test, and *P* values in **c**, **e** and **g**–**l** were calculated by two-tailed Student’s *t*-test; AU, arbitrary units. **m**, Schematic illustration showing that disruption of KAT8–IRF1 condensates diminishes PD-L1 expression. Left, without IFNγ stimulation, KAT8 localizes to the promoter region of PD-L1 and acetylates H4K16. Middle, with IFNγ stimulation, in addition to H4K16ac, KAT8 also acetylates IRF1 K78 to promote its binding on the PD-L1 promoter. The condensates formed by KAT8 and IRF1, probably facilitated by other transcription factors, cofactors and mediators, could further recruit the transcription apparatus to boost PD-L1 transcription. Right, the 2142–R8 peptide disrupts KAT8–IRF1 condensate formation, inhibits IRF1 K78ac and DNA binding and subsequently inhibits PD-L1 transcription even in the presence of IFNγ. Source data

We then assessed the efficacy of 2142–R8 in enhancing antitumor immunity. An in vitro cytotoxicity assay showed that 2142–R8, but not Mut–R8, enhanced T cell killing (Fig. 8a). In vivo, intraperitoneal injection of the peptides could infiltrate tumor tissues (Extended Data Fig. 10h). Treatment with 2142–R8, but not Mut–R8, reduced the colocalization of KAT8–IRF1 puncta, tumor volumes and tumor weights in mice bearing LLC1 tumors (Fig. 8b–e). Likewise, tumor PD-L1 expression was reduced, and the infiltration of active CD8^+^ T cells was increased by 2142–R8 but not Mut–R8 (Fig. 8f–l). Treatment with 2142–R8 also enhanced activation of the infiltrated CD8^+^ T cells in mouse tumors (Extended Data Fig. 10i). The tumor-inhibitory effects of 2142–R8 were also observed in CT26 and 4T1 tumor models (Extended Data Fig. 10j–l). By contrast, 2142–R8 failed to further enhance tumor reduction when blocking the PD-1/PD-L1 axis by anti-PD-1 treatment or in NOG mice (Extended Data Fig. 10j–m). Taken together, these results illustrate that the 2142–R8 peptide inhibits PD-L1 expression and enhances antitumor immunity in vivo.

## Discussion

Understanding the mechanism of PD-L1 regulation is critical to develop more strategies for cancer immunotherapy, as targeting the PD-1/PD-L1 axis has been proven effective and has become a first-line treatment in multiple cancers^49^. PD-L1 is often upregulated in tumors by intrinsic oncogenic signaling and extrinsic stimulations^50^. IFNγ, which is predominantly secreted by T cells, is a key regulator in the tumor immune response and exerts complex effects on the tumor microenvironment^51,52^. PD-L1 upregulates the expression of MHC/HLA molecules on tumor cells, which enhances recognition and killing by cytotoxic T cells^52^. However, at the same time, IFNγ potently induces upregulation of PD-L1 in tumor cells, which induces exhaustion and eventual apoptosis of tumor-infiltrating T cells^52^. In fact, preclinical studies and clinical trials on IFNγ treatment show discrepancies in its effectiveness against tumors^53^. IRF1, one of the transcription factors downstream of IFNγ and induced by STAT1, is the key transcription factor required to activate PD-L1 expression^19,54^, while the IFNγ signaling pathway does not always require IRF1 (refs. ^19,55^). Ectopic expression of IRF1 is sufficient to induce PD-L1 upregulation even in the absence of IFNγ^19^. Cells with IRF1 depletion show impaired PD-L1 upregulation and are more susceptible to T cell-mediated killing^56^. In this report, as illustrated in Fig. 8m, we demonstrated that KAT8 could cocondensate with IRF1, which promotes IRF1 K78ac and enhances PD-L1 promoter binding and subsequently activates transcription, highlighting the critical role of KAT8–IRF1 condensates in shaping the tumor microenvironment by regulating PD-L1 transcription.

Although KAT8 is a well-established histone acetyltransferase harboring histone and non-histone proteins as substrates^22–27^, previous studies focused on the specific structured enzyme–substrate interactions and whether the predicted IDR of KAT8 is functional remains unknown. In this study, we uncovered an interesting KAT8–IRF1 interaction pattern that forms transcriptional condensates. The formation of KAT8–IRF1 condensates depends on both structured domain and promiscuous IDR interactions. We further uncoupled specific KAT8–IRF1 interactions using a rapamycin-inducible system and demonstrated that KAT8–IRF1 IDR-mediated condensation contributes to transactivation. After IFNγ stimulation, KAT8–IRF1 condensate formation may be facilitated by interactions with other transcriptional factors, cofactors and mediators, collaboratively boosting PD-L1 mRNA transcription. These findings support a notion that epigenetic modifiers and transcription factors may act as biomolecular condensates to synergistically amplify the transactivation effect and thus respond rapidly to environmental stimulation.

There is growing evidence of biomolecular condensates in the regulation of cancer development^57–60^, suggesting that targeting this process may be promising. In the context of KAT8–IRF1 condensates, we demonstrated that the specific structured interaction between the IRF1 DBD and KAT8 is a prerequisite for condensate initiation, while the weak promiscuous interactions of the IRF1 and KAT8 IDRs promote condensate formation. Based on this mechanism, we identified the 2142–R8 peptide, which can block IRF1 DBD and KAT8 interaction and disrupt the formation of KAT8–IRF1 condensates, subsequently suppressing PD-L1 expression and enhancing antitumor immunity in vitro and in vivo. Overall, our data show that inhibiting cancer-related condensate formation might be a potential strategy for cancer therapy.

## Methods

### Ethics statement

The Ethics Committee of Sun Yat-sen University Cancer Center and Animal Research Committee of Sun Yat-sen University Cancer Center approved this study (GZKJ2020-019 and L102012018110H). All experiments performed in this study conform to related ethical guidelines. The maximal tumor size of animal experiments was 2,000 mm^3^, and all experiments did not exceed this limit. The paraffin-embedded cancer tissue samples mentioned were obtained from the Department of Pathology at Sun Yat-sen University Cancer Center. Human peripheral blood mononuclear cells (PBMCs) were obtained from healthy donors. All blood donors in this study signed informed consent. The human multiple organ cancer tissue arrays were purchased from Shanghai Outdo Biotech.

### Cell lines

Cell lines 143B, HCT116 and U2OS were obtained from ATCC. HEK293T, A375, A549, LLC1, OVCAR3, PC9, HCC1937, PC3, T24, DU145, CT26 and 4T1 cell lines were obtained from Cellcook. SNU-1040 was obtained from Biospes. All cell lines included in this study were validated by short-tandem repeat DNA profiling and were consecutively passaged less than 10 times. Cell culture was performed following instructions from ATCC.

### Plasmid construction

For mammalian expression plasmids, *KAT8* and *IRF1* sequences were amplified and assembled into the expression vectors with tags (HA, 3×MYC, FLAG, SFB, V5, mEGFP, TagBFP or mCherry). Plasmids containing CBP, P300, GCN5, PCAF and Tip60 were obtained as described previously^61^. KAT8 C316S and IRF1 K78R constructs were generated from WT KAT8 and IRF1 with a one-step cloning kit (Vazyme, C113-02), and the sequences were verified by Sanger sequencing. MED1 IDR includes amino acids 948–1574 of MED1, MYC IDR includes amino acids 210–360 of MYC, and OCT4 IDR includes amino acids 1–138 of OCT4. Prokaryotic expression plasmids of 6×His-mCherry, 6×His-mEGFP, 6×His-KAT8, 6×His-mEGFP–KAT8, 6×His-mEGFP–KAT8 C316S, 6×His-IRF1, 6×His-IRF1–mCherry and 6×His-IRF1 K78R–mCherry were cloned into a modified pGEX-4T vector with glutathione *S*-transferase sequences deleted.

### Animal experiments

Six- to 8-week-old C57BL/6N, BALB/c and NOG female mice were purchased from Beijing Vital River Laboratory Animal Technology. Four to 6 d after subcutaneous injection of LLC1 cells (1 × 10^6^) expressing non-targeted control sgRNA (sgNC) or *KAT8* sgRNA at the right inguinal area of C57BL/6N mice (*n* = 6 per group), tumor lengths and widths were measured every 2 d using calipers. Tumor volumes were calculated as length × width^2^/2 (mm^3^). For anti-PD-1 treatment groups, sgNC- and sgKAT8-expressing LLC1 cells were randomly divided into two subgroups (*n* = 6 per group), one for anti-PD-1 (BioXcell, BE0146) and one for IgG (BioXcell, BE0089) treatment. Antibodies were dissolved in InVivoPure (pH 7.0) dilution buffer (BioXcell, IP0070) and intraperitoneally injected into mice at a dose of 100 μg at the indicated times. For peptide treatment assays, mice were randomly assigned into three treatment groups: normal saline, 2142–R8 and Mut–R8 (*n* = 6 per group). Mice from the 2142–R8 or Mut–R8 groups were intraperitoneally injected with 5 mg per kg (body weight) peptide in normal saline every other day. Mice from the normal saline group were intraperitoneally injected with normal saline every other day. For peptide and antibody combination therapies, LLC1 (1 × 10^6^), CT26 (1 × 10^6^) and 4T1 (3 × 10^5^) cells were injected as mentioned above. Mice were treated with peptide (5 mg per kg (body weight) peptide) and/or antibody (100 μg for each mouse) as indicated. The experimental procedure used for peptide treatment of immunodeficient mice was similar to that used for immune-competent mice.

### ChIP assays

ChIP assays were performed using the EZ-Magna ChIP A/G chromatin immunoprecipitation kit (Millipore, 17-10086) following the manufacturer’s instructions. Briefly, 1 × 10^7^ cells were cross-linked with 1% formaldehyde. After lysis, cell nuclei were subjected to sonication with a Covaris E220 focused ultrasonicator (150 peak incident power, 7.5% duty factor, 200 cycles, 1 min) to shear DNA. For immunoprecipitation, 100 µl of sheared chromatin was diluted to a total volume of 1 ml. Antibodies to H4K16ac, KAT8 and IRF1 as well as normal IgG were added and incubated overnight at 4 °C. Protein A/G beads were added to the sheared chromatin and incubated for another 2 h at 4 °C. After washing, the DNA was purified using a Tiangen Universal DNA purification kit (DP214-03) according to the manufacturer’s instructions. The purified DNA was used for qPCR analysis. ChIP–qPCR primer sequences are provided in Supplementary Table 8.

### FRAP

FRAP assays were performed using a Zeiss LSM880 confocal microscope. Droplets were formed with 50 μM recombinant mEGFP–KAT8 and IRF1–mCherry in the presence of 500 mM NaCl and 4% PEG 8000. To bleach the corresponding fluorescence signal, 100% of the maximum laser power of the 488-nm laser and 30% of the maximum laser power of the 561-nm laser with 300 interations were used. The bleached area was approximately 2 μm in diameter, and 60 rounds of imaging were performed after bleaching with the indicated intervals. Relative recovery was normalized to the initial intensity for each condensate, and the means and standard deviation of the recovery time were calculated.

### In vitro acetylation assay

Purified SFB–KAT8, IRF1–SFB WT and IRF1–SFB K78R mutant were incubated with acetyl-coenzyme A (acetyl-CoA) in HAT buffer (Millipore, 17-329) at 30 °C for 0.5 h. Acetylation of IRF1 was analyzed by SDS–PAGE.

### Acetylation in droplets and bulk samples

For the total acetylation samples, purified mEGFP–KAT8 and IRF1–mCherry at the indicated concentrations were incubated in reaction buffer (25 mM Tris-HCl, 150 mM NaCl, 10% PEG 8000, 10% glycerol, 1 mM DTT and 20 μM acetyl-CoA, pH 7.4) at 30 °C for 1 h. For acetylation in droplets and in bulk samples, reaction products with 5 μM mEGFP–KAT8 and IRF1–mCherry were centrifuged for 30 min at 21,000*g* at 25 °C, and the droplets and bulk samples were transferred to independent tubes. The reactions were terminated by the addition of 5× SDS–PAGE loading buffer, boiled at 99 °C for 10 min and quantified by western blotting.

### IRF1 K78ac antibody production

Two synthetic IRF1 acetylation peptides (DPKTW-(acetyl)K-ANFRC and CPDPKTW-(acetyl)K-ANFRSA) were conjugated with carrier protein KLH for immunization of rabbits; the unmodified IRF1 K78 peptide (DPKTWKANFRC) was used for depletion of non-K78ac-reacting antibodies. This antibody was customized at PTM Biolabs and validated by enzyme-linked immunosorbent assay and western blotting.

### Cell immunofluorescence

Immunofluorescence was performed as described previously^62^. Briefly, cells were washed with PBS twice before fixation in 4% paraformaldehyde for 15 min. After permeabilization with PBS containing 0.5% Triton X-100 for 10 min and blocking with serum for 30 min, cells were incubated with primary antibodies for 1 h and washed with 0.05% Triton X-100 in PBS for 5 min four times and in PBS for 5 min once. Afterward, cells were incubated with secondary antibodies for 45 min. Cell nuclei were stained for 3 min with Hoechst 33342 (Thermo Fisher, H3570). After washing, cells were mounted (Beyotime, P0128M) and analyzed.

### Tissue IHC and fluorescence IHC

For IHC analysis of the human multiple organ cancer tissue arrays, slices were stained with anti-PD-L1 (GeneTex, GTX104763) or anti-KAT8 (Abcam, ab200660), followed treatment with anti-rabbit IgG secondary antibody and DAB reagents. Each case on the array was scored by a pathologist who was blinded to the study. For the correlation analysis of PD-L1 and KAT8 expression in cancer tissues, the median IHC scores of PD-L1 and KAT8 were used to assign the cases to the high and low expression groups, and a chi-square test was used to determine the *P* value.

For fluorescence IHC staining of PD-L1 expression on mouse tumor tissues with peptide treatment, fresh tumor tissues were fixed with 10% formalin. After paraffin embedding, tissue blocks were sliced at a thickness of ~4 μm. Slices were subjected to deparaffinization, antigen retrieval and blocking and were stained with anti-PD-L1 (Cell Signaling Technology, 64988) for 2 h at room temperature, followed by treatment with Alexa Fluor 594-conjugated secondary antibody (Invitrogen, A32754) for 45 min at room temperature. Cell nuclei were stained for 3 min with Hoechst 33342.

For fluorescence IHC costaining of PD-L1 and CD8 or KAT8 and IRF1 on mouse tumor tissues slices, experiments were performed following the instructions from the Panovue TSA kit (Panovue, 10079100020). Briefly, slices were incubated with anti-CD8α (Cell Signaling Technology, 98941) or anti-IRF1 (Cell Signaling Technology, 8478) for 2 h, incubated with anti-rabbit IgG secondary antibody for 10 min, washed and treated with PPD520-conjugated TSA reagent for 10 min. Slices were then washed and subjected to antigen retrieval and blocking again, and slices were stained with anti-PD-L1 (Cell Signaling Technology, 64988) or anti-KAT8 (Abcam, ab200660) for 2 h at room temperature. Slices stained for PD-L1 were incubated with anti-rabbit IgG secondary antibody for 10 min, washed and treated with PPD650-conjugated TSA reagent for 10 min. Slices with KAT8 staining were incubated with Alexa Fluor 594-conjugated secondary antibody (Invitrogen, A32754) for 45 min. After washing, slices were stained with DAPI for 15 min.

For fluorescence IHC analysis of samples from individuals with cancer, slices were stained with either anti-KAT8 (Abcam, ab200660; 1:400) and anti-PD-L1 (Cell Signaling Technology, 29122) or anti-IRF1 (Cell Signaling Technology, 8478) and anti-PD-L1 (Cell Signaling Technology, 29122) for 1 h at room temperature. Afterward, the slices were incubated with secondary antibodies for 45 min followed by Hoechst 33342 for 3 min. The mounted slices were analyzed by confocal microscopy and Fiji software.

### In vitro cytotoxicity assay

Human PBMCs were isolated from the buffy coat of whole blood by gradient centrifugation using Ficoll 400. PBMCs were then stimulated for 48 h in 12-well plates (approximately 5 × 10^6^ cells per well) coated with T cell activators (anti-CD3 and anti-CD28, STEMCELL Technologies, 10971) in RPMI 1640 medium supplemented with 10% fetal bovine serum (FBS). Activated T cells were counted and seeded into 48-well plates with tumor cells (1 × 10^5^ cells per well for both cell types) in RPMI 1640 medium. For peptide treatment, 10 μM 2142–R8 or Mut–R8 was added into the cell medium and incubated for 24 h. T cells were carefully washed away with PBS, and the adherent tumor cells were fixed with 4% paraformaldehyde for 15 min before they were stained with crystal violet for 30 min.

### IRF1 K78R knock-in A375 cells

The knock-in experiment was performed as previously described^63^. An efficient *IRF1*-targeting sgRNA was selected, and a DNA donor template containing mutations was designed. The donor template was cloned into the pSIN vector with EF1α promoter deletion. To increase the efficiency of positive clone selection, a fragment encoding EGFP was inserted into the donor template in the intron sequence between exon 3 and exon 4. Additionally, an internal ribosome entry site was placed upstream of *EGFP*. CRISPR–Cas9 and the generated donor constructs were then cotransfected into A375 cells. Six days after transfection, flow cytometry sorting was performed to obtain EGFP^+^ cells, and single clones were picked. Site-specific PCR and Sanger sequencing were used to validate the gene-edited clone. The sequences of K78R knock-in donor template and sgRNA are provided in Supplementary Table 8.

### Peptide pulldown assay

The 2142–biotin and Mut–biotin peptides with ≥98% purity were synthesized and purchased from Genscript Biotech. One milligram of cell lysate from A375 or 143B cells was incubated with peptides at the indicated concentrations for 2 h at 4 °C, and precleared streptavidin sepharose (GE, 17511301) was added to the lysates with rotation for another 2 h. The coimmunoprecipitated KAT8 was analyzed by western blotting.

### Peptide penetration assays

FITC–Ahx-2142–R8 peptides with ≥98% purity were synthesized and purchased from Genscript Biotech. For cell penetration, 143B cells were treated with 100 nM FITC–Ahx-2142–R8 peptide for 6 h and fixed. For tumor tissue infiltration analysis, mice bearing LLC1 tumors were intraperitoneally injected with 5 mg per kg (body weight) FITC–Ahx-2142–R8 peptides. Twenty-four hours later, the tumors were isolated and frozen immediately. Cryostat sections (4-μm thickness) were fixed with 4 °C acetone for 10 min. Hoechst 33342 was used to stain nuclei. Images were captured using a Zeiss LSM880 microscope.

### Protein expression and purification

Protein purification was performed as described previously^64^. Prokaryotic expression plasmids containing 6×His-mEGFP, 6×His-mCherry, 6×His-KAT8, 6×His-mEGFP–KAT8, 6×His-mEGFP–KAT8 C316S, 6×His-IRF1, 6×His-IRF1–mCherry and 6×His-IRF1 K78R–mCherry were expressed in *Escherichia coli* BL21(DE3) cells at 18 °C overnight with 0.5 mM isopropyl-β-d-thiogalactoside (Biofroxx, 1122GR100). Bacteria were suspended in Tris buffer (50 mM Tris-HCl, 500 mM NaCl and 1 mM DTT, pH 7.4) and lysed in an ultrahigh-pressure homogenizer. The cleared supernatant was obtained after lysates were centrifuged at 12,000*g* for 30 min at 4 °C. BeyoGold His-tag purification resin (Beyotime, P2218) was preequilibrated and used to purify the His-tagged recombinant proteins with rotation at 4 °C for 2 h. The resin was centrifuged at 1,000*g* for 5 min at 4 °C and washed with 10 volumes of lysis buffer four times. Proteins were eluted with elution buffer (50 mM Tris-HCl, 500 mM NaCl, 1 mM DTT and 500 mM imidazole, pH 7.4) and dialyzed in ~300 times the volume of the samples in Tris buffer (50 mM Tris-HCl, 500 mM NaCl and 1 mM DTT, pH 7.4) at 4 °C overnight. Finally, the dialyzed proteins were concentrated and purified by size-exclusion chromatography using a Superdex 200 increase 10/300 GL column (GE Healthcare). For purification of SFB–KAT8, IRF1–SFB and IRF1 K78R–SFB, HEK293T cells were used for transfection. SFB-tagged proteins in cell lysates were enriched using streptavidin sepharose (GE, 17511301). The proteins were then eluted with 2 mg ml^–1^ biotin. The size and purity of the eluted proteins were analyzed by SDS–PAGE and Coomassie blue staining. The protein concentrations were measured by UV absorbance at 280 nm, and the extinction coefficients were calculated using the ProtParam tool^65^.

### Phase separation assay

Protein purifications were performed as described previously^64^. In vitro-purified proteins were incubated in phase separation buffer (25 mM Tris-HCl and 0.5 mM DTT, pH 7.4) at the indicated NaCl concentrations in the presence or absence of PEG 8000, as described in the figure legends. After a 5-min incubation, the protein mixture was transferred into a 384-well plate (Cellvis, p384-1.5H-N) and analyzed by confocal microscopy. Quantification of the droplets was determined using ImageJ, and the following batch analysis parameters were used: image type, 8 bit; adjust, auto threshold; method = RenyiEntropy white; analyze particles, size = 0-Infinity; circularity = 0.00–1.00. For turbidity assays, mEGFP–KAT8 and IRF1–mCherry proteins (0.075–10 μM) were mixed in phase separation buffer at 150 mM NaCl and 10% PEG 8000 for 5 min. The mixtures were measured at an optical density at 600 nm using an MD SpectraMax Plus 384 microplate reader (Molecular Devices).

### OptoDroplets assay

The optoDroplets assay was performed as described previously^66^. IRF1^115–325^–mCherry–Cry2 and KAT8^1–68^–mEGFP–Cry2 were transfected into HEK293T cells separately or together. Cells were illuminated with a 488-nm laser every 10 s. Images were captured for mCherry, mEGFP or both signals every 10 s using a Nikon CSU-W1 spinning disk confocal microscope.

### Gal4-UAS–mEGFP reporter assay

For the Gal4-UAS–mEGFP reporter cells, a reporter vector containing nine Gal4 UAS upstream of the mEGFP and flanked SB IRDR-L/R elements were cotransfected with SB100 (Addgene, 34879) into 143B cells via Lipofectamine 3000. Twenty-four hours after transfection, 2 μg ml^–1^ puromycin was used to select the integrated cells. One week after selection, fluorescence-activated cell sorting (FACS) was used to isolate a subset of uniform mEGFP^+^ cells and culture for further investigation. The Gal4 DBD was assembled with IRF1^115–325^ and FRB, and KAT8^1–68^ was assembled with FKBP12 into pSIN vector using a one-step cloning kit (Vazyme, C113-02). The sequences were verified by Sanger sequencing. These two vectors were transfected into Gal4-UAS–mEGFP reporter cells separately or together and with a pSIN-mCherry vector as a quantification control. Medium was changed 6 h later, and DMSO or rapamycin (200 nM) was added into the medium for 18 h. The expression levels of mEGFP and mCherry were quantified by FACS. To further investigate the rapamycin dose-dependent activation on UAS reporter cells, IRF1^115–325^–FRB–Gal4 DBD and KAT8^1–68^–FKBP12 vectors containing SB IRDR-L/R elements and the blasticidin resistance gene were cotransfected with SB100 into the UAS–mEGFP reporter cells. Blasticidin (10 μg ml^–1^) was used for selection for 1 week. Cells were then seeded into 96-well plates via limiting dilution, and moderate mEGFP-expressing single clones were isolated to get the IRF1^115–325^–FRB-Gal4 DBD and KAT8^1–68^–FKBP12 stably integrated UAS–mEGFP reporter cells. Reporter cells were treated with the indicated concentrations of rapamycin for 24 h, and mEGFP signal intensity was quantified by FACS. CytExpert (2.4) was used for analysis of the flow cytometric data.

### Estimation of 143B nuclear volume

Nuclear volume was measured as previously described^67^. Briefly, live 143B cells were stained with Hoechst 33342. Three-dimensional images were captured by confocal microscopy (ZEISS LSM880). For each nucleus, the length (*l*), width (*w*) and depth (*d*) were measured using ZEN software (ZEISS). The nuclear volumes were calculated with the ellipsoid volume formula *V* = (4/3)*π*(*l*/2 × *w*/2 × *d*/2). Thirty randomly selected cells were measured and analyzed.

### CCK8 assay

The CCK8 assay was performed as described previously^68^. Briefly, A375 cells were seeded into 96-well plates with 2,000 cells per well for 24 h and treated with normal saline and the indicated concentrations of 2142–R8 or Mut–R8 for 48 h. Afterward, CCK8 was added into the medium for 1 h. CCK8 signal was detected with a microplate reader at 450 nm.

### Quasar 705 DNA probe production

The probe DNA fragment was amplified from tumor DNA by PCR from the specific region of the PD-L1 promoter using a pair of Quasar 705 fluorophore-labeled PCR primers (RuiBiotech). Amplification of genome DNA templates was performed using KOD FX Neo polymerase (TOYOBO, KFX201). Quasar 705-labeled probes were purified using a gel extraction kit (Tiangen, DP209-2). The wild-type and IRF1 motif mutation PD-L1 promoter DNA sequences are provided in Supplementary Table 8.

### Proximity labeling assay

A proximity labeling assay was performed as described previously^31^. To label KAT8-interacting proteins, doxycycline-inducible V5-TurboID-KAT8-expressing A375 stable cells were treated with 500 ng ml^–1^ doxycycline for 24 h. Cells were then stimulated with 100 U ml^–1^ IFNγ for 6 h, and 15 min before collection, 50 μM biotin was added to the culture medium. Afterward, cells were lysed and sonicated in RIPA–SDS buffer (50 mM Tris-HCl (pH 7.5), 150 mM NaCl, 0.125% SDS, 0.125% sodium deoxycholate and 1% Triton X-100). The clear lysates were incubated with streptavidin beads at 4 °C overnight, and the beads were then washed with buffers in the following order: 1 M KCl buffer, 0.1 M Na_2_CO_3_ buffer, 2 M urea in 10 mM Tris buffer and RIPA–SDS buffer twice. The washed beads were suspended in 5× loading buffer (Beyotime, P0015L) containing 10% SDS and boiled for 5 min, and the boiled proteins were subjected to mass spectrometry analysis. For analysis of the effect of 2142–R8 peptide on blocking the KAT8–IRF1 interaction under physiological conditions, doxycycline-inducible V5-TurboID-KAT8-expressing A375 stable cells were treated with 10 μM 2142–R8 or Mut–R8 peptide and doxycycline (500 ng ml^–1^) for 24 h. The cells were then exposed to 100 U ml^–1^ IFNγ for 6 h, and 50 μM biotin was added to the cell culture medium 15 min before cell collection. Proteins were then prepared as mentioned above and subjected to SDS–PAGE analysis.

### Whole-genome CRISPR–Cas9 gene knockout screens

Whole-genome CRISPR–Cas9 gene knockout screens were performed according to a previous study^69^. Briefly, 143B cells (approximately 60 million cells) were infected with a low multiplicity of infection (~0.3) of the whole-genome CRISPR–Cas9 knockout lentivirus library. After 24 h, cells were passaged and cultured for 7 d or 14 d in medium supplemented with 0.5 μg ml^–1^ puromycin. Twenty-four hours before cell sorting, cells were exposed to 100 U ml^–1^ IFNγ. For cell sorting, cells were collected and suspended in cold PBS before they were stained with anti-PD-L1 (BD, 558017) and sorted. Cells in the total population with the top 5% and tail 5% PD-L1 expression intensity were collected and subjected to DNA extraction, sgRNA amplification and sequencing. The resulting data were analyzed by the model-based analysis of genome-wide CRISPR–Cas9 knockout method (MAGeCK)^70^.

### dCas9-SunTag PD-L1 promoter visualization

The assay for PD-L1 promoter visualization was performed as previously described^38^. In brief, 20 sgRNAs (sgARRAY) around the PD-L1 promoter were cloned into a PUC19 backbone via Golden Gate reaction. The sgARRAY was verified via EcoRI digest. pTETON-dCas9-24*GCN4, pTETon-scFv-GCN4-sfGFP and sgARRAY were cotransfected into 143B cells for 6 h, and 0.5 μg ml^–1^ doxycycline was added into the medium. Eighteen hours later, cells were treated with 100 U ml^–1^ IFNγ for another 6 h. Afterward, cells were prepared for immunofluorescence staining. Primary antibodies were anti-KAT8 (Atlas, HPA066324) and anti-IRF1 (Santa Cruz, sc-74530). Secondary antibodies were anti-rabbit Alexa Fluor 594 (Invitrogen, A32754) and anti-mouse Alexa Fluor 405 (Invitrogen, A-31553). SIM analysis was performed using the N-SIM S superresolution microscope (Nikon). The sequences of the sgARRAY are provided in Supplementary Table 8.

### Mass spectrometry

To identify the acetylated lysine residues of IRF1, HEK293T cells were cotransfected with KAT8 and IRF1–SFB for 24 h and treated with 5 μM TSA and 5 mM nicotinamide for another 24 h. IRF1–SFB protein was then enriched by streptavidin beads. The washed beads were boiled for 5 min in buffer containing 2% SDS to elute bound proteins in preparation for SDS–PAGE analysis. The band corresponding to approximately 55–72 kDa was excised. For analysis of KAT8-interacting proteins, after SDS–PAGE analysis, the whole lane was excised and digested with trypsin at 37 °C overnight to obtain the peptide extract. Peptides were then desalted and lyophilized. After separation on an analytical column, the peptides were analyzed by mass spectrometry.

### Flow cytometry

Flow cytometry was performed as described previously^71^. For analysis of the tumor-infiltrating cytotoxic T cells in mice, resected tumors were first cut into small pieces and incubated in digestion buffer (50 U ml^–1^ DNase I and 0.4 mg ml^–1^ collagenase IV in RPMI 1640 medium) at 37 °C for 1 h with shaking at 100 r.p.m. After passing through a 70-μm cell strainer, suspensions of single cells were washed with PBS three times. BD Horizon fixable viability stain 700 (FVS700; BD, 564997) was used to label non-viable cells. After the 10-min incubation, FVS700 was washed away with staining buffer (2% FBS in PBS). For experiments with sgKAT8 tumors, cells were stained with primary antibodies (anti-CD45 (Biolegend, 103112), anti-CD3ε (Biolegend, 100308) and anti-CD8α (eBioscience, 11-0081-82)) at 4 °C for 30 min. For experiments with peptide treatment, cells were stained with primary antibodies (anti-CD45 (Biolegend, 103134), anti-CD3ε (Biolegend, 100306) and anti-CD8α (Biolegend, 100708)) at 4 °C for 30 min. Stained cells were analyzed by flow cytometry.

For analysis of cell surface PD-L1 expression in human cancer cell lines, flow cytometry was performed using anti-CD274 (Biolegend, 329706).

For intracellular marker analysis, single cells obtained from mouse tumors were suspended in RPMI 1640 medium with 10% FBS at a concentration of about 1 × 10^6^ cells per ml. A leukocyte activation cocktail (BD, 550583) was added into the medium at a concentration of 2 μl for 1 ml of cell suspension. After incubating at 37°C for 4 h, cells were collected, and FVS700 (BD, 564997) was used to label non-viable cells. After washing, cells were incubated with anti-CD8α (Biolegend, 100708) at 4 °C for 30 min. Cells were then subjected to IFNγ (Biolegend, 505832), interleukin-2 (IL-2; Biolegend, 503810) and granzyme B (eBioscience, 35-8898-82) staining using a fixation/permeablization kit (BD, 554714) according to the manufacturer’s instructions.

Flow cytometry analysis was performed using a CytoFLEX LX flow cytometer (Beckman Coulter). Data were analyzed with CytExpert 2.4 software. The gating strategies used for surface marker analysis are provided in Supplementary Fig. 1, and strategies for the analysis of intracellular markers (IFNγ, IL-2 and granzyme B) are provided in Supplementary Fig. 2.

### RNA sequencing and analysis pipeline

A375 cells treated with 10 μM peptides for 24 h or expressing sgRNAs targeting *KAT8* were stimulated with 100 U ml^−1^ IFNγ for 6 h. Cells were then collected, and total RNA was extracted using TRIzol (Life Technologies, 15596026). RNA integrity was measured with an Agilent 2100 Bioanalyzer System, and RNA libraries were prepared by poly(A) capture and reverse transcription of cDNA. After quality validation. Libraries were sequenced with 150-base pair paired-end sequencing strategies using an Illumina NovaSeq 6000. For RNA-sequencing data analysis, the pipeline nf-core/rnaseq (v3.8.1)^72,73^ was used. The MHC class I-associated gene list was retrieved from the Reactome class I MHC mediated antigen processing presentation gene set in the the Molecular Signatures Database of gsea-msigdb.org (ref. ^74^). The IRF1 target gene set was downloaded from the Harmonizome^75^ database.

### Statistics and reproducibility

No statistical methods were used to predetermine sample sizes; sample sizes were chosen empirically and are similar to those reported in previous studies. No data were excluded from the analysis. All of the statistical tests used in this study are indicated in the figure legends. For in vivo experiments, all mice were randomly allocated into experimental groups. For cell line-based experiments, randomization was not required because all samples were analyzed equally. The investigators were not blinded to allocation during experiments and outcome assessment. GraphPad Prism software (v8.2) was used for all statistical analyses except for the chi-square test and the correlation analysis of KAT8 and PD-L1 expression in the multiple organ cancer tissue arrays, which were analyzed using SPSS (IBM SPSS Statistics 25). For qPCR and ChIP–qPCR data, the results of three independent experiments were tested using two-tailed Student’s *t*-tests. To test differences in mouse tumor growth, a two-way analysis of variance (ANOVA) followed by Tukey’s multiple comparisons test was used. Data distribution was assumed to be normal, but this was not formally tested.

### Reporting summary

Further information on research design is available in the Nature Portfolio Reporting Summary linked to this article.

## Supplementary information

Supplementary Information Supplementary Figs. 1 and 2. Gating strategies.

Reporting Summary

Supplementary Table Supplementary Tables 1–8.

Supplementary Video 1 optoDroplets assay of the mCherry channel.

Supplementary Video 2 optoDroplets assay of the mEGFP channel.

Supplementary Video 3 optoDroplets assay of the merged channel.

## Source data

Source Data Fig. 1 Unprocessed western blots.

Source Data Fig. 1 Statistical source data.

Source Data Fig. 2 Unprocessed western blots.

Source Data Fig. 2 Statistical source data.

Source Data Fig. 3 Unprocessed western blots.

Source Data Fig. 3 Statistical source data.

Source Data Fig. 4 Statistical source data.

Source Data Fig. 5 Unprocessed western blots.

Source Data Fig. 5 Statistical source data.

Source Data Fig. 6 Unprocessed western blots.

Source Data Fig. 6 Statistical source data.

Source Data Fig. 7 Unprocessed western blots.

Source Data Fig. 7 Statistical source data.

Source Data Fig. 8 Statistical source data.

Source Data Extended Data Fig. 1 Unprocessed western blots.

Source Data Extended Data Fig. 1 Statistical source data.

Source Data Extended Data Fig. 2 Statistical source data.

Source Data Extended Data Fig. 3 Unprocessed western blots.

Source Data Extended Data Fig. 5 Statistical source data.

Source Data Extended Data Fig. 6 Unprocessed western blots.

Source Data Extended Data Fig. 6 Statistical source data.

Source Data Extended Data Fig. 7 Statistical source data.

Source Data Extended Data Fig. 8 Unprocessed western blots.

Source Data Extended Data Fig. 9 Unprocessed western blots.

Source Data Extended Data Fig. 9 Statistical source data.

Source Data Extended Data Fig. 10 Unprocessed western blots.

Source Data Extended Data Fig. 10 Statistical source data.

## Data Availability

RNA-sequencing data that support the findings of this study have been deposited in the Genome Sequence Archive for Humans with accession code HRA003184. Mass spectrometry data have been deposited in the ProteomeXchange Consortium via the iProX partner repository^76,77^ with the accession codes PXD038565 and PXD038568. ChIP–seq data of KAT8 (ENCSR954KIC and ENCFF656USH) and IRF1 (ENCSR890DSP and ENCFF775DML) were retrieved from the ENCODE database (https://www.encodeproject.org/). Source data are provided with this paper. All other data supporting the findings of this study are available from the corresponding author on reasonable request.

## Code availability

No unique code was developed for this study.
