# Author Correction: Disrupting the phase separation of KAT8–IRF1 diminishes PD-L1 expression and promotes antitumor immunity

**DOI:** 10.1038/s43018-026-01218-y

**Published:** 2026-07-16

**Authors:** Yuanzhong Wu, Liwen Zhou, Yezi Zou, Yijun Zhang, Meifang Zhang, Liping Xu, Lisi Zheng, Wenting He, Kuai Yu, Ting Li, Xia Zhang, Zhenxuan Chen, Ruhua Zhang, Penghui Zhou, Nu Zhang, Limin Zheng, Tiebang Kang

**Affiliations:** 1 Sun Yat-sen University Cancer Center, State Key Laboratory of Oncology in South China, Collaborative Innovation Center for Cancer Medicine, Guangzhou, China; 2 Department of Neurosurgery, First Affiliated Hospital of Sun Yat-sen University, Guangzhou, People’s Republic of China

**Keywords:** Gene regulation, Acetylation, Tumour immunology, Cancer therapy, Cancer

Correction to: *Nature Cancer* 10.1038/s43018-023-00522-1, published online 9 March 2023.

In the version of this article originally published, the line scan analyses of TagBFP-KAT8 IRF1 K78R-mCherry in Fig. 5n and the corresponding source data file included the last 30 values of the TagBFP-KAT8 IRF1-mCherry dataset shown in the same figure panel and source data file. This error occurred during graph preparation, because the 519 TagBFP-KAT8 IRF1 K78R-mCherry data points were pasted into a file that already contained the 549 TagBFP-KAT8 IRF1-mCherry data points, thus inadvertently retaining the last 30 data points of TagBFP-KAT8 IRF1-mCherry in the TagBFP-KAT8 IRF1 K78R-mCherry dataset. The amended figure (also shown as Fig. 1, below) and source data are now available in the HTML and PDF versions of the article.

**Fig. 1:**
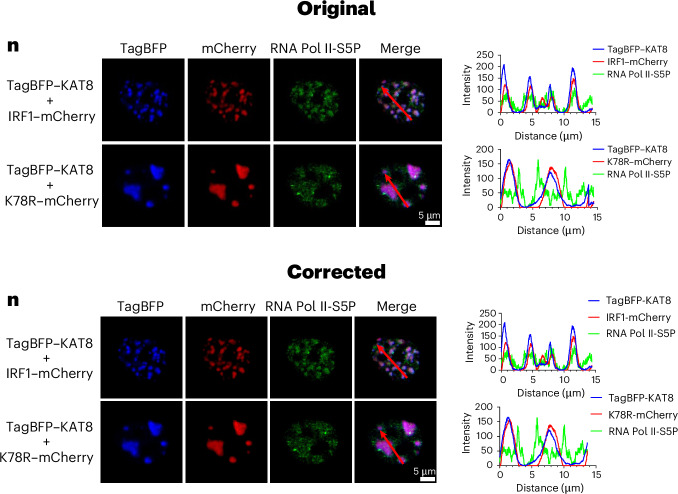
**Original and corrected Fig. 5n.**

In addition, in three instances within the Abstract and main text, “*CD247*” should have been “*CD274*” and in the Methods section on Animal Experiments, “BALB/c” should have been mentioned in the sentence “Six- to 8-week-old C57BL/6N and NOG female mice” that describes the mouse strains used in the experiments. These errors have been amended in the HTML and PDF versions of the article.

