# Supplementary material for: Disrupting the phase separation of KAT8–IRF1 diminishes PD-L1 expression and promotes antitumor immunity
Source: Nat Cancer. 2023 Mar 9;4(3):382–400. doi: 10.1038/s43018-023-00522-1 (PMC10042735; doi:10.1038/s43018-023-00522-1)
Supplement: Supplementary file 1 — Supplementary Figs. 1 and 2. Gating strategies. [file 43018_2023_522_MOESM1_ESM.pdf]

nature cancer

Article

https://doi.org/10.1038/s43018-023-00522-1

Disrupting the phase separation of KAT8–IRF1 diminishes PD-L1 expression and promotes antitumor immunity

In the format provided by the authors and unedited

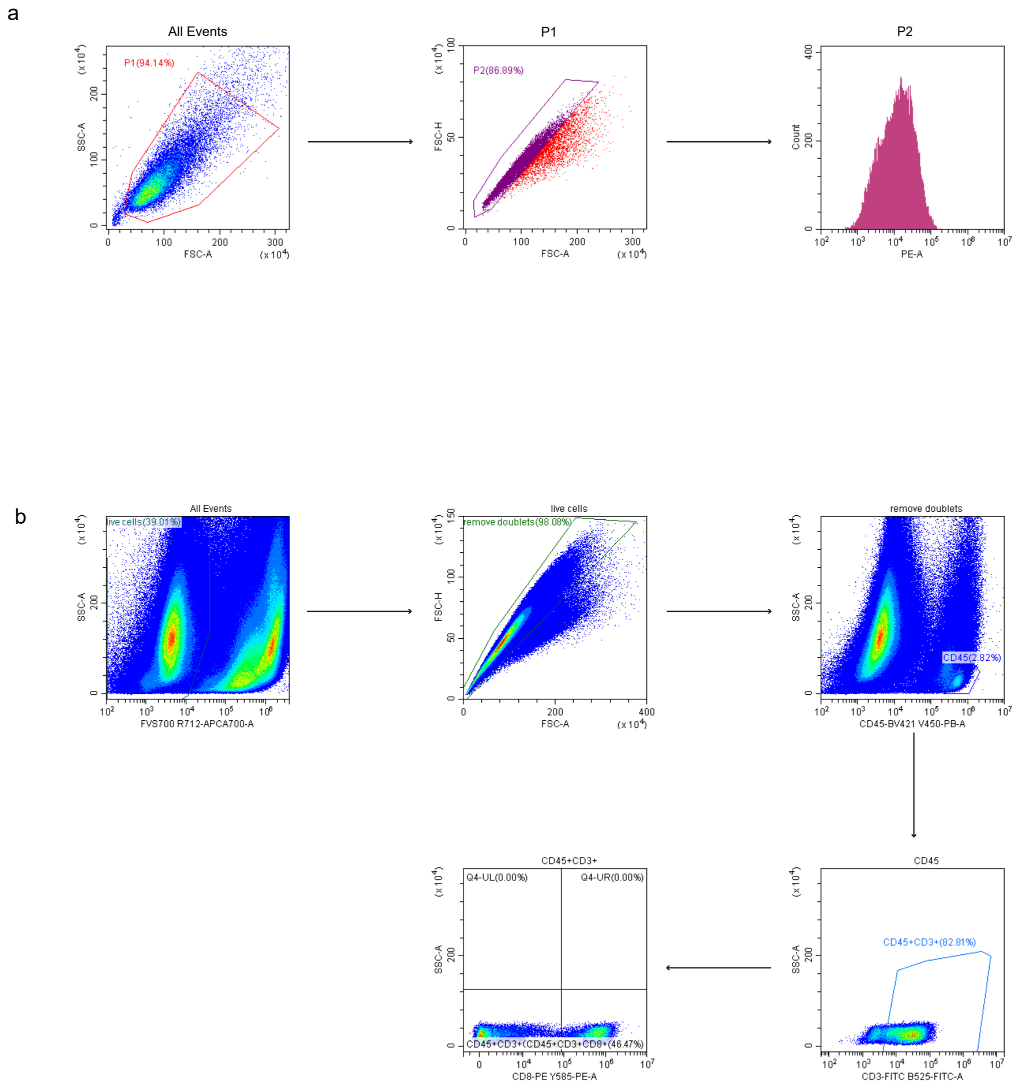

Supplementary Fig. 1 Gating strategies for flow cytometry analysis.

a. Gating strategy for cell surface PD-L1 expression analysis of human cancer cell lines, related to **Extended Data Fig. 1**.

b. Gating strategy for analysis of CD3+CD8+ T cells in tumor-infiltrating lymphocytes, related to **Fig. 1, Fig. 8 and Extended Data Fig. 10**.

a

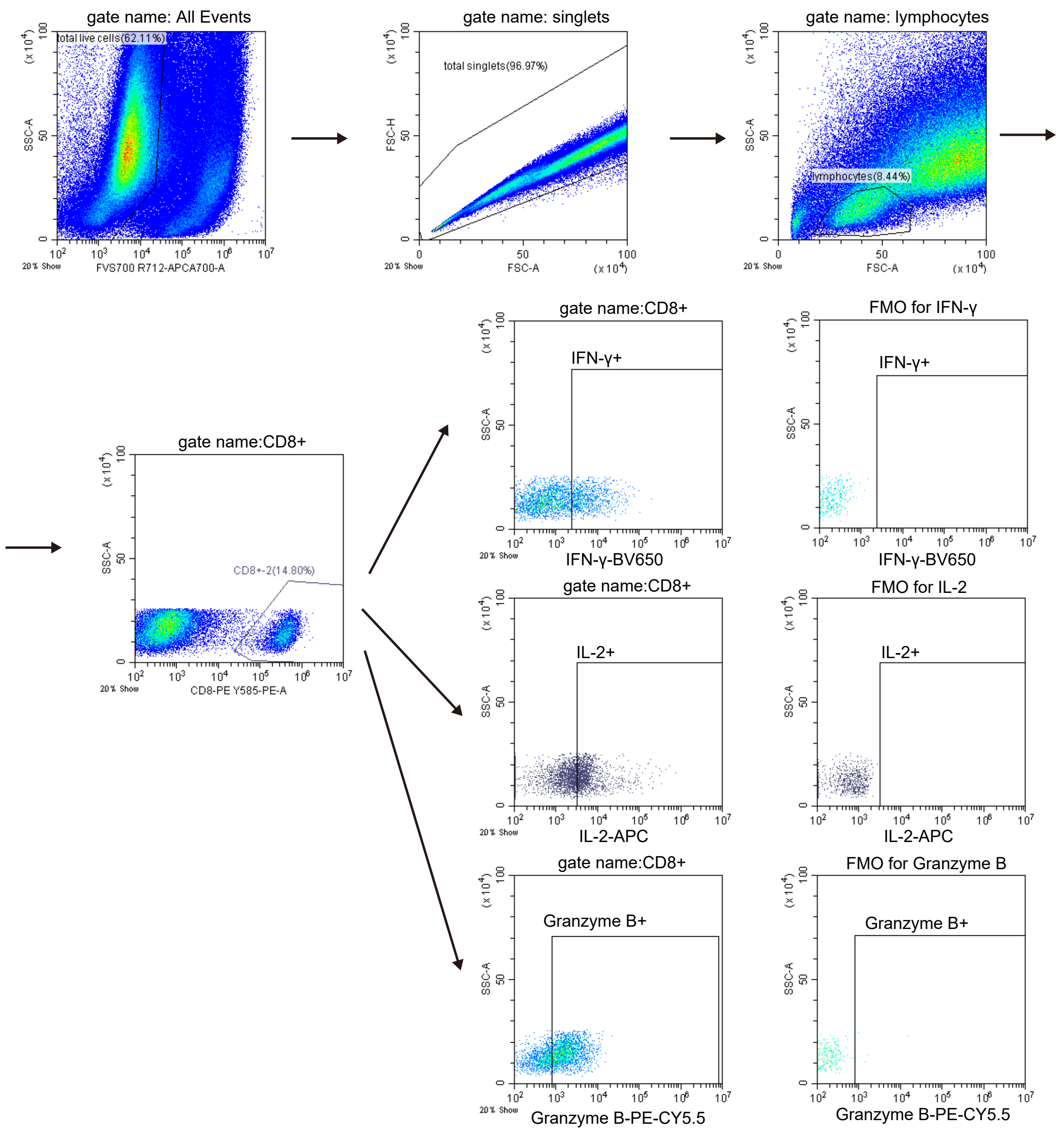

Supplementary Fig. 2 Gating strategies for flow cytometry analysis.

a. Gating strategy for analysis of CD8+IFN-γ+, CD8+IL2+, CD8+GranzymeB+T cells in tumor-infiltrating lymphocytes, related to **Fig. 8 and Extended Data Fig. 10**
