# Supplementary material for: Disrupting the phase separation of KAT8–IRF1 diminishes PD-L1 expression and promotes antitumor immunity
Source: Nat Cancer. 2023 Mar 9;4(3):382–400. doi: 10.1038/s43018-023-00522-1 (PMC10042735; doi:10.1038/s43018-023-00522-1)
Supplement: Supplementary file 2 — Reporting Summary [file 43018_2023_522_MOESM2_ESM.pdf]

nature portfolio

Corresponding author(s): Tiebang Kang

Last updated by author(s): Jan 13, 2023

Reporting Summary

Nature Portfolio wishes to improve the reproducibility of the work that we publish. This form provides structure for consistency and transparency in reporting. For further information on Nature Portfolio policies, see our Editorial Policies and the Editorial Policy Checklist.

Statistics

For all statistical analyses, confirm that the following items are present in the figure legend, table legend, main text, or Methods section.

n/a	Confirmed	
☐	☒	The exact sample size (*n*) for each experimental group/condition, given as a discrete number and unit of measurement
☐	☒	A statement on whether measurements were taken from distinct samples or whether the same sample was measured repeatedly
☐	☒	The statistical test(s) used AND whether they are one- or two-sided<br>*Only common tests should be described solely by name; describe more complex techniques in the Methods section.*
☒	☐	A description of all covariates tested
☒	☐	A description of any assumptions or corrections, such as tests of normality and adjustment for multiple comparisons
☐	☒	A full description of the statistical parameters including central tendency (e.g. means) or other basic estimates (e.g. regression coefficient) AND variation (e.g. standard deviation) or associated estimates of uncertainty (e.g. confidence intervals)
☐	☒	For null hypothesis testing, the test statistic (e.g. *F*, *t*, *r*) with confidence intervals, effect sizes, degrees of freedom and *P* value noted<br>*Give P values as exact values whenever suitable.*
☒	☐	For Bayesian analysis, information on the choice of priors and Markov chain Monte Carlo settings
☒	☐	For hierarchical and complex designs, identification of the appropriate level for tests and full reporting of outcomes
☐	☒	Estimates of effect sizes (e.g. Cohen's *d*, Pearson's *r*), indicating how they were calculated

Our web collection on statistics for biologists contains articles on many of the points above.

Software and code

Policy information about availability of computer code

Data collection	LightCycler480 (Roche), ZEISS LSM 880 confocal laser scanning microscope, ENCODE database, Uniprot database, CytoFLEX LX Flow Cytometer (Beckman Coulter), MoFlo (Beckman Coulter), N-SIM Super Resolution Microscope (Nikon), FV1000 confocal microscopy (OLYMPUS), csu-w1 spinning disk confocal microscopy (Nikon)
Data analysis	GraphPad Prism v8.2, IBM SPSS statistics 25 were used for statistical analysis. Proteome Discoverer 2.2 was used for analyzing the Mass spectrometry raw data by searching the Uniprot database. MaGeCK 0.5.9 was used for raw data analysis of whole-genome CRSIPR-Cas9 gene knockout screens.CytExpert 2.4 was used for flow cytometry data analysis.ZEN 2.3 lite was used for ZEISS LSM880 image.Fiji was used for LLPS related quantification, in vitro cytotoxic assay grayscale value analysis, and the immunofluorescence intensity of in tumor tissue analysis. For RNA-seq data analysis, the pipeline nf-core/rnaseq (v3.8.1) was used. Briefly, reads were aligned to the GRCh37 reference genome using HISAT2 and gene expression was quantified with featureCounts. Deferentially expressed genes were identified using DESeq2. The MHC class I associated gene list was retrieved from the REACTOME_CLASS_I_MHC_MEDIATED_ANTIGEN_PROCESSING_PRESENTATION gene set in the Molecular Signatures Database of GSEA-msigdb.org. The IRF1 target gene set was downloaded from Harmonizome database.

For manuscripts utilizing custom algorithms or software that are central to the research but not yet described in published literature, software must be made available to editors and reviewers. We strongly encourage code deposition in a community repository (e.g. GitHub). See the Nature Portfolio guidelines for submitting code & software for further information.

Data

Policy information about availability of data

All manuscripts must include a data availability statement. This statement should provide the following information, where applicable:

- Accession codes, unique identifiers, or web links for publicly available datasets
- A description of any restrictions on data availability
- For clinical datasets or third party data, please ensure that the statement adheres to our policy

The datasets generated during and/or analyzed during the curent study are available within the main text,extended data and Source data files. RNA–sequencing data that support the findings of this study have been deposited into the Genome Sequence Archive for Humans with accession code HRA003184. Mass spectrometry data have been deposited into ProteomeXchange Consortium via the iProX partner repository with the accession code PXD038565 and PXD038568. ChIP seq data (ENCSR954KIC, ENCFF656USH, ENCSR890DSP, ENCFF775DML) was retrieved from ENCODE database https://www.encodeproject.org/. All other data supporting the findings of this study are available from the corresponding author on reasonable request.

Human research participants

Policy information about studies involving human research participants and Sex and Gender in Research.

Reporting on sex and gender	The findings in our research do not apply to only one sex. Sex was not considered in our human research participants.
Population characteristics	The human multiple organ cancer tissue arrays were purchased from SHANGHAI OUTDO BIOTECH CO., LTD. The information about of the tissue arrays can be found in Supplementary Table 2. For the tumor slices staining, the patient clinical information are as follows: lung cancer patient 1, male, 48 years old; lung cancer patient 2, male, 59 years old, lung cancer patient 3, male, 48 years old; breast cancer patient 1, female, 53 years old; breast cancer patient 2, female, 57 years old; gastric cancer patient 1, female, 83 years old; gastric cancer patient 2, female, 41 years old; melanoma patient 1, male, 71 years old; melanoma patient 1, female, 56 years old. Healthy blood donor: donor 1, female, 44 years old; donor 2, male, 34 years old; donor 3, female, 35 years old; donor 4, female, 28 years old.
Recruitment	The paraffin-embedded tissue slices were obtained from patients who underwent surgical resection at Sun Yat-sen University Cancer Center. We recruited four healthy blood donors, explained this project and answered all their questions in detail.
Ethics oversight	The study design and usage of samples were approved by the Ethics Committee of Sun Yat-sen University Cancer Center (GZKJ2020-019). All the four healthy blood donors in this study signed informed consent. We provided 100 RMB for compensation for each donor.

Note that full information on the approval of the study protocol must also be provided in the manuscript.

Field-specific reporting

Please select the one below that is the best fit for your research. If you are not sure, read the appropriate sections before making your selection.

☒ Life sciences ☐ Behavioural & social sciences ☐ Ecological, evolutionary & environmental sciences

For a reference copy of the document with all sections, see nature.com/documents/nr-reporting-summary-flat.pdf

Life sciences study design

All studies must disclose on these points even when the disclosure is negative.

Sample size	No statistical methods were used to predetermine sample sizes. Sample sizes were determined based on published papers and previous experience, and are described in the Figure legends / Methods. For in vitro studies, a sample size of n=3 would allow for adequate analysis to reach meaningful conclusions of the data. For in vivo studies, a bigger sample size (n=6) was used to compensate for the higher natural variance in vivo.
Data exclusions	No data was excluded from the experiments.
Replication	Our experimental findings were confirmed with at least 3 times independent experiments, unless otherwise indicated. All the experimental findings were reliably reproduced.
Randomization	For in vivo experiments, all mice were randomly allocated into experimental groups. For cell line based experiments, randomization was not required because all samples were analyzed equally.
Blinding	IHC analysis was performed by a pathologist who had no information about patient clinical data and this study. For remaining experiments, no blinding was used.

Reporting for specific materials, systems and methods

We require information from authors about some types of materials, experimental systems and methods used in many studies. Here, indicate whether each material, system or method listed is relevant to your study. If you are not sure if a list item applies to your research, read the appropriate section before selecting a response.

Materials & experimental systems

n/a	Involved in the study
☐	☒ Antibodies
☐	☒ Eukaryotic cell lines
☒	☐ Palaeontology and archaeology
☐	☒ Animals and other organisms
☒	☐ Clinical data
☒	☐ Dual use research of concern

Methods

n/a	Involved in the study
☒	☐ ChIP-seq
☐	☒ Flow cytometry
☒	☐ MRI-based neuroimaging

Antibodies

Antibodies used

anti-human CD274 PE-Cy7 (clone MIH1, BD Biosciences, Cat# 558017, 1:50), anti-human CD274-PE (clone 29E.2A3, Biolegend, Cat# 329706, 1:50), anti-human PD-L1 (GeneTex, Cat# GTX104763, 1:2000 for WB, 1:50 for IHC), anti-human PD-L1 (Clone 405.9A11, Cell Signaling Technology, Cat# 29122S, 1:100 for IF), anti-mouse PD-L1 (Clone D5V3B, Cell Signaling Technology, Cat# 64988, 1:100 for IF), KAT8 (Clone EPR15803, Abcam, Cat# ab200660, 1:1000 for WB, 1:1000 for IHC), KAT8 (Atlas Antibodies, Cat# HPA066324, 1:100 for IF, 1:200 for ChIP), anti-mouse CD8α antibody (clone D4W2Z, Cell Signaling Technology, Cat# 98941; 1:100), IRF1 (Clone D5E4, Cell Signaling Technology, Cat# 8478, 1:2000 for WB, 1:100 for tissue IF), IRF1 (Clone H-8, Santa Cruz, Cat# sc-74530, 1:50 for cell IF), GAPDH (CWBio, Cat# CW0100M, 1:4000), RNA Pol II-S5P (clone 3E8, Millipore, Cat# 04–1572, 1:200), V5 Tag (clone D3H8Q, Cell Signaling Technology, Cat# 13202, 1:2000), FLAG Tag (clone D6W5B, Cell Signaling Technology, Cat# 14793, 1:2000), MYC Tag (Cell Signaling Technology, Cat# 9402, 1:1000), HA Tag (clone 6E2, Cell Signaling Technology, Cat# 2367, 1:2000), Ac-K (Cell Signaling Technology, Cat# 9441, 1:1000), H4K5ac (clone D12B3, Cell Signaling Technology, Cat# 8647, 1:1000), H4K8ac (Cell Signaling Technology, Cat# 2594, 1:1000), H4K12ac (clone D2W6O, Cell Signaling Technology, Cat# 13944, 1:1000), H4K16ac (clone E2B8W, Cell Signaling Technology, Cat# 13534, 1:1000), Histone H4 (clone L64C1, Cell Signaling Technology, Cat# 2935, 1:1000), Fibrillarin (clone C13C3, Cell Signaling Technology, Cat# 2639, 1:200), Coilin (clone IH10, Abcam, Cat# ab87913, 1:200), PML (clone EPR16792, Abcam, Cat# ab179466, 1:200), anti-mouse CD45-BV421 (clone 30-F11, Biolegend, Cat# 103134, 1:50), anti-mouse CD45-APC (Biolegend, Cat# 103112), anti-mouse CD3ε-FITC (clone 145-2C11, Biolegend, Cat# 100306, 1:50), anti-mouse CD3ε-PE (clone 145-2C11, Biolegend, Cat# 100308, 1:50), anti-mouse CD8a-PE (clone 53-6.7, Biolegend, Cat# 100708, 1:50), anti-mouse CD8a-FITC (clone 53-6.7, eBioscience, Cat# 11-0081-82, 1:50), anti-mouse IL-2-APC (clone JES6-5H4, Biolegend, Cat# 503810, 1:50), anti-mouse IFN-γ-Brilliant Violet 650™ (clone XMG1.2, Biolegend, Cat# 505832, 1:50), anti-mouse Granzyme B-PE-Cyanine5.5 (clone NGZB, Cat# 35-8898-82, 1:50), anti-Rabbit IgG (H+L) Highly Cross-Adsorbed Secondary Antibody, Alexa Fluor™ 488 (Invitrogen, Cat# A-21206, 1:1000), anti-Rabbit IgG (H+L) Highly Cross-Adsorbed Secondary Antibody, Alexa Fluor™ Plus 594 (Invitrogen, Cat# A32754, 1:1000), anti-Mouse IgG (H+L) Highly Cross-Adsorbed Secondary Antibody, Alexa Fluor™ 594 (Invitrogen, Cat# A-21203, 1:1000), anti-Mouse IgG (H+L) Cross-Adsorbed Secondary Antibody, Alexa Fluor™ 405 (Invitrogen, Cat# A-31553, 1:1000), anti-Rabbit IgG (H+L) Cross-Adsorbed Secondary Antibody, Alexa Fluor™ 647 (Invitrogen, Cat# A-21244, 1:500), anti-Rat IgG H&L (Alexa Fluor® 488) (Abcam, Cat# ab150157, 1:500), anti-Mouse IgG (H+L)-HRP (Promega, Cat# W4021, 1:50,000), anti-Rabbit IgG (H+L)-HRP (Promega, Cat# W4011, 1:50,000)

Validation

We used the antibodies according to manufacturers' information. For the anti-IRF1-K78ac antibody, we used the IRF1-K78R mutant protein for the validation (Fig. 5b). All other antibodies used in our study have been validated by manufactures and literatures, detailed information could be found on the website from manufactures as listed below:
anti-human CD274 PE-Cy7 (BD Biosciences, Cat# 558017), https://www.bdbiosciences.com/en-us/products/reagents/flow-cytometry-reagents/research-reagents/single-color-antibodies-ruo/pe-cy-7-mouse-anti-human-cd274.558017
anti-human CD274-PE (Biolegend, Cat# 329706), https://www.biolegend.com/nl-nl/products/pe-anti-human-cd274-b7-h1-pd-l1-antibody-4375
anti-human PD-L1 (WB, IHC, GeneTex, Cat# GTX104763), https://www.genetex.com/Product/Detail/PD-L1-antibody/GTX104763
anti-human PD-L1 (IF, Cell Signaling Technology, Cat# 29122S), https://www.cellsignal.com/products/primary-antibodies/pd-l1-405-9a11-mouse-mab/29122
anti-mouse PD-L1 (IF, Cell Signaling Technology, Cat#64988), https://www.cellsignal.com/products/primary-antibodies/pd-l1-d5v3b-rabbit-mab-mouse-specific-ihc-specific/64988
KAT8 (WB, IHC, Abcam, Cat# ab200660), https://www.abcam.com/kat8--myst1--mof-antibody-epr15803-ab200660.html
KAT8 (IF, ChIP, Atlas Antibodies, Cat# HPA066324), https://www.atlasantibodies.com/products/antibodies/primary-antibodies/triple-a-polyclonals/kat8-antibody-hpa066324/
CD8α (IHC, Cat# 98941), https://www.cellsignal.cn/products/primary-antibodies/cd8a-d4w2z-xp-rabbit-mab-mouse-specific/98941?site-search-type=Products&N=4294956287&Ntt=98941&fromPage=plp&_requestid=104328
IRF1 (WB, IF for tissue, Cell Signaling Technology, Cat# 8478), https://www.cellsignal.com/products/primary-antibodies/irf-1-d5e4-xp-rabbit-mab/8478
IRF1 (IF for cell, Santa Cruz, Cat# sc-74530), https://www.scbt.com/zh/p/irf-1-antibody-h-8?requestFrom=search
GAPDH (CWBio, Cat# CW0100M), https://www.cwbio.com/goods/index/id/10116
RNA Pol II-S5P (Millipore, Cat# 04–1572), https://www.merckmillipore.com/DE/en/product/Anti-RNA-polymerase-II-subunit-B1-phospho-CTD-Ser-5-Antibody-clone-3E8,MM_NF-04-1572
V5 Tag (Cell Signaling Technology, Cat# 13202), https://www.cellsignal.com/products/primary-antibodies/v5-tag-d3h8q-rabbit-mab/13202

FLAG Tag (Cell Signaling Technology, Cat# 14793), https://www.cellsignal.com//products/primary-antibodies/dykddddk-tag-d6w5b-rabbit-mab-binds-to-same-epitope-as-sigma-s-anti-flag-m2-antibody/14793
MYC Tag (Cell Signaling Technology, Cat# 9402), https://www.cellsignal.com//products/primary-antibodies/c-myc-antibody/9402
HA Tag (Cell Signaling Technology, Cat# 2367), https://www.cellsignal.com//products/primary-antibodies/ha-tag-6e2-mouse-mab/2367
Ac-K (Cell Signaling Technology, Cat# 9441), https://www.cellsignal.com//products/primary-antibodies/acetylated-lysine-antibody/9441
H4K5ac (Cell Signaling Technology, Cat# 8647), https://www.cellsignal.com//products/primary-antibodies/acetyl-histone-h4-lys5-d12b3-rabbit-mab/8647
H4K8ac (Cell Signaling Technology, Cat# 2594), https://www.cellsignal.com//products/primary-antibodies/acetyl-histone-h4-lys8-antibody/2594
H4K12ac (Cell Signaling Technology, Cat# 13944), https://www.cellsignal.com//products/primary-antibodies/acetyl-histone-h4-lys12-d2w6o-rabbit-mab/13944
H4K16ac (Cell Signaling Technology, Cat# 13534), https://www.cellsignal.com//products/primary-antibodies/acetyl-histone-h4-lys16-e2b8w-rabbit-mab/13534
Histone H4 (Cell Signaling Technology, Cat# 2935), https://www.cellsignal.com//products/primary-antibodies/histone-h4-l64c1-mouse-mab/2935
BV421 anti-mouse CD45 (Biolegend, Cat# 103134), https://www.biolegend.com/en-us/products/brilliant-violet-421-anti-mouse-cd45-antibody-7253?GroupID=BLG6837
anti-mouse CD45-APC (Biolegend, Cat# 103112), https://www.biolegend.com/en-us/products/apc-anti-mouse-cd45-antibody-97
FITC anti-mouse CD3ε (Biolegend, Cat# 100306), https://www.biolegend.com/en-us/products/fitc-anti-mouse-cd3epsilon-antibody-23
anti-mouse CD3ε-PE (Biolegend, Cat# 100308), https://www.biolegend.com/en-us/products/pe-anti-mouse-cd3epsilon-antibody-25?GroupID=BLG6744
anti-mouse CD8α-FITC (eBioscience, Cat# 11-0081-82), https://www.thermofisher.cn/cn/zh/antibody/product/CD8a-Antibody-clone-53-6-7-Monoclonal/11-0081-82
PE anti-mouse CD8a (Biolegend, Cat# 100708), https://www.biolegend.com/en-us/products/pe-anti-mouse-cd8a-antibody-155
Fibrillarin (Cell Signaling Technology, Cat# 2639), https://www.cellsignal.com//products/primary-antibodies/fibrillarin-c13c3-rabbit-mab/2639
Coilin (Abcam, Cat# ab87913), https://www.abcam.com/coilin-antibody-ih10-ab87913.html
PML (Abcam, Cat# ab179466), https://www.abcam.com/pml-protein-antibody-epr16792-ab179466.html
IFN-γ (Biolegend, Cat# 505832), https://www.biolegend.com/fr-ch/products/brilliant-violet-650-anti-mouse-ifn-gamma-antibody-7681
IL-2 (Biolegend, Cat# 503810), https://www.biolegend.com/fr-ch/products/apc-anti-mouse-il-2-antibody-950
GranzymeB (eBioscience, Cat# 35-8898-82), https://www.thermofisher.cn/cn/zh/antibody/product/Granzyme-B-Antibody-clone-NGZB-Monoclonal/35-8898-82

Eukaryotic cell lines

Policy information about cell lines and Sex and Gender in Research

Cell line source(s)	143B (Cat# CRL-8303, ATCC); HCT116 (Cat# CCL-247, ATCC); U20S (Cat# HTB-96, ATCC); HEK293T (Cat# CC4003, Cellcook); A375 (Cat# CC1801, Cellcook); A549 (Cat# CC0202, Cellcook); LLC1 (Cat# CC9044, Cellcook); OVCAR3 (Cat# CC0802, Cellcook); PC9 (Cat# CC0204, CellCook); HCC1937 (Cat# CC0314, Cellcook); PC3 (Cat# CC1202, Cellcook); T24 (Cat# CC1001, Cellcook); DU145 (Cat# CC1201, Cellcook); CT26 (Cat# CC9036, Cellcook); 4T1 (Cat# CC9022, Cellcook); SNU-1040 (Cat# BC2163, Biospes)
Authentication	All cell lines used in this study were authenticated by short tandem repeat DNA finger printing.
Mycoplasma contamination	Cell lines used in this study were routinely tested to be negative for mycoplasma.
Commonly misidentified lines (See ICLAC register)	No commonly misidentified cell lines were used.

Animals and other research organisms

Policy information about studies involving animals; ARRIVE guidelines recommended for reporting animal research, and Sex and Gender in Research

Laboratory animals	Six to eight-week-old C57BL/6N and NOG female mice were purchased from Beijing Vital River Laboratory Animal Technology Co., Ltd. Animal housing rooms temperature; 22 ± 2 Celsius; humidity: 40-70%; 12 hours cycles of light/darkness. Please see the Animal experiments section of Methods for details.
Wild animals	No wild animals were involved in this study.
Reporting on sex	The findings in our research do not apply to only one sex. Sex was not considered in our study design.
Field-collected samples	No field-collected samples were involved in this study.
Ethics oversight	Animal experiments were approved by the Animal Research Committee of Sun Yat-sen University Cancer Center (L102012018110H) and were performed in accordance with established guidelines.

Note that full information on the approval of the study protocol must also be provided in the manuscript.

Flow Cytometry

Plots

Confirm that:

☒ The axis labels state the marker and fluorochrome used (e.g. CD4-FITC).

☒ The axis scales are clearly visible. Include numbers along axes only for bottom left plot of group (a 'group' is an analysis of identical markers).

☒ All plots are contour plots with outliers or pseudocolor plots.

☒ A numerical value for number of cells or percentage (with statistics) is provided.

Methodology

Sample preparation	For analysis of the tumor infiltrated cytotoxic T cells in mice, the resected tumors were first cut into small pieces and incubated in digestion buffer (50 U/mL DNase I and 0.4 mg/mL Collagenase IV in RPMI 1640 medium) at 37°C for 1 h with 100 r.p.m. shaking. After passing through a 70 µm cell strainer, suspension of single cells was washed with PBS three times. BD Horizon™ Fixable Viability Stain 700 (FVS700) (BD, 564997; 1:1000) was utilized to non-viable cell labeling. After the 10 min incubation, FVS700 was washed away with staining buffer (2% FBS in PBS). For experiments of sgKAT8 tumors, cells were stained primary antibodies (anti-CD45 (Biolegend, 103112; 1:50), anti-CD3ε (Biolegend, 100308; 1:50) and anti-CD8α (eBioscience, 11-0081-82; 1:50)) at 4°C for 30 min. For experiments of peptide treatment, cells were stained primary antibodies (anti-CD45 (Biolegend, 103134; 1:50), anti-CD3ε (Biolegend, 100306; 1:50) and anti-CD8α (Biolegend, 100708; 1:50)) at 4°C for 30 min. Stained cells were analyzed by flow cytometry.<br>For analysis of cell surface PD-L1 expression in human cancer cell lines, flow cytometry was performed using anti-CD274 (Biolegend, 329706; 1:50).<br>For intracellular markers analysis, single cells obtained from mice tumors were suspended in RPMI 1640 medium with 10% FBS, at a concentration of about 1×106 cells / mL. The Leukocyte Activation Cocktail (BD, 550583) were added into the medium, at a concentration of 2 µL for 1 mL cell suspension. After incubating at 37°C for 4 h, cells were collected and BD Horizon™ Fixable Viability Stain 700 (FVS700) (BD, 564997; 1:1000) was utilized to non-viable cell labeling. After washing, cells were incubated with anti-CD8α antibodies (Biolegend, 100708; 1:50) at 4°C for 30 min. Then cells were subjected to IFN-γ (Biolegend, 505832; 1:50), IL-2 (Biolegend, 503810; 1:50) and Granzyme B (eBioscience, 35-8898-82; 1:50) staining using Fixation/Permeablization Kit (BD, 554714) according to the manufacturer's instruction.
Instrument	Flow cytometry analysis was performed using CytoFLEX LX Flow Cytometer (Beckman Coulter).
Software	CytExpert 2.4
Cell population abundance	Cell sorting of CRISPR-Cas9 screens were performed using MoFlo Cell Sorter (Beckman Coulter). The sort efficiency was no less than 75% as calculated by MoFlo integrated programme.
Gating strategy	For the CRISPR-Cas9 knockout screens, cells in the total population with the top 5% and tail 5% fluorescence intensity of PD-L1-PE-Cy7 were sorted. For analysis of the cell surface PD-L1 expression of cancer cell lines, cells were first gated by FSC/SSC to exclude debris. The next gate was FSC-A/FSC-H to eliminate non-singlets. Then PD-L1 expression was analyzed by gating PE. For TIL analysis, cells were first gated by FVS700/SSC to exclude non-viable cells. The next gate was FSC-A/FSC-H to eliminate non-singlets. Then gate CD45+ and low SSC-A cell population. Then gate CD3+ polpulation and at last gate CD8+ population.<br>For intracellular marker analysis in CD8 TILs, cells were first gated by FVS700/SSC to exclude non-viable cells. The next gate was FSC-A/FSC-H to eliminate non-singlets. Then cells were gated by FSC/SSC to isolate the lymphocytes population. The next gate is CD8+ cells. Based on this gate, gate IFN-γ, Granzyme B and IL-2, the positive populations were determined by FMO.

☒ Tick this box to confirm that a figure exemplifying the gating strategy is provided in the Supplementary Information.
