# Supplementary figures and images for: Disrupting the phase separation of KAT8–IRF1 diminishes PD-L1 expression and promotes antitumor immunity
Source: Nat Cancer. 2023 Mar 9;4(3):382–400. doi: 10.1038/s43018-023-00522-1 (PMC10042735; doi:10.1038/s43018-023-00522-1)

Fig. 1c

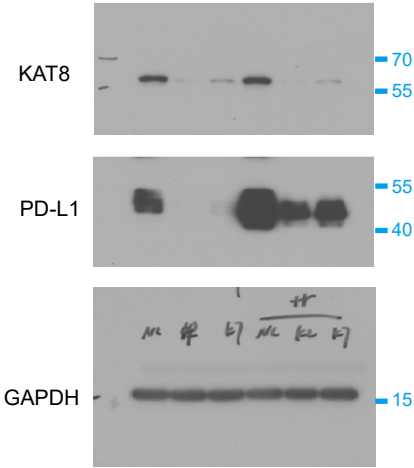

Fig. 1e

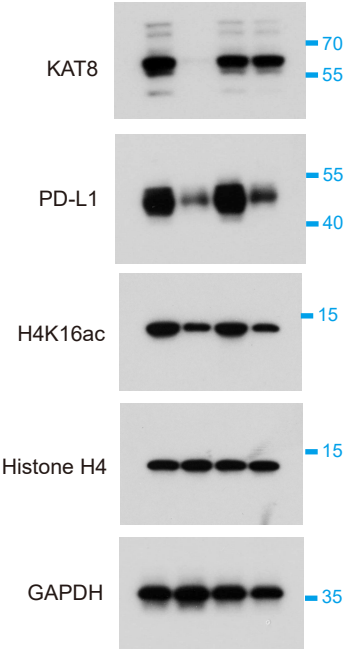

Supplement: Supplementary file 7 — Unprocessed western blots. [file 43018_2023_522_MOESM7_ESM.pdf]

Fig. 2b

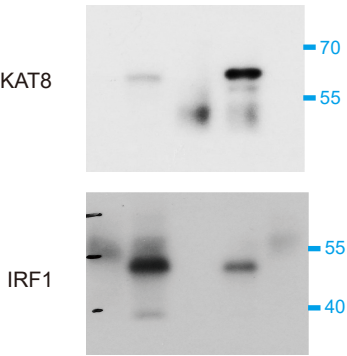

Supplement: Supplementary file 9 — Unprocessed western blots. [file 43018_2023_522_MOESM9_ESM.pdf]

Fig. 3e

IP: FLAG

V5

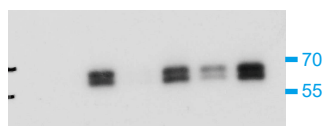

FLAG

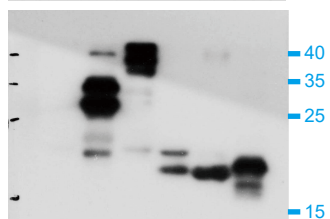

WCL

V5

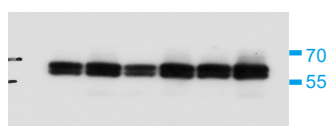

FLAG

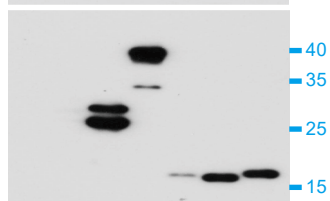

Supplement: Supplementary file 11 — Unprocessed western blots. [file 43018_2023_522_MOESM11_ESM.pdf]

Fig. 5a

IP: Streptavidin

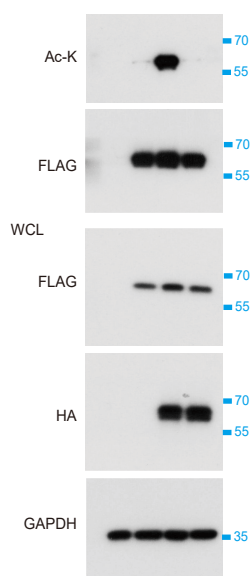

Fig. 5b

IP: Streptavidin

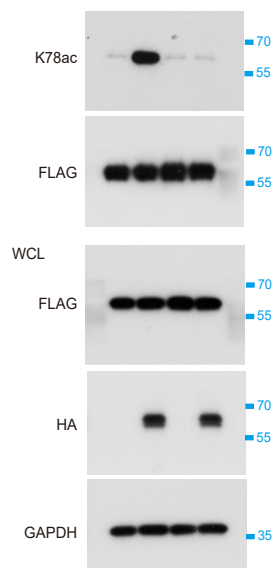

Fig. 5c

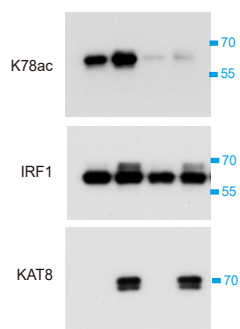

Fig. 5d

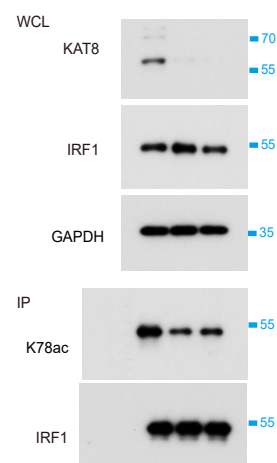

Fig. 5f

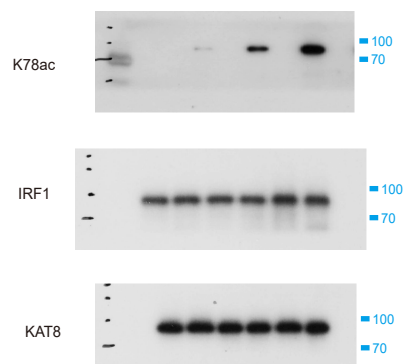

Fig. 5g

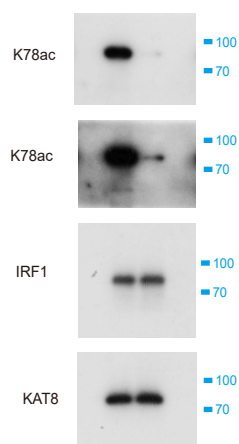

Fig. 5h

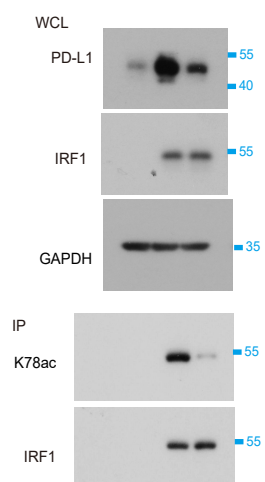

Fig. 5k

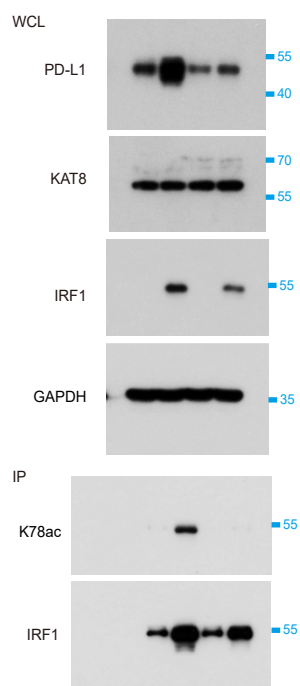

Supplement: Supplementary file 14 — Unprocessed western blots. [file 43018_2023_522_MOESM14_ESM.pdf]

Fig. 6a

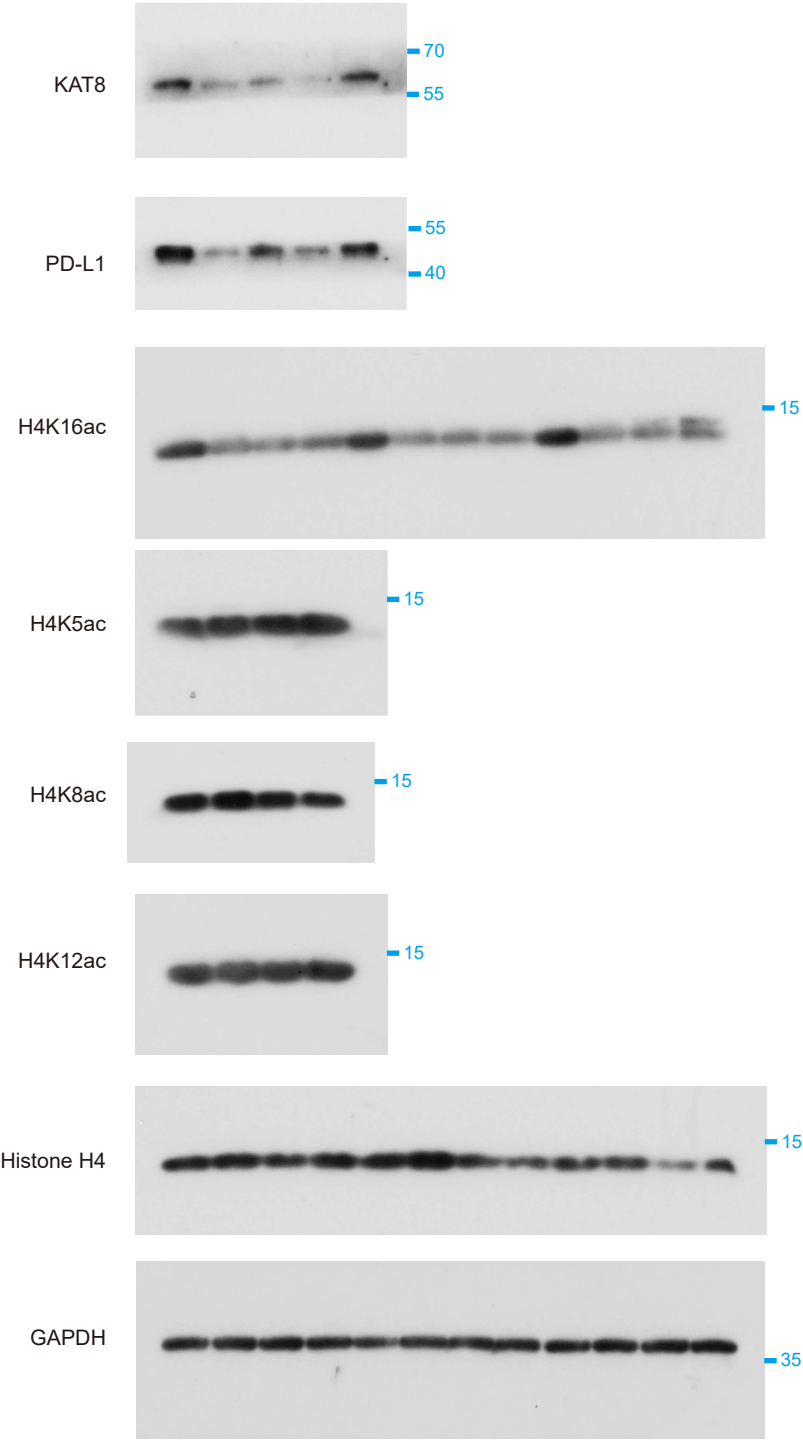

Supplement: Supplementary file 16 — Unprocessed western blots. [file 43018_2023_522_MOESM16_ESM.pdf]

Fig. 7b

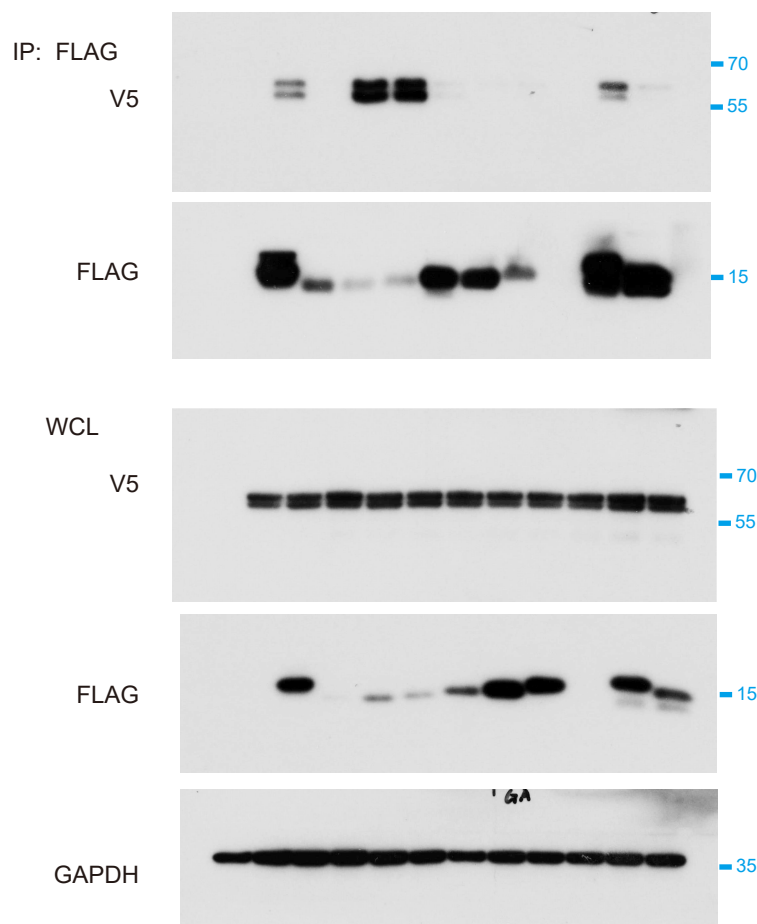

Fig. 7e

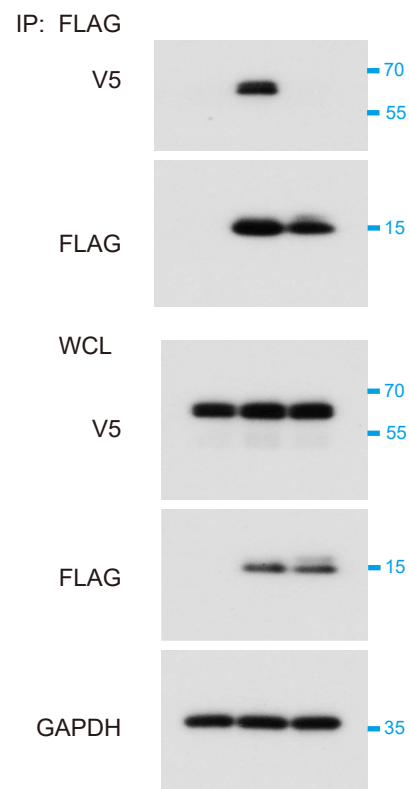

Fig. 7f

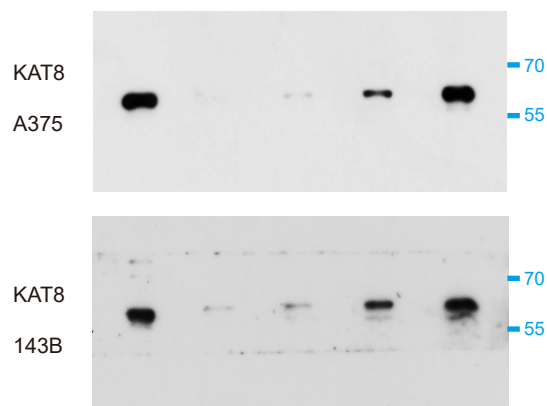

Fig. 7g

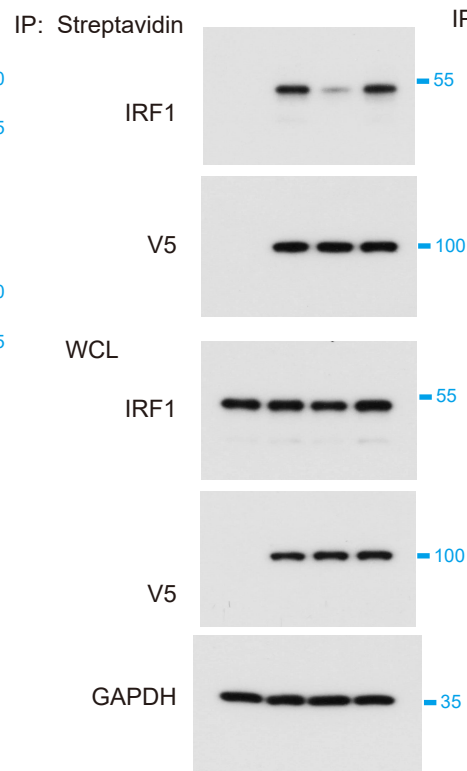

Fig. 7h

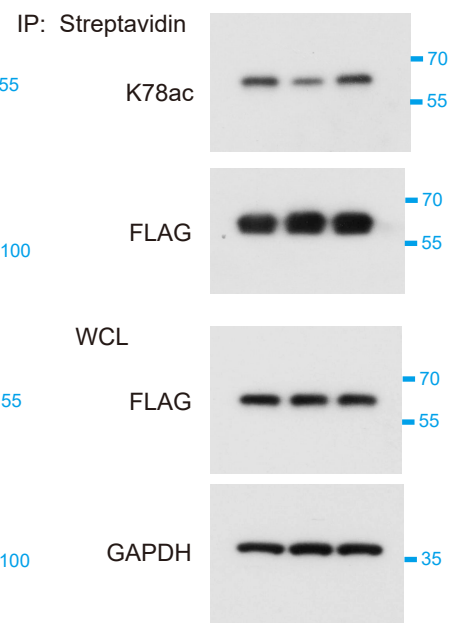

Fig. 7m

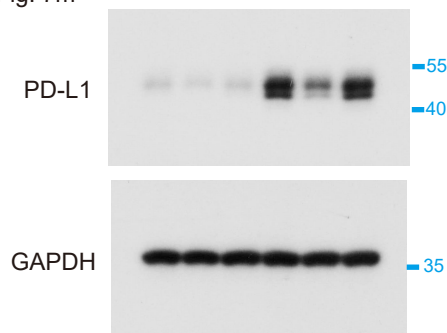

Supplement: Supplementary file 18 — Unprocessed western blots. [file 43018_2023_522_MOESM18_ESM.pdf]

Extended Data Fig. 6c

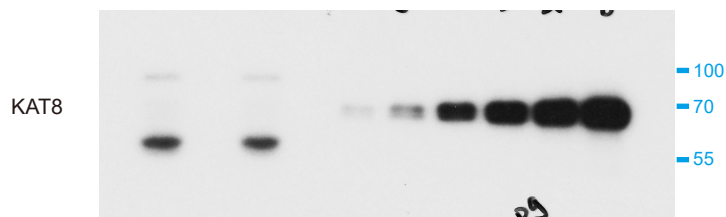

Extended Data Fig. 6d

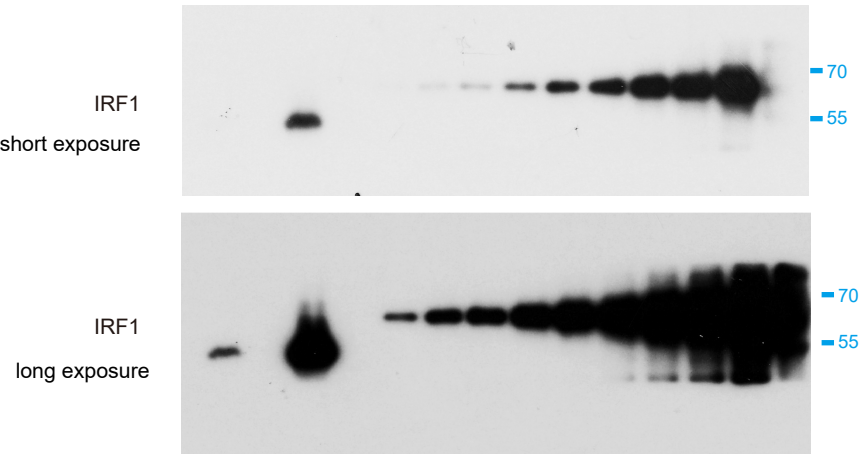

Extended Data Fig. 6e

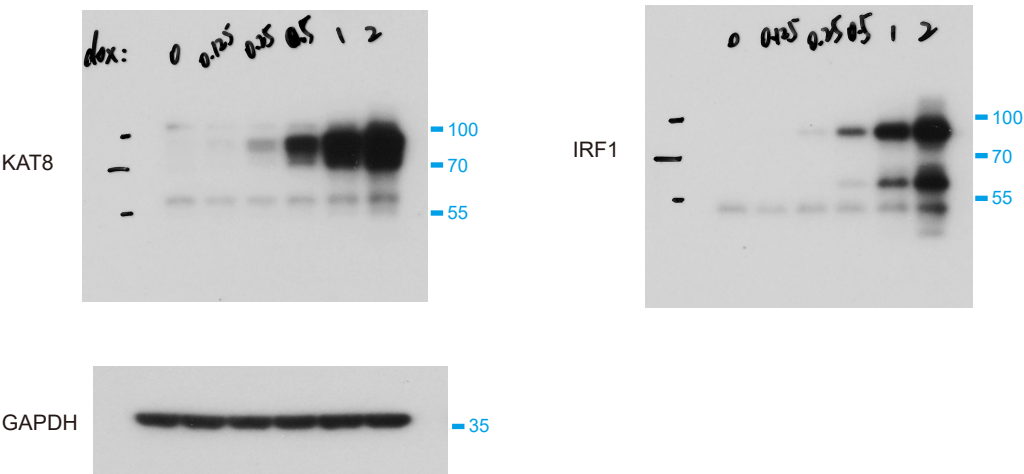

Supplement: Supplementary file 26 — Unprocessed western blots. [file 43018_2023_522_MOESM26_ESM.pdf]

Extended Data Fig. 9b

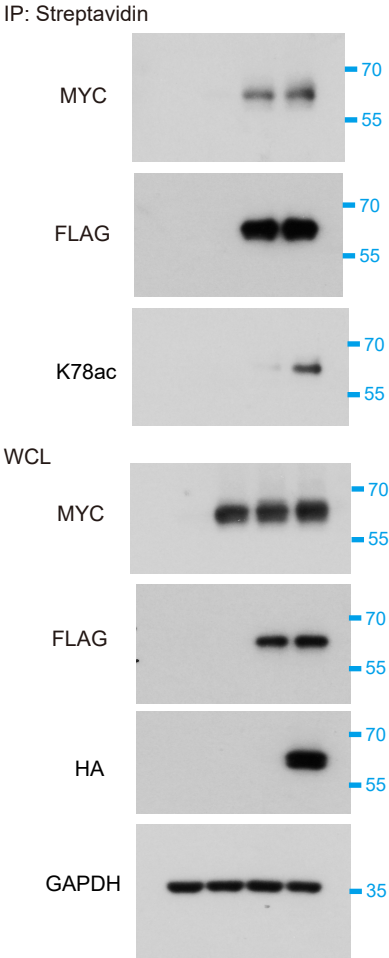

Extended Data Fig. 9i

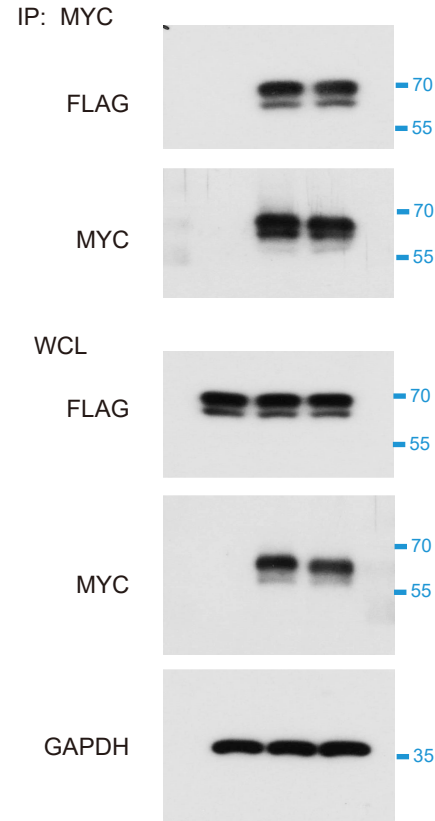

Supplement: Supplementary file 30 — Unprocessed western blots. [file 43018_2023_522_MOESM30_ESM.pdf]
