# Supplementary material for: Disrupting the phase separation of KAT8–IRF1 diminishes PD-L1 expression and promotes antitumor immunity
Source: Nat Cancer. 2023 Mar 9;4(3):382–400. doi: 10.1038/s43018-023-00522-1 (PMC10042735; doi:10.1038/s43018-023-00522-1)

Extended Data Fig. 8a

IP: Streptavidin

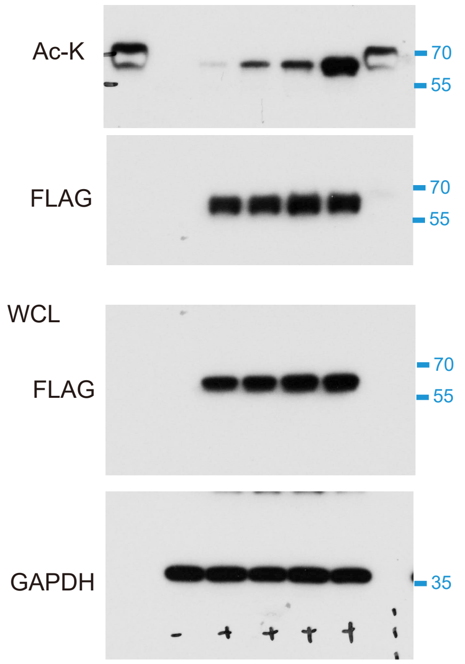

Extended Data Fig. 8c

IP: Streptavidin

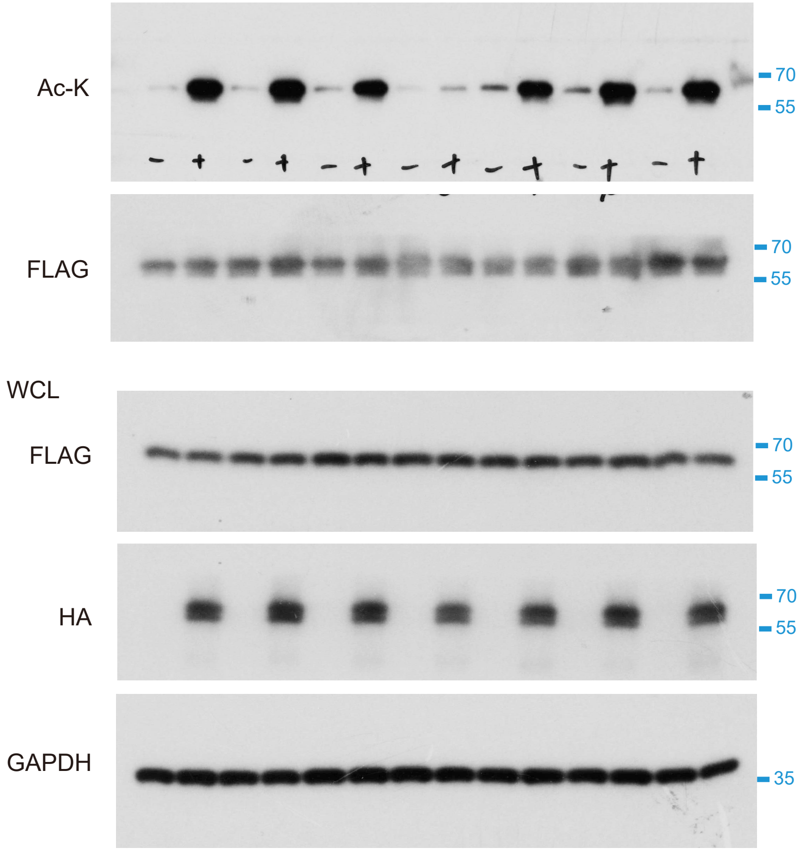

Extended Data Fig. 8d

IP: Streptavidin

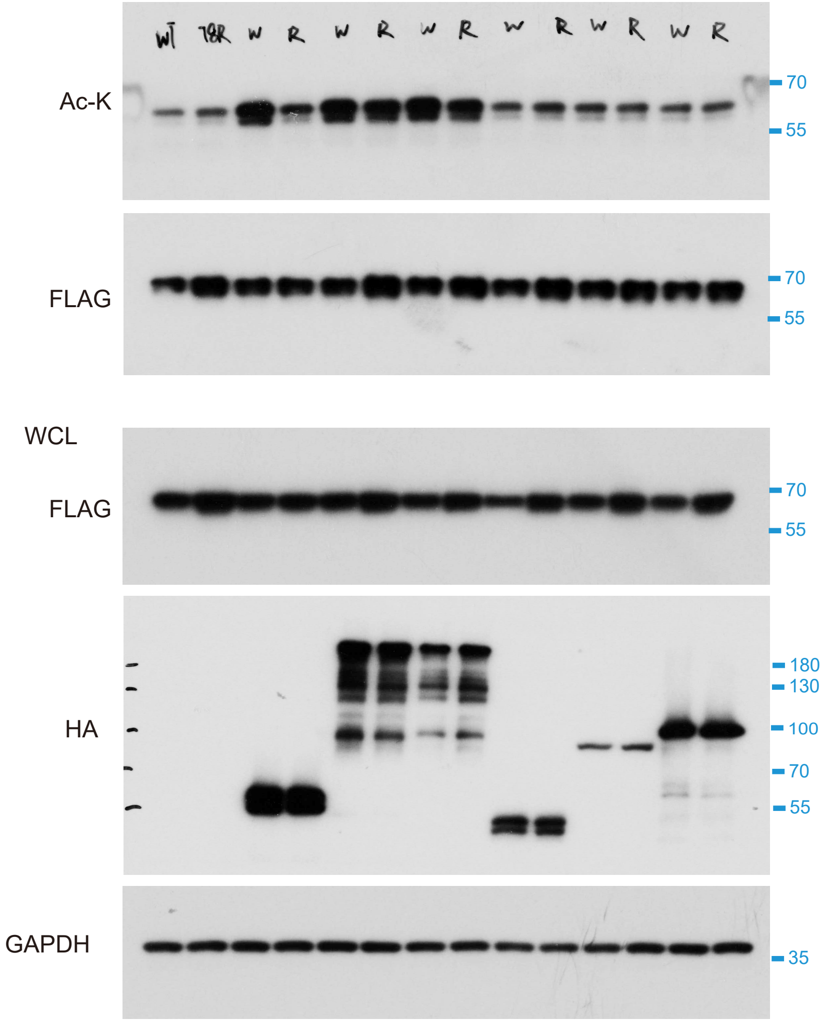

Extended Data Fig. 8e

IP: Streptavidin

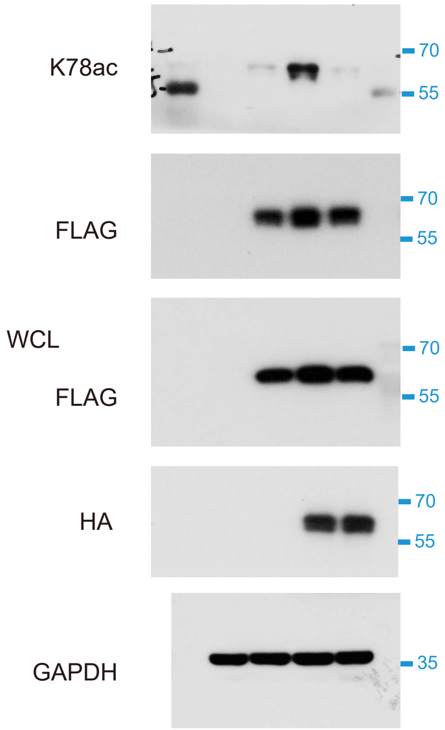

Supplement: Supplementary file 29 — Unprocessed western blots. [file 43018_2023_522_MOESM29_ESM.pdf]
